# Ionizing radiation triggers mitophagy to enhance DNA damage in cancer cells

**DOI:** 10.1038/s41420-023-01573-0

**Published:** 2023-07-28

**Authors:** Yanxian Ren, Pengfei Yang, Chenghao Li, Wen-an Wang, Tianyi Zhang, Jin Li, Haining Li, Chunlu Dong, Wenbo Meng, Heng Zhou

**Affiliations:** 1 Department of General Surgery, The First Hospital of Lanzhou University, Lanzhou, China; 2 School of Public Health, Yangzhou University, Yangzhou, China; 3 Institute of Modern Physics, Chinese Academy of Sciences, Lanzhou, China; 4 Renmin Hospital of Wuhan Economic and Technological Development Zone, Wuhan, China; 5 Gansu Provincial Cancer Hospital, Gansu Provincial Academic Institute for Medical Sciences, Lanzhou, China

**Keywords:** Mitophagy, DNA damage response

## Abstract

Radiotherapy is an important cancer treatment strategy that causes DNA damage in tumor cells either directly or indirectly. Autophagy is a physiological process linked to DNA damage. Mitophagy is a form of autophagy, which specifically targets and eliminates impaired mitochondria, thereby upholding cellular homeostasis. However, the connection between DNA damage and mitophagy has yet to be fully elucidated. We found that mitophagy, as an upstream signal, increases ionizing radiation-induced DNA damage by downregulating or overexpressing key mitophagy proteins Parkin and BNIP3. Enhancing the basal level of mitophagy in conjunction with X-ray irradiation can potentially diminish cell cycle arrest at the G2/M phase, substantially elevate the accumulation of γ-H2AX, 53BP1, and PARP1 foci within the nucleus, augment DNA damage, and facilitate the demise of tumor cells. Consequently, this approach prolongs the survival of melanoma-bearing mice. The findings of this study are anticipated to offer a therapeutic approach for enhancing the therapeutic effectiveness of radiotherapy.

## Introduction

DNA damage is closely related to tumor occurrence and development. In DNA damage response (DDR), radiotherapy activates many signaling pathways, particularly those involving PI3-kinase, such as ataxia telangiectasia and rad3-related protein (ATR) and ataxia telangiectasia mutated (ATM) [1–3]. ATR functions as a protein kinase with specificity for serine and threonine residues, becoming activated upon detection of DNA single-strand breaks (SSBs). When DNA double strand breaks (DSBs) occur, serine/threonine-specific kinase ATM is activated. ATM phosphorylates p53 and modulates p53/Cyclin B1-mediated G2/M phase arrest [4]. Checkpoint kinases Chk1 and Chk2, which undergo phosphorylation and activation by ATR and ATM, have a crucial function in regulating the G2 phase checkpoint and may serve as potential targets for radiotherapy [5–8]. In the absence of exogenous DNA damage, Chk1 kinase is partially phosphorylated, and it is further activated by ATR after DNA damage and replication arrest [9]. Chk2 is abundantly expressed in normal tissues throughout the cell cycle. They are activated by ATM upon DNA damage, which is also the primary pathway for Chk2 activation induced by irradiation [10]. BRCA1 plays a role in all stages of the cell cycle, as well as in the repair of DNA damage. ATR and ATM induce the activation of BRCA1 through the phosphorylation of various serine residues. Upon activation, BRCA1 enhances the activation of ATR/Chk1 and intensifies the G2 phase arrest following irradiation [6]. 53BP1 (p53 binding protein 1) is an intermediary protein of considerable size that possesses the Tudor domain and necessitates its localization to sites of DNA damage. Foci are created through the swift recruitment of 53BP1 in proximity to DNA damage, which is induced by the ATM or ATR-mediated DDR signaling pathway [11]. Moreover, Chk1 promotes the phosphorylation of BRCA1, which is necessary for the proper localization of 53BP1 at the DNA damage site [2]. In the event of DSBs, phosphorylation of histone H2AX at serine-139 (γ-H2AX) occurs at the break site. Phosphorylated γ-H2AX accumulates at DSB sites while simultaneously recruiting various molecules, forming foci, and participating in early DDR [12]. Apart from that, PARP1, a member of the poly (ADP-ribose) polymerase (PARP) family, participates in the recognition of SSBs. PARP inhibitors cause unrepairable SSBs and the collapse of replication forks [13].

As the physiological process that removes damaged proteins and organelles, *sensu stricto* autophagy (often called macroautophagy) is highly conserved throughout eukaryotic evolution, which involves three main stages: the encapsulation of proteins and organelles to form autophagosomes, the fusion of autophagosomes with endosomes to form amphisomes, and the subsequent fusion of amphisomes with lysosomes to form autophagolysosomes [14]. Cell homeostasis and organelle renewal are achieved through the degradation of its contents. Mitophagy is a type of autophagy targeting phagocytosis and degradation of damaged or incomplete mitochondria and is considered an important mechanism controlling the quantity and quality of mitochondria. The PINK1/Parkin signaling pathway mainly regulates mitophagy. PINK1, positioned in the outer membrane of mitochondria, functions as a serine/threonine kinase. Parkin is an E3 ubiquitin ligase located in the cytoplasm. Mitochondrial membrane potential reduction leads to accumulation of PINK1 and the mobilization of Parkin to the outer membrane of mitochondria, resulting in its activation. Once activated, Parkin facilitates the ubiquitination of proteins on the outer mitochondrial membrane, enhances the assembly of autophagosomes, and recruits microtubule-associated protein 1 light chain 3 (LC3). Subsequently, LC3 induces damaged mitochondria to form mitochondrial-autophagosomes through the LC3-interaction region (LIR), accelerating the degradation and clearance of damaged mitochondria [15–17]. Bcl-2 nineteen-kilodalton interacting protein 3 (BNIP3) is a Bcl-2 family protein that possesses a single transmembrane domain and specifically localizes to the outer mitochondrial membrane. Atypical BH3 domains distinguish it from other proteins. BNIP3 upregulates mitophagy levels. It can compete with Beclin-1 for the binding site of the BH3 domain, destroying the stable binding of Bcl-2 and Beclin-1 and allowing Beclin-1 to dissociate and initiate mitophagy. In addition, BNIP3 can interact with LC3 and connect to the mitochondrial membrane to mediate mitophagy [18–20]. BNIP3 gene knockout in the ischemic-reperfusion renal proximal tubular epithelial cell model inhibits mitophagy and protects tubular cells from injury [21].

DNA damage serves as the fundamental principle underlying tumor radiotherapy. The efficacy of radiation in eliminating tumor cells is attributed to its ability to disrupt DNA integrity, while the resistance of tumors to radiation therapy is linked to impaired DNA damage repair mechanisms. When DNA is damaged, it sets off a cascade of DDR designed to aid cell survival, including the induction of autophagy [22]. We assume that autophagy is linked to tumor development and radioresistance. Ionizing radiation can induce mitophagy and stimulate metabolic activation within the mitochondria, providing energy for the DDR. The precise role of mitophagy in radiation-induced DNA damage, however, remains to be determined. Thus, we conducted a study to examine the impact of mitophagy on DNA damage in tumor cells, aiming to propose a novel avenue of enhancing the efficacy of radiotherapy.

## Results

### Ionizing radiation inhibits cell proliferation

**Fig. 1:**
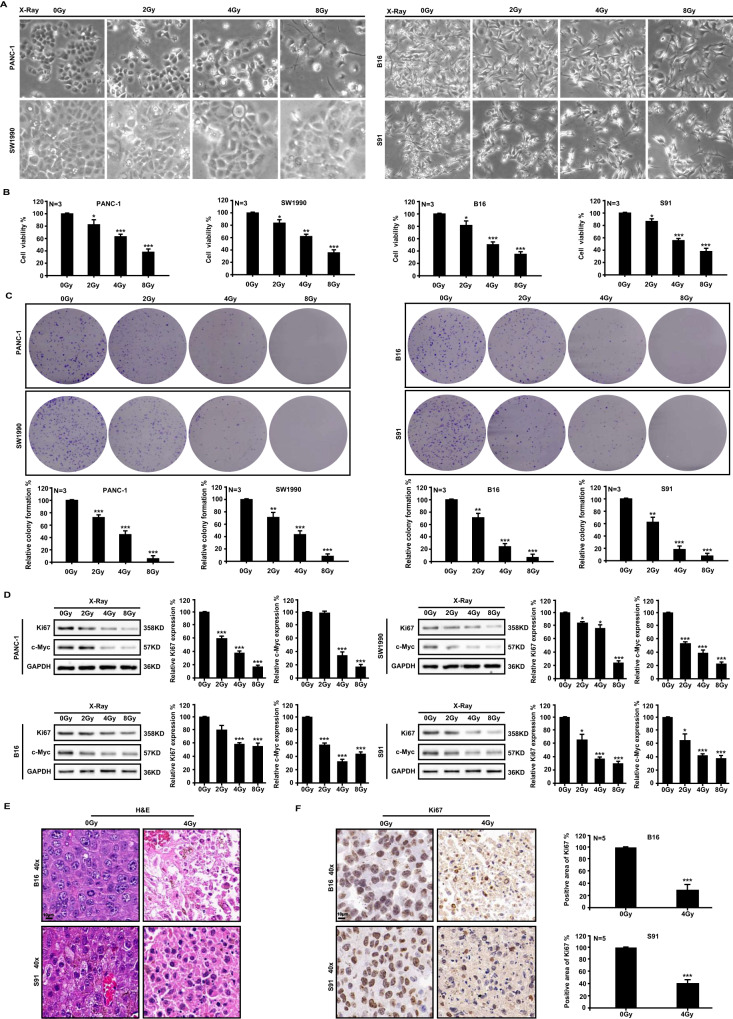
Ionizing radiation inhibits cell proliferation. At 48 h after X-ray irradiation, cell morphology (**A**), survival (**B**), and clonal formation rate (**C**) of human pancreatic cancer cell lines PANC-1 and SW1990, as well as murine melanoma cell lines B16 and S91, were determined. Western blot (**D**), hematoxylin-eosin staining (**E**), and immunohistochemical analyses (**F**) were used to detect the expression levels of the malignant proliferative protein markers Ki67 and c-Myc in cells and tumors.

Human pancreatic cancer cell lines PANC-1 and SW1990, along with murine melanoma cell lines B16 and S91, were subjected to X-ray irradiation at doses of 2 Gy, 4 Gy, and 8 Gy. An increase in radiation dose decreased proliferation and increased cell volume, and the cells were flat and swollen (Fig. 1A). The CCK8 assay indicated a decline in cell viability after 48 h of irradiation, as shown in Fig. 1B. Additionally, Fig. 1C demonstrated a significant decrease in the rate of clone formation. All effects of irradiation were dependent on the dose. Next, we detected Ki67 and c-Myc, two markers of tumor cell malignancy, and found that different doses of ionizing radiation could significantly reduce their expression in the four cell lines (Fig. 1D). Furthermore, 4 Gy X-ray irradiation reduced the size of nuclei (Fig. 1E) and Ki67 expression (Fig. 1F) of B16 and S91 melanoma tumors. As a result, ionizing radiation prevents tumor cell proliferation.

### Ionizing radiation activates DDR signaling pathways

**Fig. 2:**
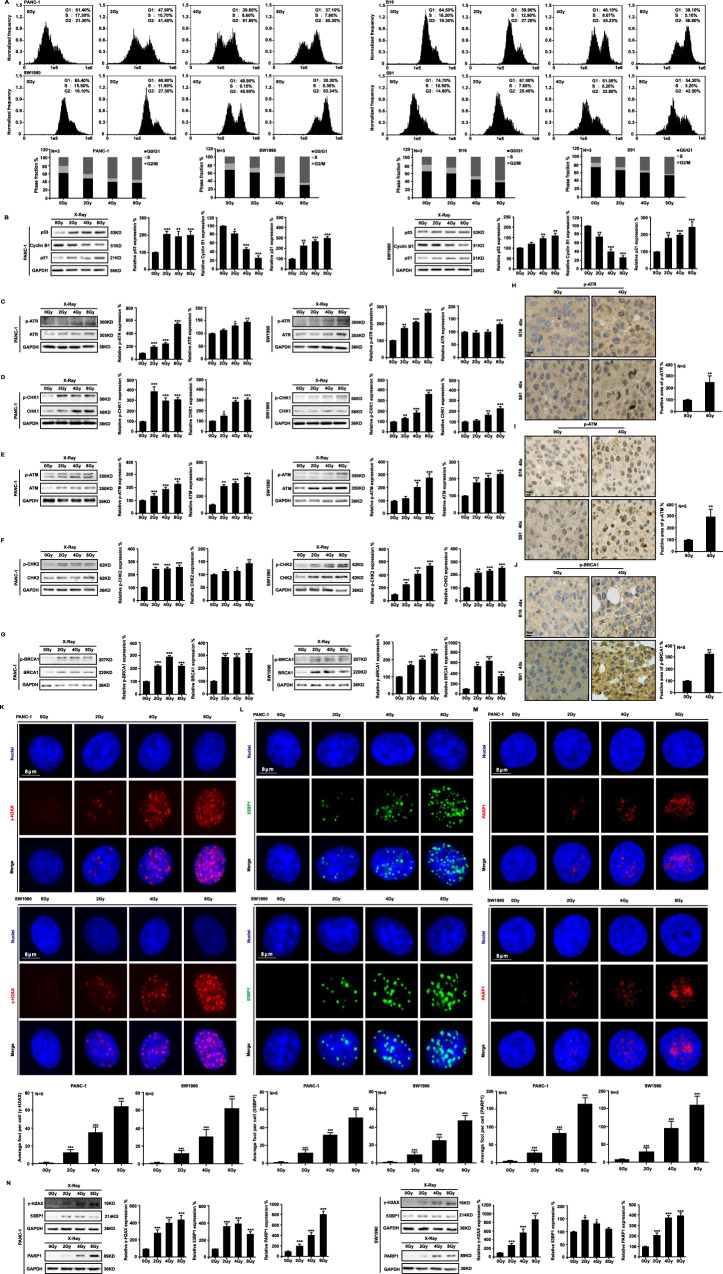
Ionizing radiation activates DNA damage response signaling pathways. Cell cycle arrest was observed at 48 h after X-ray irradiation in human pancreatic cancer cell lines PANC-1 and SW1990, as well as murine melanoma cell lines B16 and S91 (**A**). Western blot analysis was performed to quantify the expression of p53/CyclinB1/p21 proteins (**B**). ATR/Chk1, ATR/Chk2, and BRCA1 expression was detected in cells (**C**–**G**) and tumors (**H**–**J**). Immunofluorescence (**K**–**M**) and western blot (**N**) analyses were conducted to detect the formation of foci and expression levels of DNA damage sensors γ-H2AX, 53BP1, and PARP1.

Ionizing radiation primarily causes nuclear damage. Irradiation with 2 Gy, 4 Gy, and 8 Gy X-rays induced significant G2/M phase arrest at 48 h in PANC-1, SW1990, B16, and S91 cell lines (Fig. 2A). p53 mediates long-term G2 to M phase block maintenance by directly inhibiting Cyclin B1 expression via transcription products, while its downstream gene p21 is also involved in G2/M phase arrest. X-ray irradiation significantly upregulated p53 and p21 expression but downregulated Cyclin B1 expression at 48 h in PANC-1 and SW1990 cells (Fig. 2B). The classical DNA damage repair pathways ATR/Chk1 and ATM/Chk2 were investigated for their responses. There was an increased expression and phosphorylation of ATR, Chk1, ATM, and Chk2 in PANC-1 and SW1990 cells after ionizing radiation was applied (Fig. 2C–F), as well as an increase in phosphorylation of BRCA1 (Fig. 2G). In addition, the phosphorylation levels of ATR, ATM, and BRCA1 were significantly increased after 4 Gy X-ray irradiation in B16 and S91 melanoma tumors (Fig. 2H–J). According to immunofluorescence staining (Fig. 2K–M) and western blot analysis (Fig. 2N), the expression levels of γ-H2AX, 53PB1, and PARP1 in PANC-1 and SW1990 cell lines were dramatically increased at 48 h in a radiation dose-dependent manner. As a result of the above results, it can be concluded that ionizing radiation caused single-stranded or double-stranded DNA damage and induced G2/M phase arrest.

### Ionizing radiation induces mitophagy in tumor cells

**Fig. 3:**
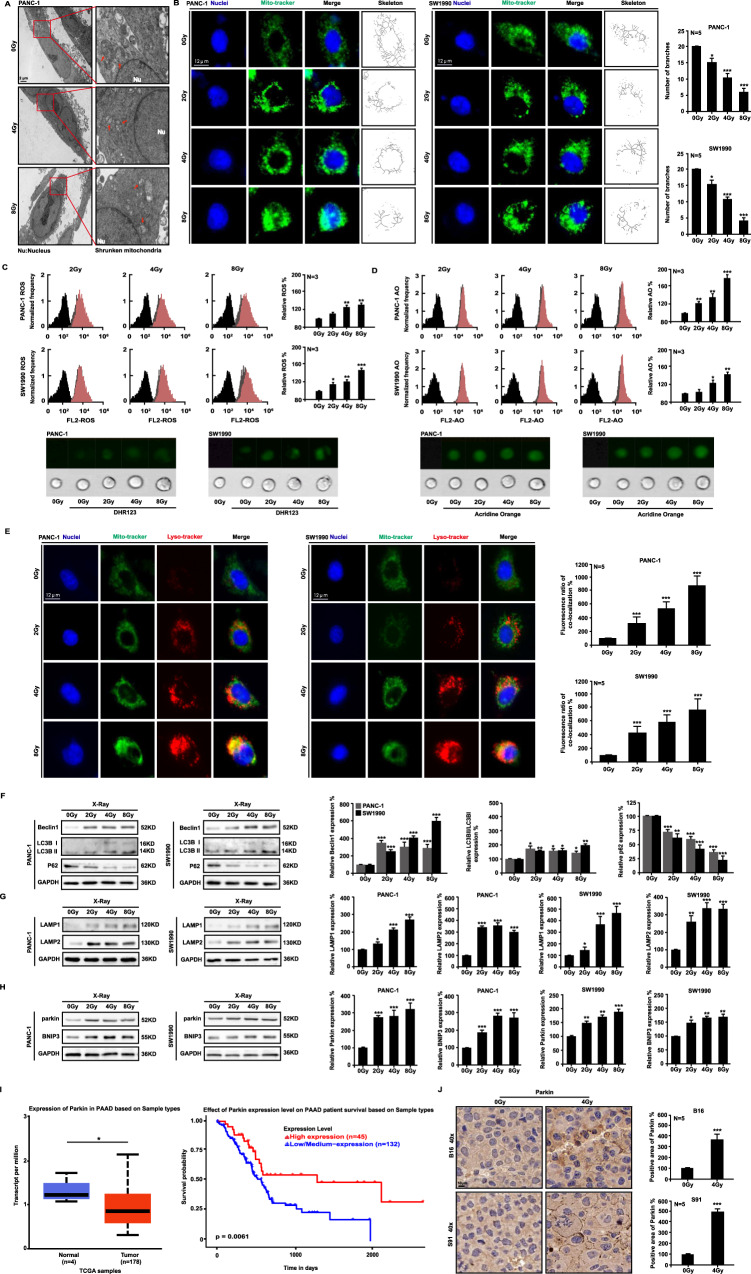
Ionizing radiation induces mitophagy in tumor cells. Transmission electron microscopy (**A**) and immunofluorescence (**B**) analyses were used to examine the morphological changes in mitochondria of PANC-1 cells exposed to X-ray irradiation. Flow cytometry was used to quantify the amount of ROS (**C**) in mitochondria and cell acidification (**D**). Immunofluorescence assay revealed the co-localization of mitochondria and lysosomes in cells exposed to X-ray irradiation at 48 h (**E**). The expression of mitophagy-related proteins was quantified by western blot analyses (**F**–**H**). The TCGA database was used to compare Parkin expression levels versus pancreatic tumor survival rates (**I**). Parkin expression after X-ray irradiation in mouse melanoma (**J**) was detected by immunohistochemical staining.

Ionizing radiation also causes cytoplasm damage. Mitochondria, the powerhouse of cells, are easily damaged by ionizing radiation. Thus, transmission electron microscopy showed that in PANC-1 cells irradiated with 4 Gy or 8 Gy X-ray, the mitochondria were swollen and deformed, with broken cristae, indicating a vacuolated state (Fig. 3A). When PANC-1 and SW1990 cells were X-ray irradiated, immunofluorescence staining showed shrinkage of both branched and unbranched cristae (Fig. 3B). Furthermore, DHR123 staining was used to detect an increase in ROS in the mitochondria of PANC-1 and SW1990 cells after irradiation (Fig. 3C). AO staining was used to detect an increase in acidification level in the two cell lines (Fig. 3D). In PANC-1 and SW1990 cells, immunofluorescence analysis showed that ionizing radiation significantly increased mitochondrial co-location with lysosomes after 48 h (Fig. 3E). Ionizing radiation significantly increased the expression of Beclin1, upregulated autophagy-related LC3 II/LC3 I ratio, decreased p62 level (Fig. 3F), significantly increased lysosomal surface marker proteins LAMP1 and LAMP2 (Fig. 3G), and increased the levels of mitophagy sensors Parkin and BNIP3 (Fig. 3H). Thus, ionizing radiation was found to activate the mitochondrial-lysosome degradation pathway. Our study found that pancreatic tumors expressed higher levels of Parkin than normal tissues based on the TCGA database. Patients with pancreatic tumors with high Parkin expression outlived those with low Parkin expression (Fig. 3I). Similarly, 4 Gy X-ray irradiation significantly increased Parkin expression levels in murine melanoma tumors (Fig. 3J). The preceding experiments demonstrate that ionizing radiation induces mitochondrial damage and triggers mitophagy.

### Parkin/BNIP3-mediated mitophagy promotes DNA damage in vitro

**Fig. 4:**
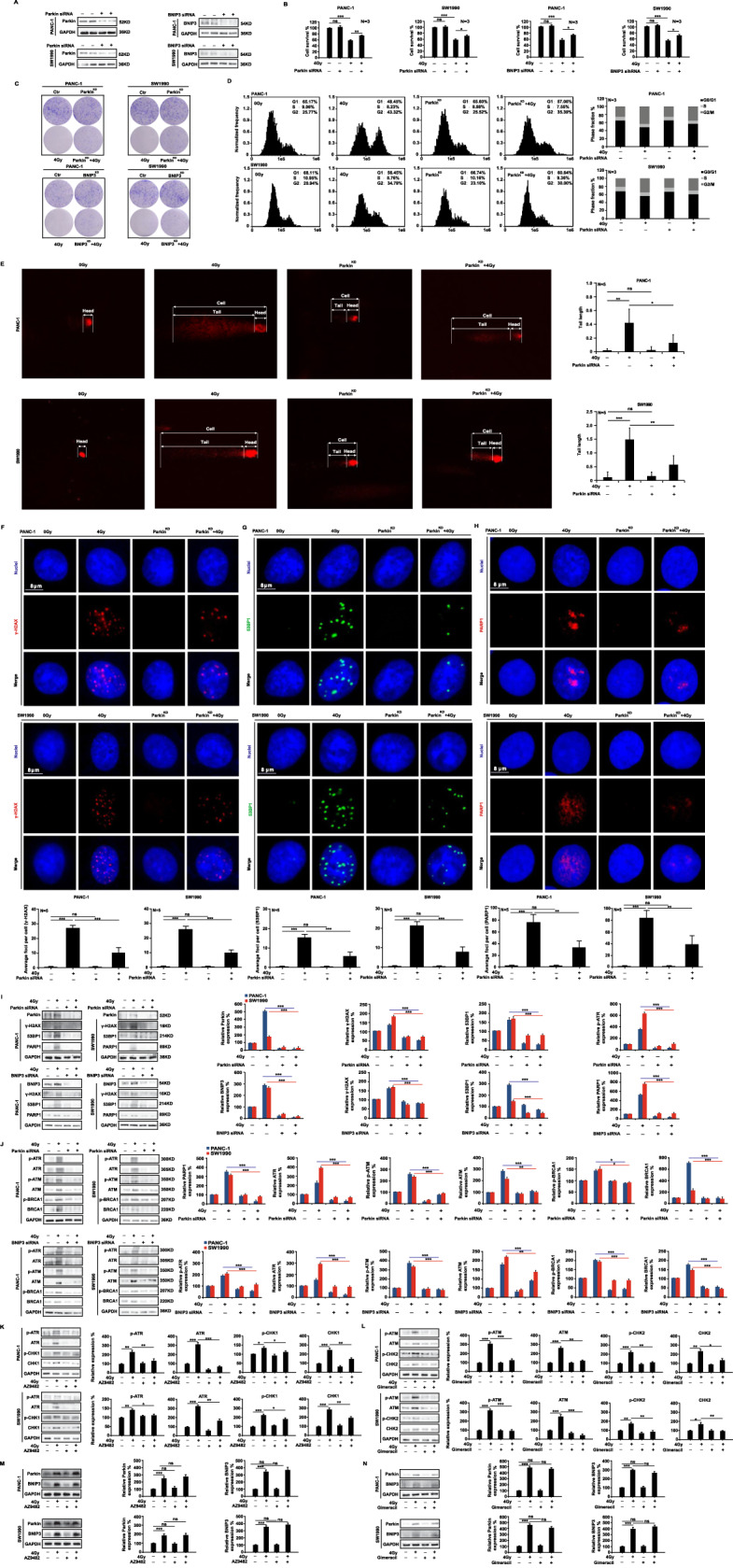
Parkin/BINP3-mediated mitophagy promotes DNA damage in vitro. Parkin^KD^ and BNIP3^KD^ lines of PANC-1 and SW1990 cells were generated by siRNA interference technology (**A**). CCK8 assay (**B**) and clone formation assay (**C**) were used to detect the cell proliferation after 4 Gy X-ray irradiation, flow cytometry (**D**) and comet assay (**E**) was used to detect cell cycle arrest and DNA damage at 48 h after irradiation. Immunofluorescence assay (**F**–**H**) and western blot analysis (**I**) were used to quantify the expression of γ-H2AX, 53BP1, and PARP1, as well as the activation of the ATR/ATM signaling pathway (**J**). The cells were treated with the SSB inhibitor AZ9482 (1 nM) and the DSB inhibitor Gimeracil (1 mM), and mitophagy level changes were observed using western blot analysis (**K**–**N**).

**Fig. 5:**
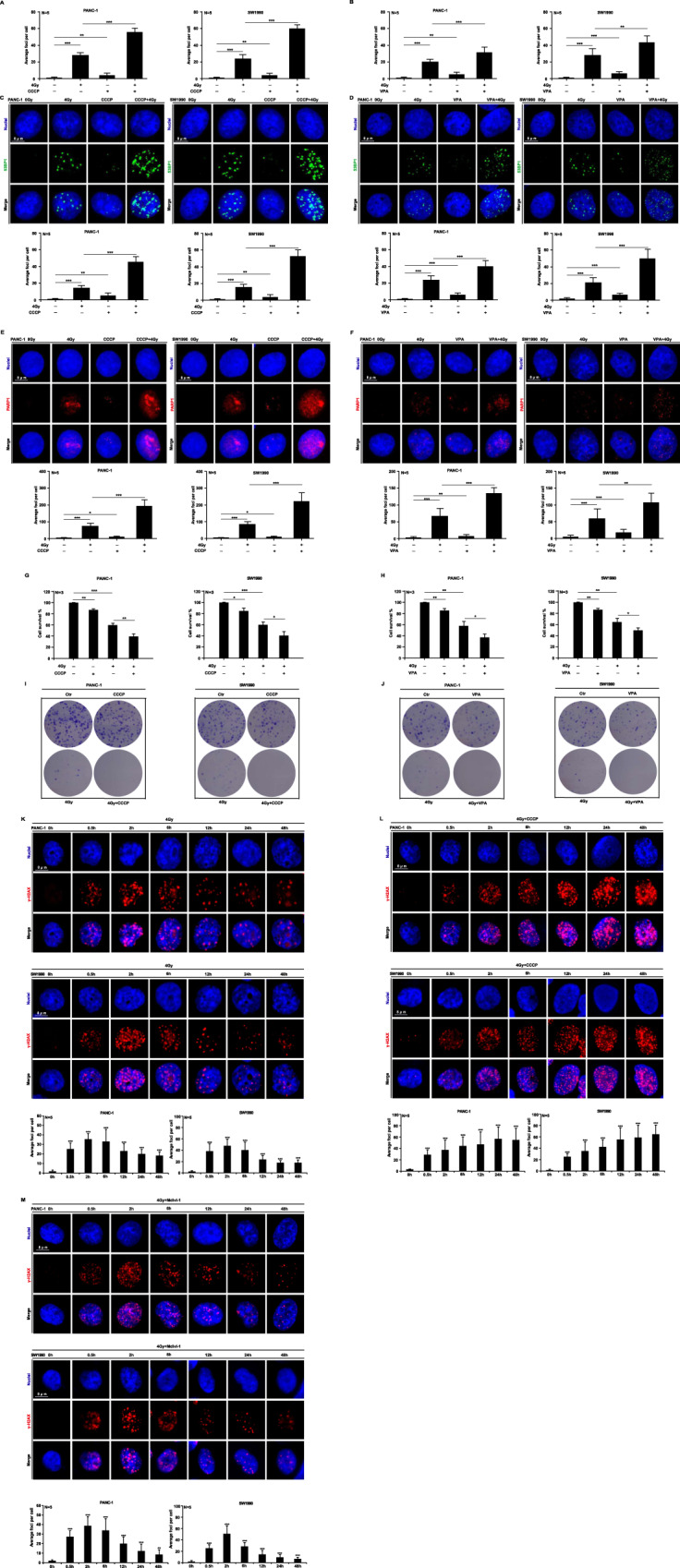
Mitophagy activator enhances ionizing radiation-induced DNA damage. PANC-1 and SW1990 cells were treated with 1 μM CCCP and 1 mM VPA to enhance the baseline level of mitophagy. Immunofluorescence analysis was employed to evaluate the expression of DNA damage sensors, including γ-H2AX (**A**, **B**), 53BP1 (**C**, **D**), and PARP1 (**E**, **F**), in combination with 4 Gy X-ray irradiation. Cell proliferation inhibition was assessed using the CCK8 assay and clonal formation assay (**G**–**J**). Immunofluorescence was utilized to detect γ-H2AX foci at various time points (ranging from 0.5 h to 48 h) following 4 Gy irradiation and combined treatment with CCCP or Mdivi-1 (**K**–**M**).

For investigating the relationship between DNA damage and mitophagy, we chose Parkin and BNIP3, two key proteins in mitophagy. First, we used siRNA interference technology to knock down Parkin (Parkin^KD^) or BINP3 (BNIP3^KD^) expression in PANC-1 and SWA1990 cells (Fig. 4A) and then irradiated the cells with 4 Gy X-ray. Knocking down the expression of the two genes did not affect cell proliferation under normal culture conditions after 48 h. However, Parkin^KD^ or BNIP3^KD^ cells were significantly less likely to die than wild-type cells after irradiation (Fig. 4B). The clonal formation in Parkin^KD^ or BNIP3^KD^ cells after ionizing radiation was significantly augmented when compared to wild-type cells (Fig. 4C). In Parkin^KD^ cells, 4 Gy irradiation resulted in a slight decrease in G2/M phase arrest (Fig. 4D). Moreover, the comet assay demonstrated significantly reduced DNA damage in PANC-1 and SW1990 Parkin^KD^ cells compared to wild-type cells following 4 Gy irradiation (Fig. 4E). Meanwhile, ionizing radiation significantly decreased the levels of DNA damage sensors γ-H2AX, 53PB1 and PARP1 in Parkin^KD^ and BNIP3^KD^ cells compared to wild-type cells (Fig. 4F–H). Western blot assay showed that knocking down Parkin and BNIP3 genes inhibited the expression of γ-H2AX, 53PB1, and PARP1 (Fig. 4I), as well as the ATM/ATR-mediated DNA damage signaling pathway in PANC-1 and SW1990 cells (Fig. 4J). Furthermore, we used the SSB inhibitor AZ9482 (1 nM) (Fig. 4K) and the DSB inhibitor Gimeracil (1 mM) (Fig. 4L) and found that neither inhibitor alone nor the combination of 4 Gy X-ray did not induce abnormal expression changes in Parkin and BNIP3 proteins (Fig. 4M, N). In the presence of the mitophagy activator carbonyl cyanide 3-chlorophenylhydrazone (CCCP) and valproic acid (VPA), Compared with the levels of these proteins when CCCP, VPA, or ionizing radiation were used alone (Fig. 5A–F), 4 Gy X-ray irradiation significantly increased their expression. Through CCK8 and clonal formation assays, combination treatments of mitophagy activator and ionizing radiation inhibited PANC-1 and SW1990 cell proliferation (Fig. 5G–J). As well, when PANC-1 and SW1990 cells were treated with 4 Gy X-rays, changes in the number of histone H2AX foci were observed within 0.5 to 48 h. We found the number of γ-H2AX foci peaked at 2 h and gradually declined when irradiated alone (Fig. 5K). However, co-treatment with CCCP and ionizing radiation led to a sustained increase in the number of γ-H2AX foci (Fig. 5L), and when combined with Midiv-1, the number of γ-H2AX foci reached its peak within 2 h (Fig. 5M), which indicate no significance when irradiation alone or co-treated with Mdivi-1 inhibitors. These findings suggest that Parkin/BNIP3-mediated mitophagy can alleviate DNA damage and that the process is irreversible.

### Mitophagy enhances the killing effect of ionizing radiation in vivo

**Fig. 6:**
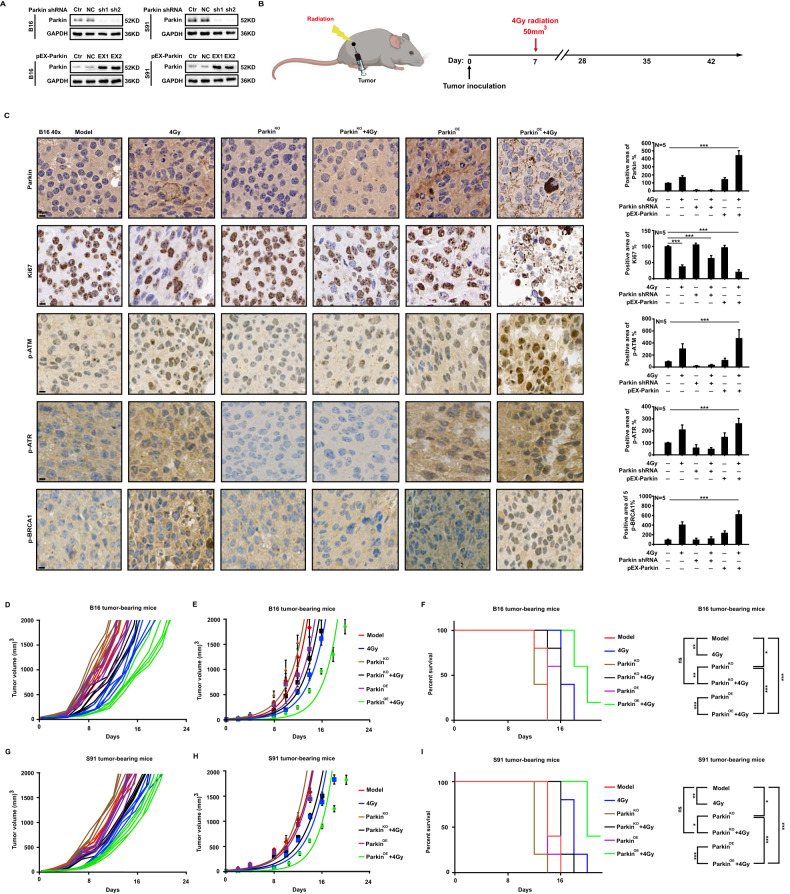
Mitophagy enhances the killing effect of ionizing radiation in vivo. C57BL/6 mice were implanted subcutaneously with wild-type, Parkin^KO^, and Parkin^OE^ B16 or S91 cells (**A**). When the tumor reached about 50 mm^3^, it was irradiated with 4 Gy X-rays (**B**). The expression of Ki67 and phosphorylated ATR, ATM, and BRCA1 was examined using immunohistochemical staining (**C**). Tumor growth was monitored every two days, and the survival of mice was compared under various treatments (**D**–**I**).

To explore how mitophagy affects DNA damage caused by ionizing radiation in vivo, we established Parkin knockout (Parkin^KO^) and overexpression (Parkin^OE^) cell lines of mouse melanoma B16 or S91 cells (Fig. 6A). C57BL/6 mice were subcutaneously inoculated with 5×10^5^ B16 wild-type, B16 Parkin^KO^, B16 Parkin^OE^, and S91 wild-type, S91 Parkin^KO^, or S91 Parkin^OE^ cells. When the tumor reached 50 mm^3^, it was irradiated with 4 Gy X-rays (Fig. 6B). The tumors were harvested 48 h after irradiation and subjected to immunohistochemical staining. The ionizing radiation decreased the expression of malignant proliferation marker Ki67 in B16 Parkin^KO^ tumors compared to wild-type tumors, whereas that in B16 Parkin^OE^ tumors significantly increased. Next, we examined the levels of p-ATM, p-ATR, and p-BRCA1, which are related to DNA damage, and found that radiation significantly decreased the phosphorylation of these proteins in B16 Parkin^KO^ tumors but remarkably increased in B16 Parkin^OE^ tumors (Fig. 6C). Furthermore, X-ray irradiation significantly increased the survival rate of mice in the Parkin^OE^ tumor group compared to that of mice in the Parkin^KO^ tumor group (Fig. 6D–I). Based on these experimental findings, it is possible to conclude that mitophagy can promote DNA damage while enhancing the therapeutic effect of X-rays. Elevating background mitophagy levels will significantly improve tumor radiosensitivity and enhance the effects of radiotherapy.

## Discussion and conclusion

**Fig. 7:**
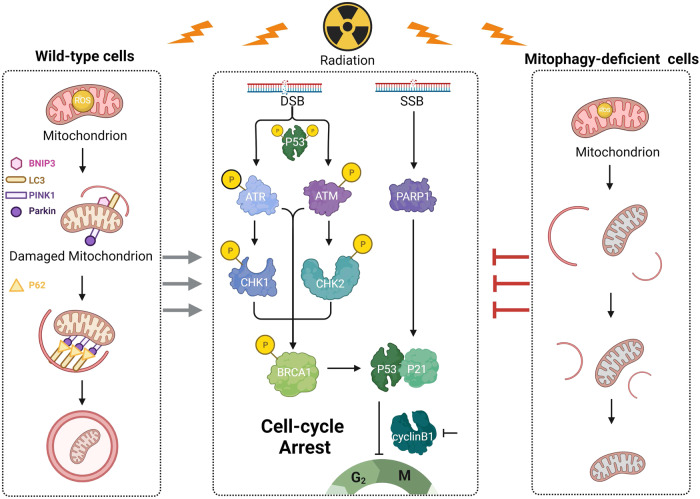
Schematic of the proposed mechanism. Ionizing radiation stimulates Parkin/BNIP3-mediated mitophagy, which increases DNA damage in cancer cells.

Most radiobiology studies have focused on the mechanism of DNA damage to identify new molecular targets for improving radiosensitivity. However, the lack of effective radiosensitizers makes it necessary to continue looking for DDR targets. Tumor radiosensitivity is influenced by various factors, including the capacity of tumors to repair DNA damage and the engagement of multiple mechanisms such as autophagy. Autophagy determines cell fate as an adaptive regulatory mechanism by providing energy for DDR and maintaining intracellular homeostasis [23]. Mitochondria, the “power station,” continuously supply energy to the cell. When stressed, they enter a self-repair mode and prioritize energy supply. However, little is known about mitophagy and DDR. Therefore, we investigated the relationship between mitophagy and DNA damage. Numerous studies have demonstrated that ionizing radiation is capable of causing cell cycle arrests, which allow time for DNA damage to be repaired. We discovered that an increase in the X-ray dose increased the G2/M phase block rate and levels of DNA damage sensors γ-H2AX, 53BP1, and PARP1, which then triggered the DDR process of the ATR/Chk1 and ATM/Chk2 signaling pathways. These findings are in line with the results of several previous studies [24–27], ionizing radiation induces mitochondrial morphology and function changes by increasing the expression of Parkin and BNIP3 and guiding damaged mitochondria to lysosome degradation pathways, which is known as mitophagy. The relationship between mitophagy and DDR is not extensively studied, but some evidence points to the effect of autophagy on DDR. During DDR, autophagy can be used as a source of energy for preserving cell cycle arrest and facilitating DNA repair. Muñoz-Gámez et al. [28] suggests that autophagy-deficient cells are more sensitive to DNA damage and that autophagy can help them survive. A study showed that ATM localizes to mitochondria and regulates PINK1-Parkin-mediated mitophagy, suggesting that DNA damage contributes to mitophagy [29]. However, our study found that CCCP induced mitophagy while also causing minor DNA damage. X-ray irradiation combined with CCCP induced more damage sites than X-ray irradiation alone. We believe that mitophagy is upstream of DNA damage because no effect was seen when DNA damage repair inhibitors were used. Galati et al. [23] claim that an increase in ROS in dysfunctional mitochondria causes DNA damage and PARP1 activation. Ionizing radiation can also cause extensive ROS production in damaged mitochondria, which supports our conclusion from this perspective. In animal experiments, we discovered that increasing the background mitophagy level in melanoma cells followed by X-ray irradiation could result in more DNA damage than that in mitophagy-deficiency tumors, slowing the speed of tumor growth and extending the survival time of tumor-bearing mice. There was no significant difference between the growth rates of the wild-type tumor and the mitophagy-deficient tumor after X-ray irradiation. This finding aligns with our examination of patient samples from the TCGA database, where individuals with elevated levels of mitophagy in their tumors demonstrated improved survival rates. Therefore, we conclude that ionizing radiation can cause Parkin/BNIP3-mediated mitophagy, enhance DNA damage, and improve the radiosensitivity of tumor cells, eventually promoting tumor cell death (Fig. 7). Our findings could lead to a new strategy for clinical radiotherapy; combining radiation with a mitophagy inducer may achieve better therapeutic effects. Although our study suggests that Parkin/BNIP3-mediated mitophagy can increase ionizing radiation-induced DNA damage, many molecular mechanisms remain unknown. Sarkar et al. claimed that ATM-Parkin interaction is important for Parkin stability [30]. Gu et al. reported that the induction of mitophagy by Lead (Pb) is mediated by the activation of the PINK1/Parkin pathway through ATM [31]. Xi et al. reported that phosphorylation of BNIP3 by oxidized ATM resulted in the accumulation of autophagosomes and the release of exosomes in hypoxic breast cancer-associated fibroblasts (CAFs) [32]. Hence, further research is required to determine whether Parkin and BNIP3 interact with ATR and ATM or whether they are linked by cytokines or chemokines in the case of ionizing radiation.

## Materials and methods

### Cell culture

Human pancreatic cancer cell lines PANC-1 and SW1990, along with murine melanoma cell lines B16 and S91, were acquired from the Cell Bank of the Chinese Academy of Sciences. All cell lines were cultured in Dulbecco’s modified Eagle’s medium supplemented with 10% fetal bovine serum (FBS), 100 U/mL penicillin, and 100 μg/mL streptomycin. PANC-1 and SW1990 cell lines were used to generate Parkin and BNIP3 knockdown subclones using Parkin siRNA(h) and BNIP-3 siRNA(h), respectively; B16 and S91 cell lines were used to generate Parkin and BNIP3 knockout subclones using Parkin shRNA(m) and BNIP3 shRNA(m), respectively. In 6-well plates, four types of cells were cultured at a density of 1 × 10^5^ cells per well for a duration of 24 h. Following that, transfection was carried out for an additional 24 h using si-Control, si-Parkin, and si-BNIP3; sh-Control, sh-Parkin, and sh-BNIP3, along with Lipofectamine RNAiMAX reagent, following the instructions provided by the manufacturer (Invitrogen, Carlsbad, CA, USA). All cell cultures were maintained at 37 °C in a humidified incubator with 5% CO_2_ and were verified to be free from microbial contamination. Supplementary table 1 lists related reagents and antibodies. Supplementary table 2 lists related plasmids and sequence.

### Experimental mouse model

The Lanzhou Veterinary Research Institute provided 6–8-week-old male C57BL/6 mice. Animals were kept at the First Hospital of Lanzhou University under pathogen-free conditions, with a 12 h light-dark cycle and a temperature-controlled environment. There was no restriction on the mice’s access to food and water. The Ethical Committee of the First Hospital of Lanzhou University provided approval for all animal experiments, and the guidelines outlined in EU Directive 2010/63/EU were followed. 5 × 10^5^ cells were subcutaneously injected into C57BL/6 mice to induce B16 and S91 tumors. Upon reaching a size of ~50 mm^3^, the tumors were subjected to local irradiation using 4 Gy X-rays. The mice carrying the tumors were closely observed, and regular documentation of tumor growth was performed. The above method refers to our previous study [33].

### Radiotherapy

In this experiment, x-rays were generated using an X-Rad 225 generator (Precision, Minneapolis, MN, USA) (energy: 225 kV/13.3 mA). Pancreatic cancer cells and melanoma cells were exposed to radiation in cell culture dishes. Anesthesia was administered to mice bearing melanoma before they were placed on the platform (pentobarbital sodium 50 mg/kg, intraperitoneally). The radiation was targeted using a special lead protection device, allowing it only to reach the tumor site.

### PAGE and immunoblotting

A protease inhibitor cocktail and 150 mM sodium chloride were added to RIPA buffer, along with 1.0% NP-40, 0.5% sodium deoxycholate, and 0.1% SDS. After undergoing ultrasonication, the cell lysate was combined with 5x loading buffer. After incubation on ice for 30 min, the mixture was placed in a metal bath at 98 °C for 10 min to denaturate the proteins. Protein concentration and separation were accomplished by applying 80 V and 120 V voltages to NuPAGE® Novex® 4–12% Bis-Tris Protein Gels. Using an electrotransfer voltage of 120 V and an electric current of 0.2 A, the proteins were transferred onto PVDF membranes. Protein molecular weight was used to determine the transfer duration. Following this, the membranes were subjected to blocking using 5% bovine serum albumin in 1×TBS containing 0.1% Tween^®^-20 (1×TBST) for 1 h at a temperature of 24 °C. The membranes were then treated with the appropriate primary antibodies overnight at 4°C. After washing, the membranes were incubated for 2 h with HRP-conjugated secondary antibodies at 24 °C. Using the ImageQuant LAS 4000 system (GE Healthcare), we detected peroxidase activity using the ECL Western Blotting Detection Reagent (Merck-Millipore).

### Flow cytometry

Trypsin digestion was followed by the collection of the cells in a medium that contained 5% FBS. Single-cell suspensions were created by gently teasing cells through 70-μm sieves into cold PBS. A centrifuge was then applied for 10 min at 800 rpm to the cells. We discarded the supernatant and re-suspended the cells in staining buffer, which contained either 2 μM dihydrorhodamine 123 (DHR123) for the detection of mitochondrial reactive oxygen species (ROS) or 15 μg/ml acridine orange for the assessment of intracellular acidity. After incubating the cells in the absence of light for 10 min at room temperature, flow cytometry was performed. For the cell cycle arrest assay, a total of 1 × 10^6^ cells were collected. The supernatant was removed, and 1 ml of PBS was added to the cells at room temperature. The cells were slowly added to 3 ml of cold anhydrous ethanol (−20 °C), shaken vigorously while adding, and fixed overnight at −20 °C. Following a fixation period of 24 h, the cells underwent centrifugation at 200 × *g* for a duration of 5 min. To resuspend the cells, 1 ml of cold PBS was added. After centrifuging again, the supernatant was aspirated, 100 ml PBS was added, then propidium iodide (20 μg/mL) was added and swirled for 10 s. The solution was thoroughly mixed and an incubated at room temperature for a duration of 30 min, ensuring it was kept away from light. Subsequently, flow cytometry analysis was performed using a Merck-Millipore FlowSight flow cytometer.

### Immunofluorescence

#### Fixed image collection

Cells were treated with 4% paraformaldehyde solution and fixed for a duration of 10 min. Then removed the liquid supernatant and incubated the cells for 20 min with chilled methanol. Afterward, the cells were washed with 75% ethanol containing glacial acetic acid. In order to permeate the cell membranes, they were rehydrated and then treated with 0.5% Triton X-100 solution for five min. The samples were then subjected to blocking with a mixture of goat serum and PBS (diluted at a ratio of 1:20) for 2 h. An incubation of two h was followed by the addition of the primary antibody (diluted at a ratio of 1:500). After undergoing five rinses with PBST, the appropriate fluorochrome-conjugated secondary antibody (diluted at a ratio of 1:1000) was introduced, and the cells were then subjected to incubation in a dark environment for a duration of 1.5 h. Subsequently, 10 μL of 4′, 6-diamidino-2-phenylindole (DAPI) was introduced, and images were captured using a fluorescence microscope.

#### Live-image collection

The cells were incubated in a dark environment for a duration of 30 min in a medium containing a mixture of Lyso-Tracker Red (60 nM) and Mito-Tracker™ Green (150 μm). A 10-min incubation at room temperature was followed by the addition of Hoechst 33342 (1 μm). Subsequently, images were obtained using fluorescence microscopy. The above method refers to our previous study [34].

### Immunohistochemical analysis

The tumor specimen was embedded in paraffin, and subsequently, it was sectioned into slices measuring 3 μm in thickness. These slices were then placed on slides with a positive charge. Using a 1:200 dilution ratio, the primary antibody was applied for immunostaining. The Lab Vision™ UltraVision™ Quanto Detection System detection kit was utilized in conjunction with 3,3′-diaminobenzidine tetrahydrochloride as the chromogen. Moreover, the paraffin-embedded tumors underwent staining with hematoxylin and eosin, following standard protocols. In each sample, a total of five fields were counted for every individual slide. ImageJ software, available at http://imagej.nih.gov/ij/, was used to determine the percentage of cells displaying phenotypic alterations.

### Comet assay

Cells were collected 48 h post-exposure to a radiation dose of 4 Gy X-ray to generate a single-cell suspension. Application of the cell suspension to prepared slides was conducted at 37 °C using low melting point agarose (LM agarose). In the following step, the slides were immersed in a lysate solution and kept at 4 °C overnight before being electrophoresed. After the LM agarose had completely dried, the slides were neutralized and subjected to staining using a fluorescent dye. The resulting DNA damage was visualized using a fluorescence microscope.

### Statistical analysis

Unless otherwise noted, all tests were carried out in triplicate and at least twice. The data was analyzed and graphs were generated using Origin 9.0 software. The Student’s *t* test was used to determine statistical significance, one-way ANOVA with post hoc Dunnett test, and Kaplan–Meier analysis with log-rank test. Statistical significance was denoted as *p < 0.05, **p < 0.01, and ***p < 0.001.

## Supplementary information

Supplementary Table 1

Supplementary Table 2

Original Data File

## Data Availability

All data generated and analyzed during this study are included in this article. Each experiment was performed at least three times independently.
