# Supplementary material for: Ionizing radiation triggers mitophagy to enhance DNA damage in cancer cells
Source: Cell Death Discov. 2023 Jul 28;9:267. doi: 10.1038/s41420-023-01573-0 (PMC10382586; doi:10.1038/s41420-023-01573-0)
Supplement: Supplementary file 1 — Supplementary Table 1 [file 41420_2023_1573_MOESM1_ESM.docx]

| **Table1**. Reagents and resources | | |
| --- | --- | --- |
| Reagent Source Identifier | | |
| **Chemicals**  AZ9482  Gimeracil  CCCP  VPA  **Fluorescent dye**  Mito-Tracker™ Green  Lyso-Tracker Red  Dihydrorhodamine 123  Acridine orange  Propidium iodide  Hoechst 33342  **Plasmid**  Parkin(h)  Parkin(m)  BNIP3(h)  **Antibodies**  Parkin  LC3B  p62  Beclin1  LAMP1  LAMP2  Ki67  c-Myc  Ki67  Malondialdehyde  BNIP3  53BP1  γ-H2AX  PARP1  p-BRCA1  BRCA1  p-ATM  ATM  p-ATR  ATR  p53  p21  Cyclin B1  p-CHK1  CHK1  p-CHK2  CHK2 | MedChemExpress  MedChemExpress  MedChemExpress  MedChemExpress  Invitrogen  Beyotime  Solarbio  Invitrogen  Solarbio  Invitrogen  santa cruz  santa cruz  santa cruz  Proteintech  Abcam  Abcam  Abcam  Abcam  Abcam  Abcam  Abcam  Servicebio  Abcam  Proteintech  santa cruz  Abcam  Proteintech  Affinit  Santa cruz  Abcam  Abcam  Affinit  Abcam  Abcam  Abcam  Abcam  Cell Signaling Technology  Cell Signaling Technology  Cell Signaling Technology  Cell Signaling Technology | 1825345-33-2  103766-25-2  555-60-2  99-66-1  M7514  C1046  S1100  A1301  P8080  H3570  sc-42158  sc-42159  sc-37451  66674-1-Ig  ab192890  ab109012  ab207612  ab289548  ab199946  ab16667  ab32072  gb111141  ab27615  68091-1-Ig  sc515841  ab26350  13371-1-AP  AF3289  sc-6954  ab81292  ab32420  DF7512  ab2905  ab179477  ab109520  ab32053  12302  2360  2197  6334 |
