# Supplementary material for: Ionizing radiation triggers mitophagy to enhance DNA damage in cancer cells
Source: Cell Death Discov. 2023 Jul 28;9:267. doi: 10.1038/s41420-023-01573-0 (PMC10382586; doi:10.1038/s41420-023-01573-0)
Supplement: Supplementary file 2 — Supplementary Table 2 [file 41420_2023_1573_MOESM2_ESM.docx]

| **Table2**. List of plasmids and sequence | |
| --- | --- |
| Plasmids name Sequence | |
| **Parkin siRNA(h)**  (sc-42158)  **Parkin shRNA(m)**  (sc-42159)  **BNIP3 siRNA(h)**  (sc-37451) | sc-42158A:  • Sense: 5′-CUACCCAGUGACCAUGAUAtt-3′，  • Antisense: 5′-UAUCAUGGUCACUGGGUAGtt-3′.  sc-42158B:  • Sense: 5′-CUACAGAGUCGAUGAAAGAtt-3′，  • Antisense: 5′-UCUUUCAUCGACUCUGUAGtt-3′.  sc-42158C:  • Sense: 5′-GGAUUGGAUUUCAGUUCAAtt-3′，  • Antisense: 5′-UUGAACUGAAAUCCAAUCCtt-3′.  sc-42159-SHA:  • Sense: GGAACAACAGAGUAUUGUAtt  • Antisense: UACAAUACUCUGUUGUUCCtt  sc-42159-SHB:  • Sense: CGAUGCAUAUAAGCACAUAtt  • Antisense: UAUGUGCUUAUAUGCAUCGtt  sc-42159-SHC:  • Sense: CUUGAGGCCUAGAGCUAAAtt  • Antisense: UUUAGCUCUAGGCCUCAAGtt  sc-37451A:  • Sense: GAACUGCACUUCAGCAAUAtt  • Antisense: UAUUGCUGAAGUGCAGUUCtt  sc-37451B:  • Sense: CCAUAGCAUUGGAGAGAAAtt  • Antisense: UUUCUCUCCAAUGCUAUGGtt  sc-37451C:  • Sense: GAAGGCACCUACUCAGUAUtt  • Antisense: AUACUGAGUAGGUGCCUUCtt |
