# Supplementary material for: Ionizing radiation triggers mitophagy to enhance DNA damage in cancer cells
Source: Cell Death Discov. 2023 Jul 28;9:267. doi: 10.1038/s41420-023-01573-0 (PMC10382586; doi:10.1038/s41420-023-01573-0)
Supplement: Supplementary file 3 — Original Data File [file 41420_2023_1573_MOESM3_ESM.pdf]

Full and uncropped western blot for Figure 1D

Lanes 1-8 are on the figure

PANC-1 SW1990

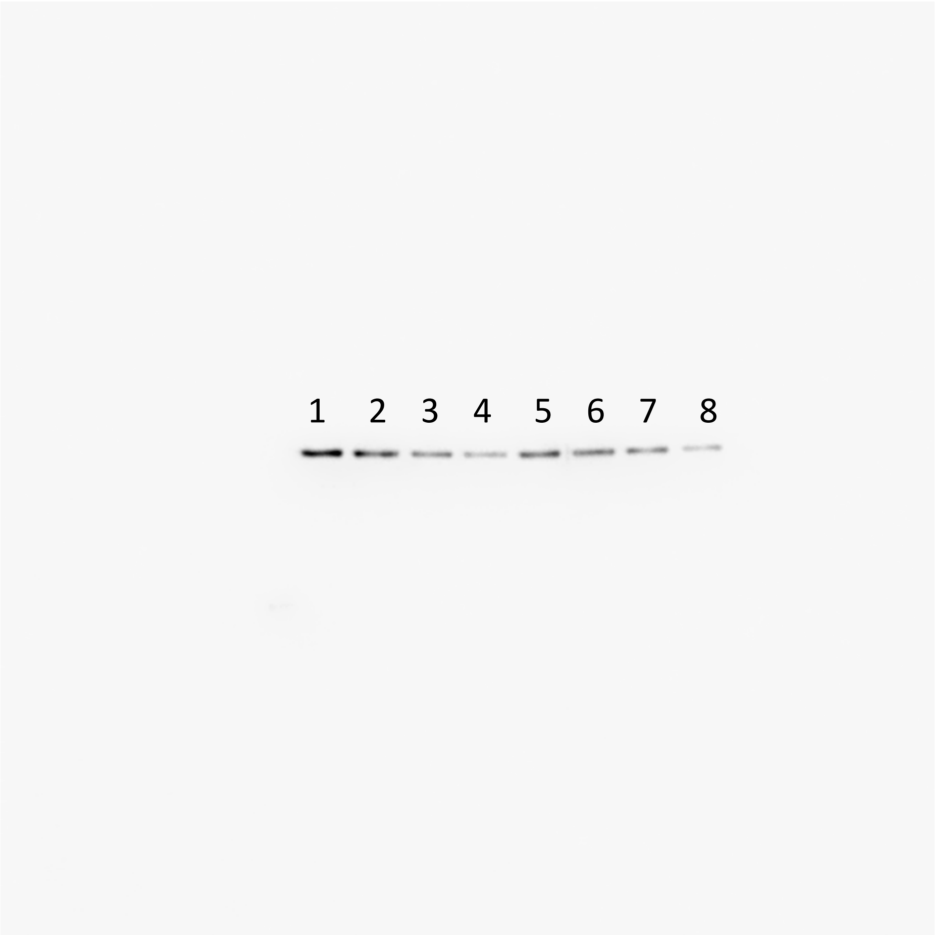

KI67

Full and uncropped western blot for Figure 1D

Lanes 1-8 are on the figure

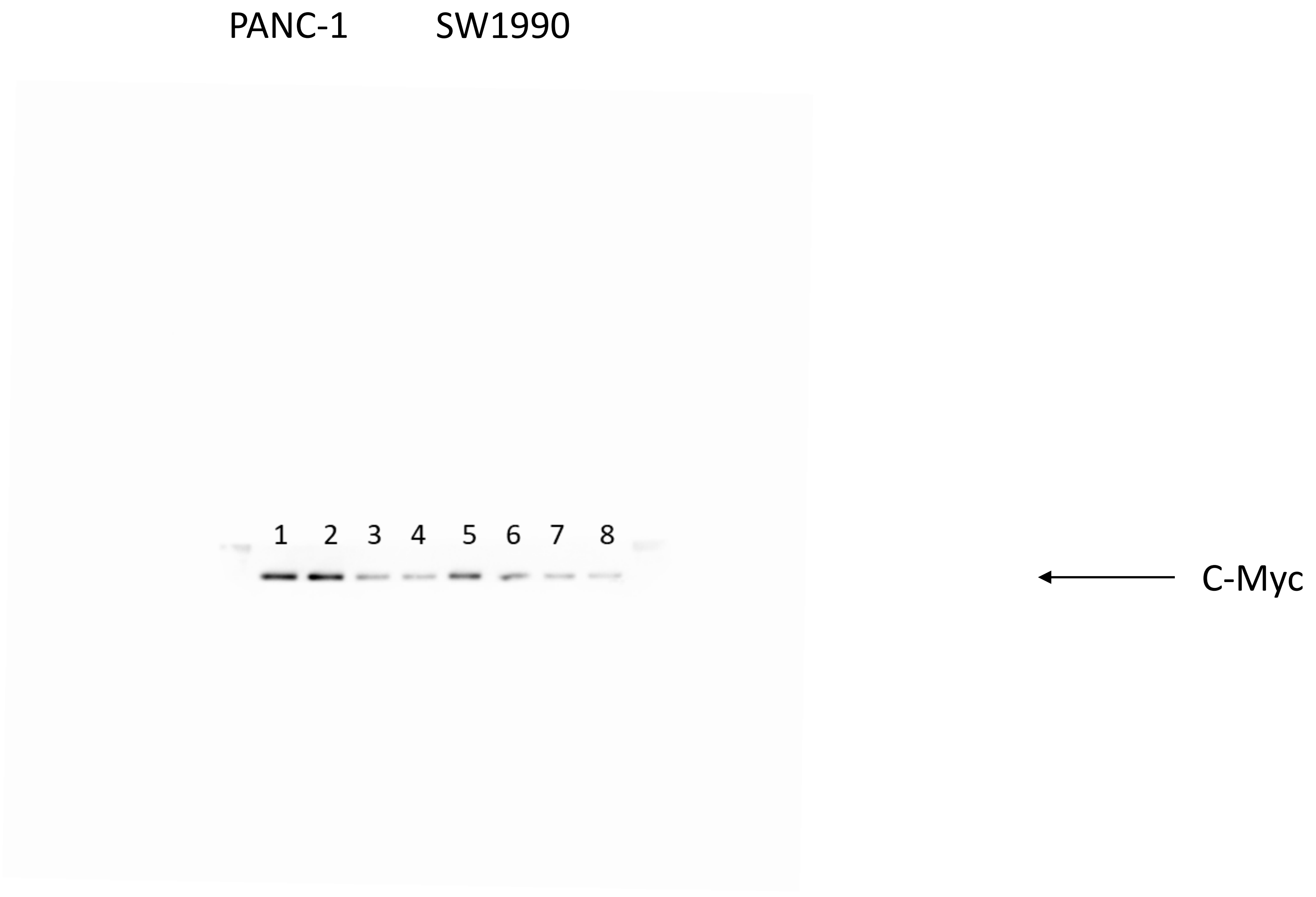

Full and uncropped western blot for Figure 1D

Lanes 1-8 are on the figure

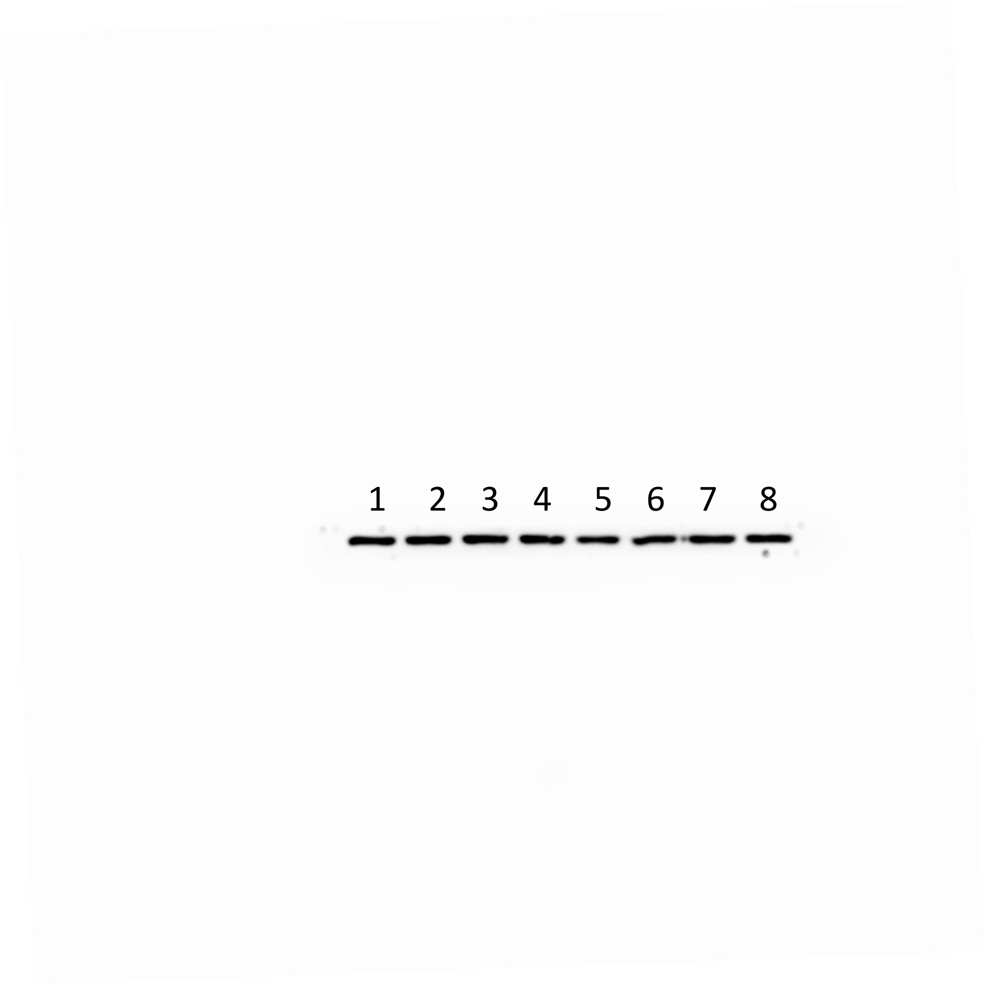

Full and uncropped western blot for Figure 1D

Lanes 1-8 are on the figure

B16 S91

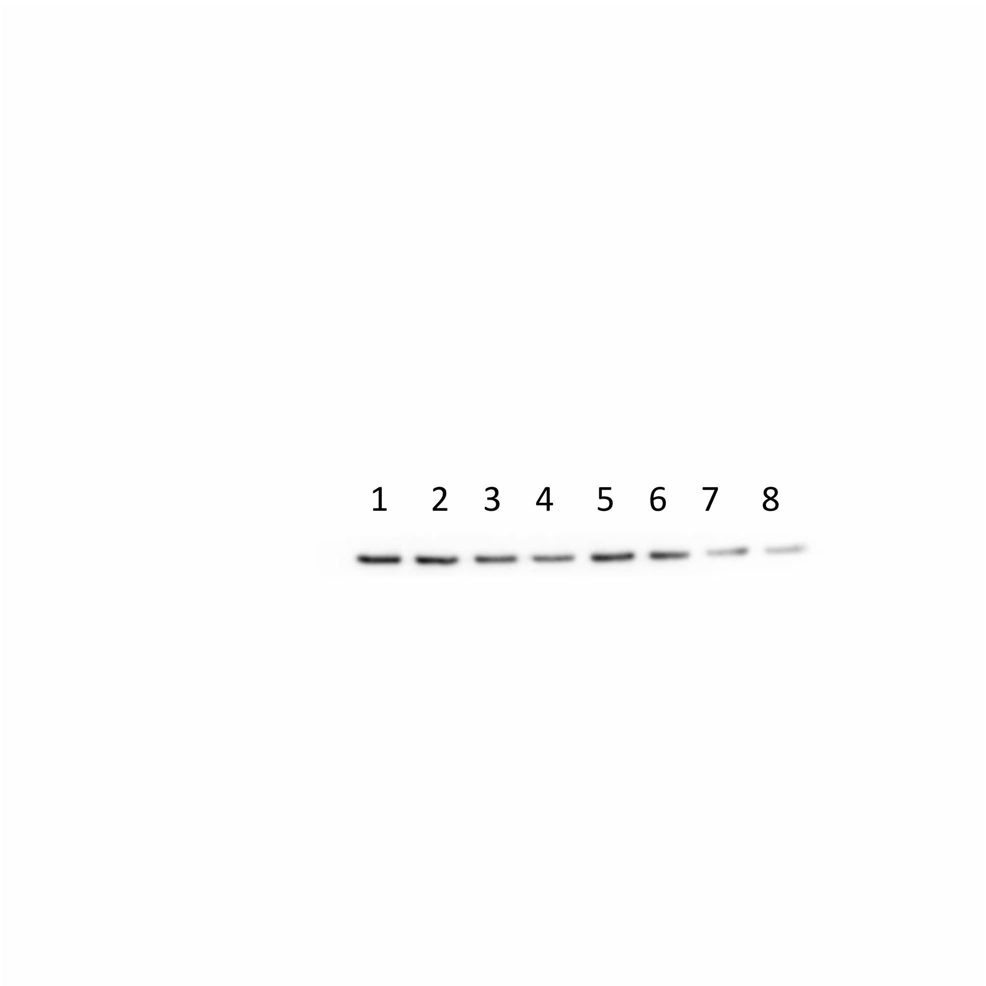

Ki67

Full and uncropped western blot for Figure 1D

Lanes 1-8 are on the figure

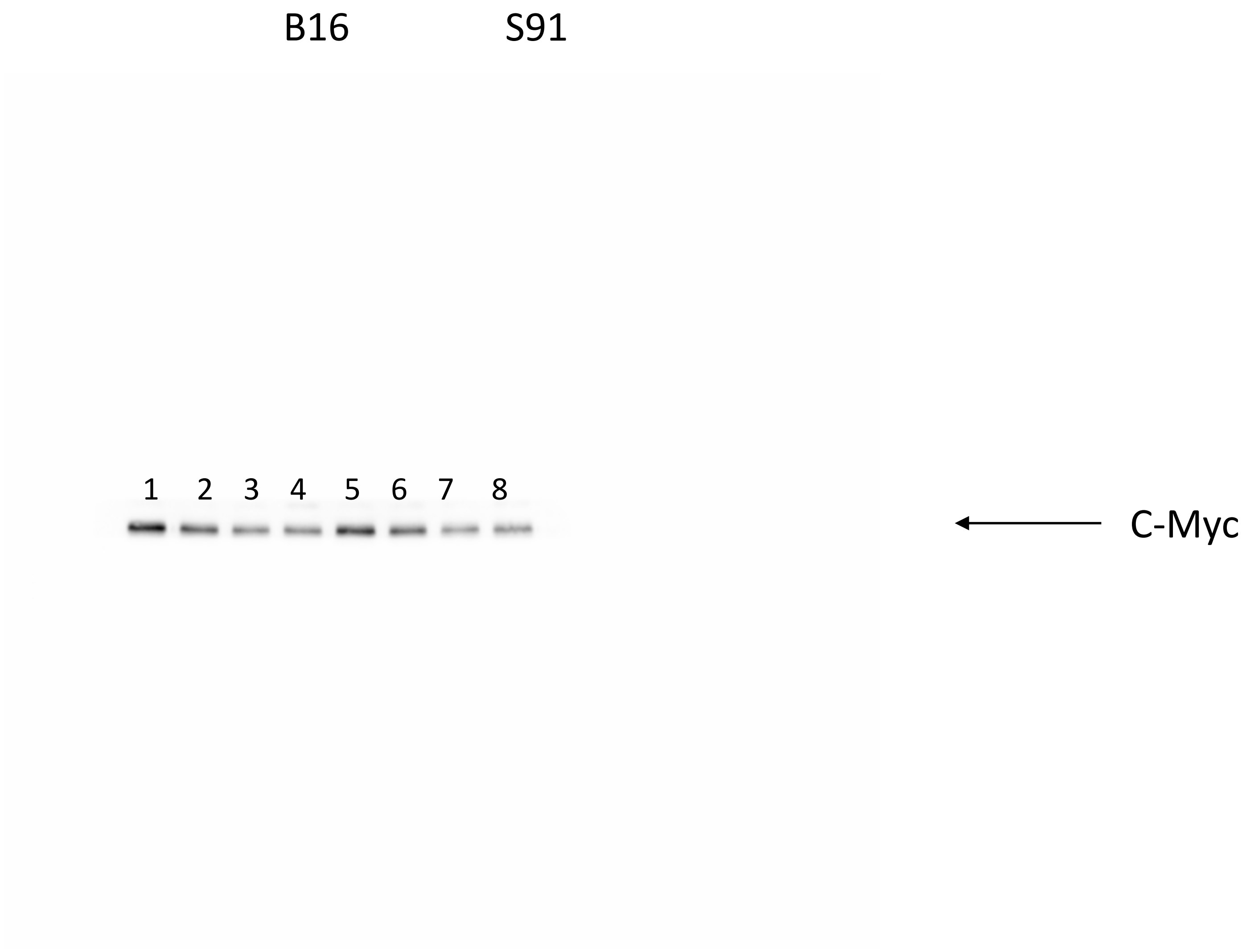

Full and uncropped western blot for Figure 1D

Lanes 1-8 are on the figure

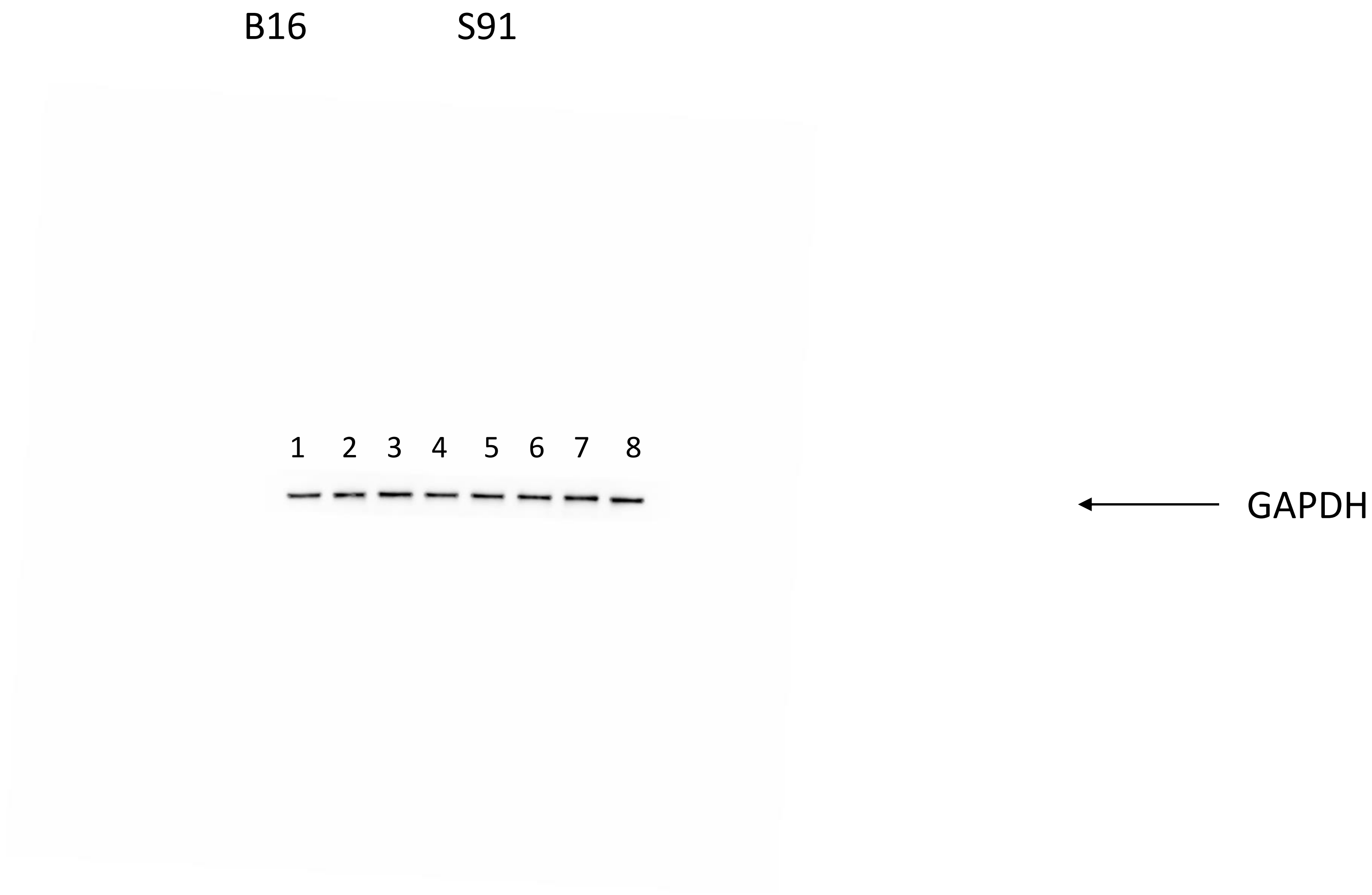

Full and uncropped western blot for Figure 2B

Lanes 1-8 are on the figure

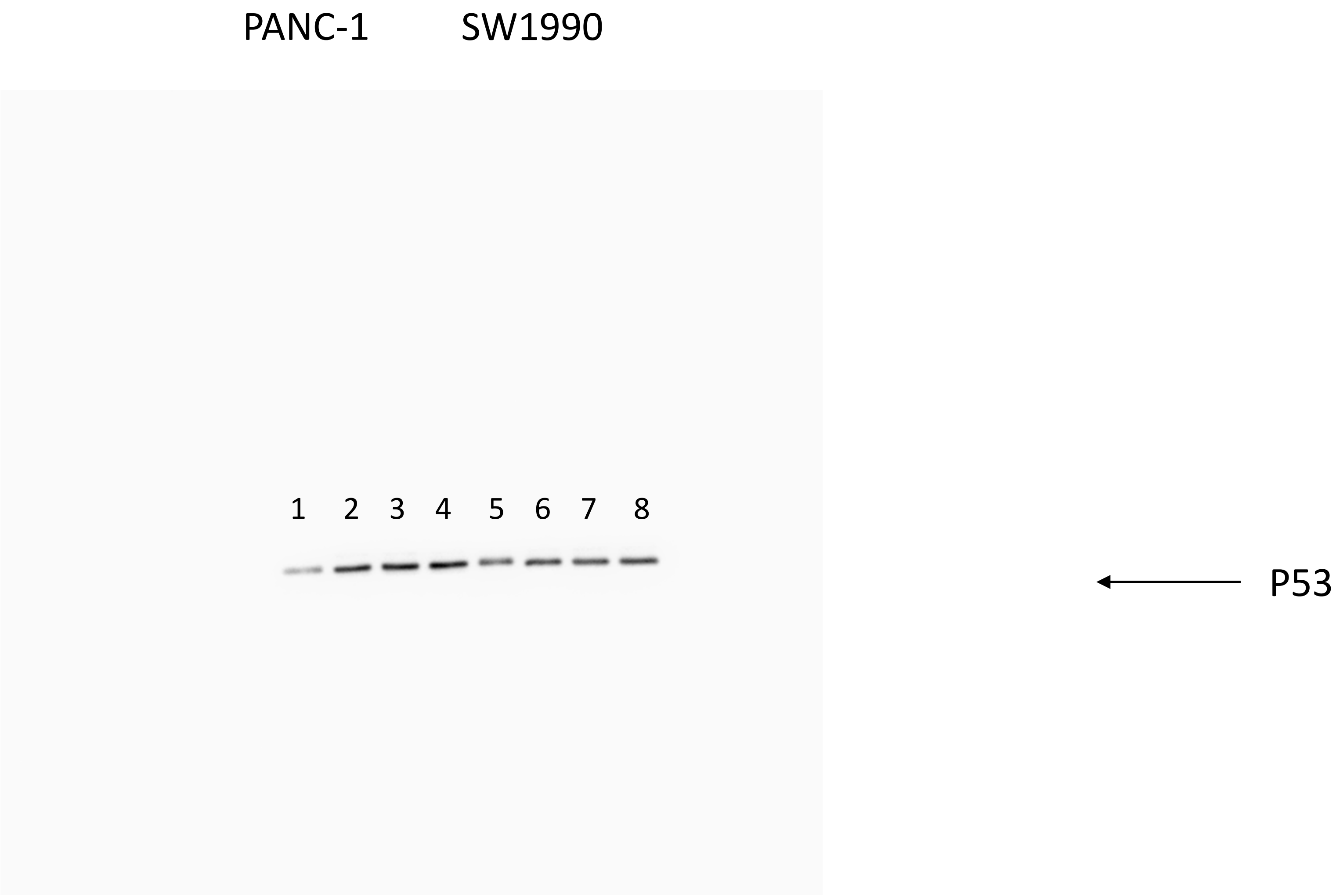

Full and uncropped western blot for Figure 2B

Lanes 1-8 are on the figure

PANC-1 SW1990

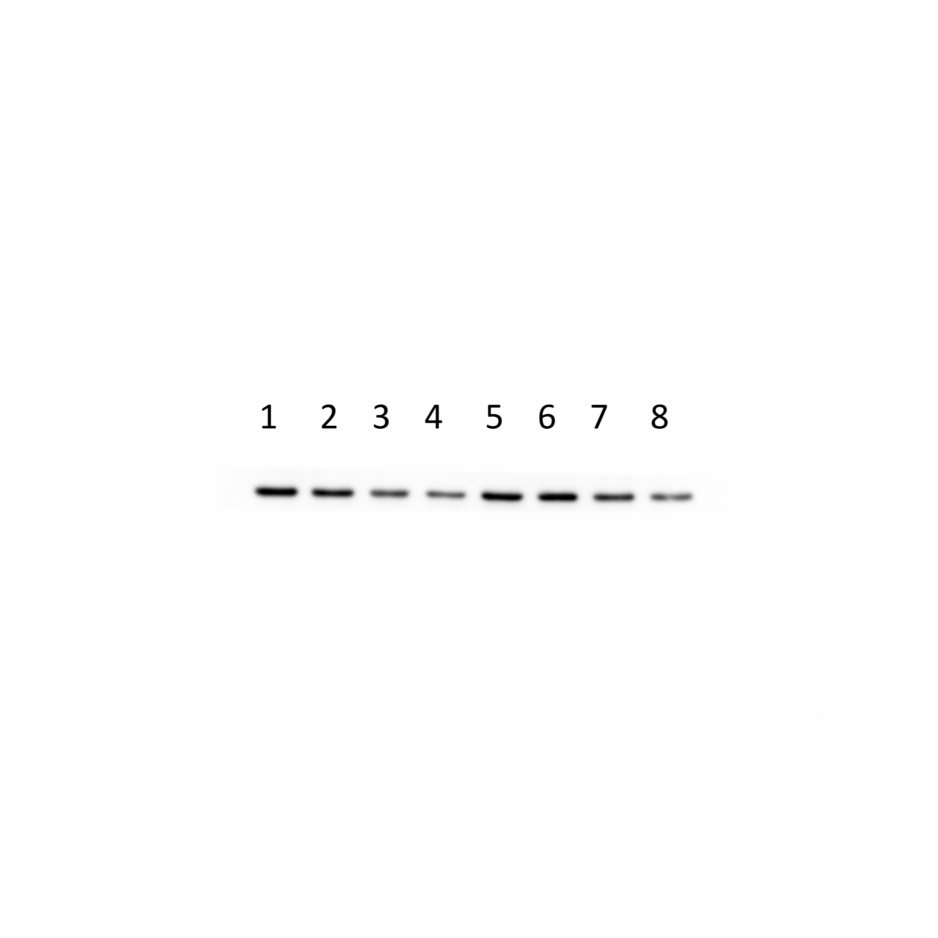

Cyclin B1

Full and uncropped western blot for Figure 2B

Lanes 1-8 are on the figure

PANC-1 SW1990

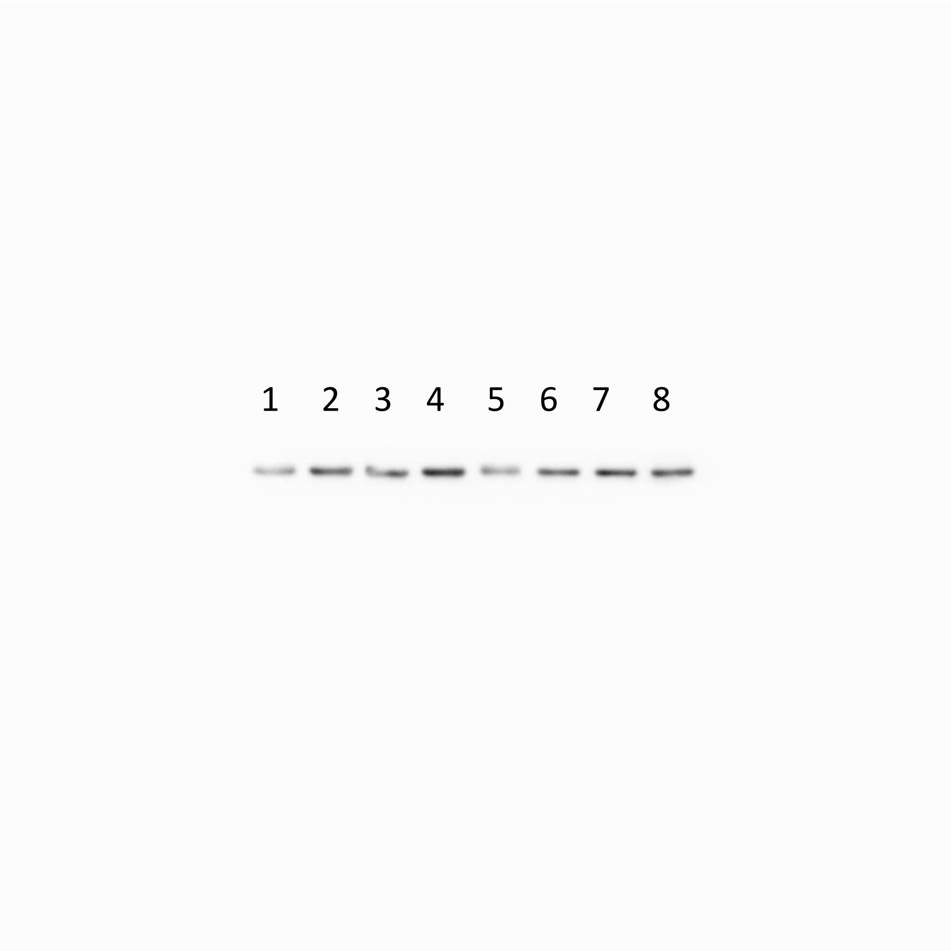

P21

Full and uncropped western blot for Figure 2B

Lanes 1-8 are on the figure

PANC-1 SW1990

1 2 3 4 5 6 7 8

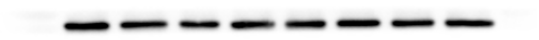

GAPDH

Full and uncropped western blot for Figure 2C

Lanes 1-8 are on the figure

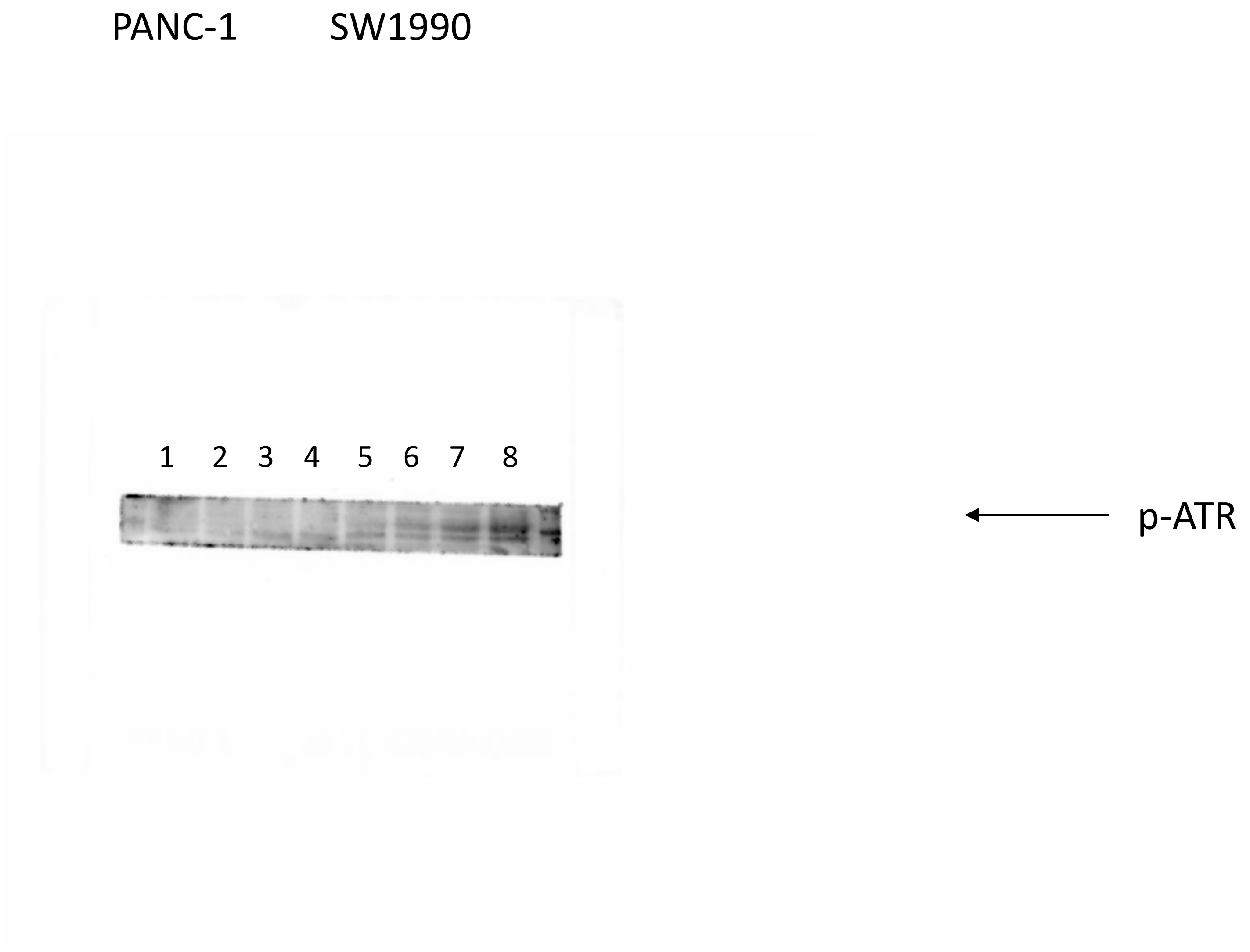

Full and uncropped western blot for Figure 2C

Lanes 1-8 are on the figure

PANC-1 SW1990

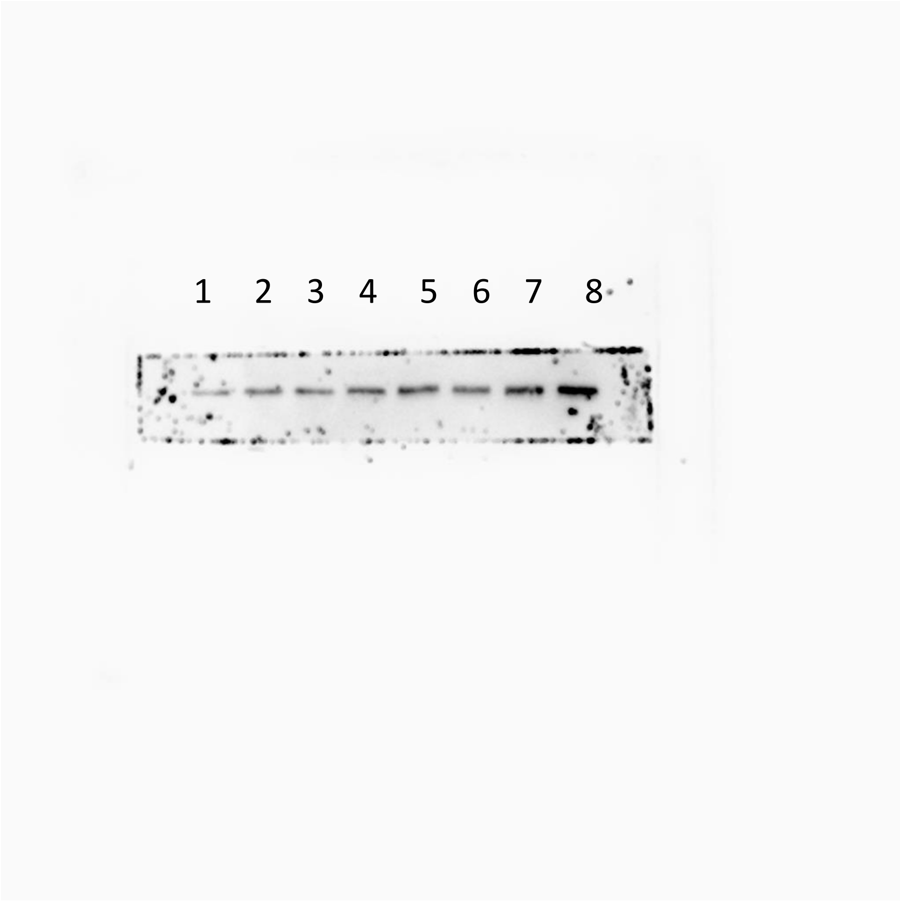

ATR

Full and uncropped western blot for Figure 2C

Lanes 1-8 are on the figure

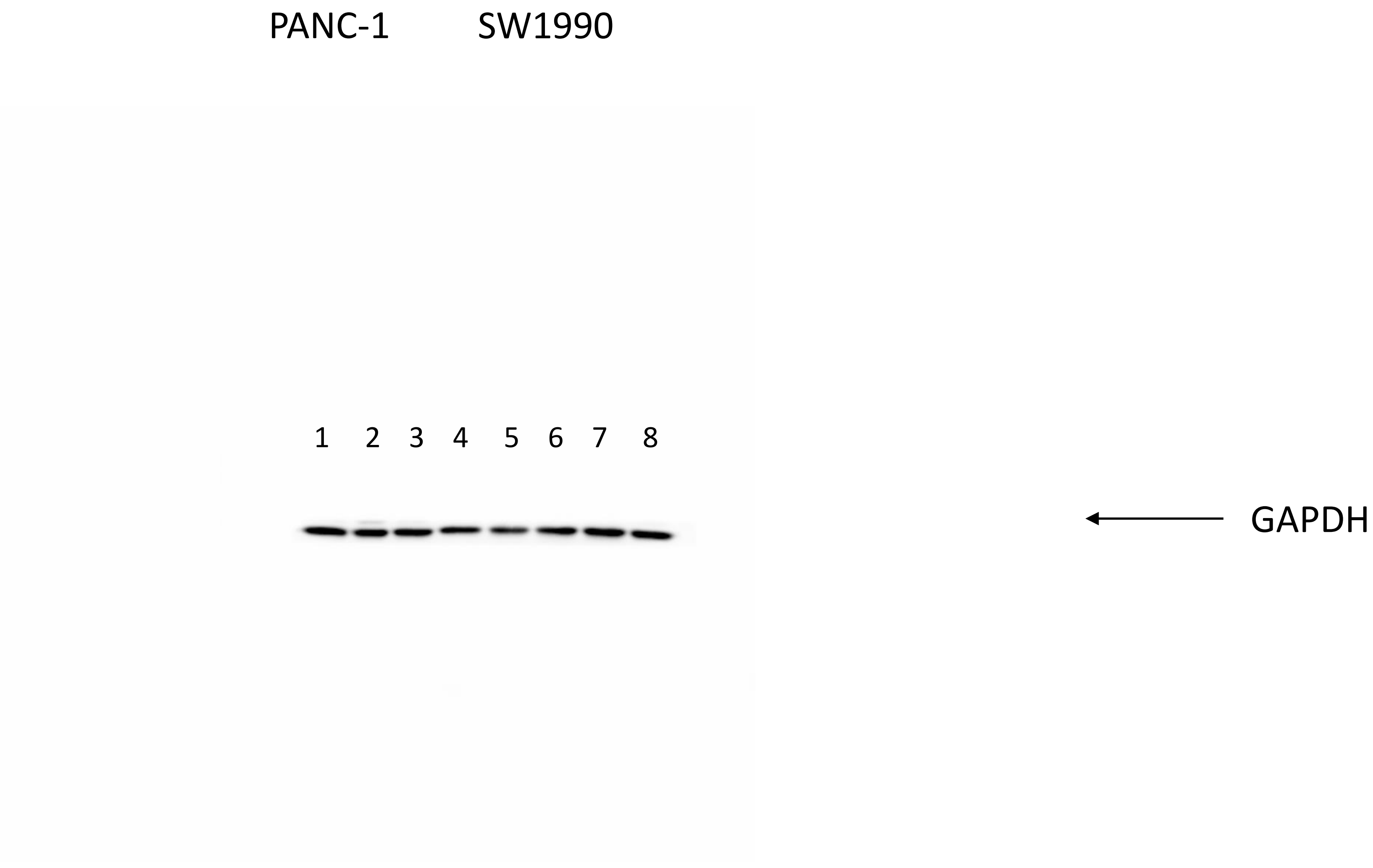

Full and uncropped western blot for Figure 2D

Lanes 1-8 are on the figure

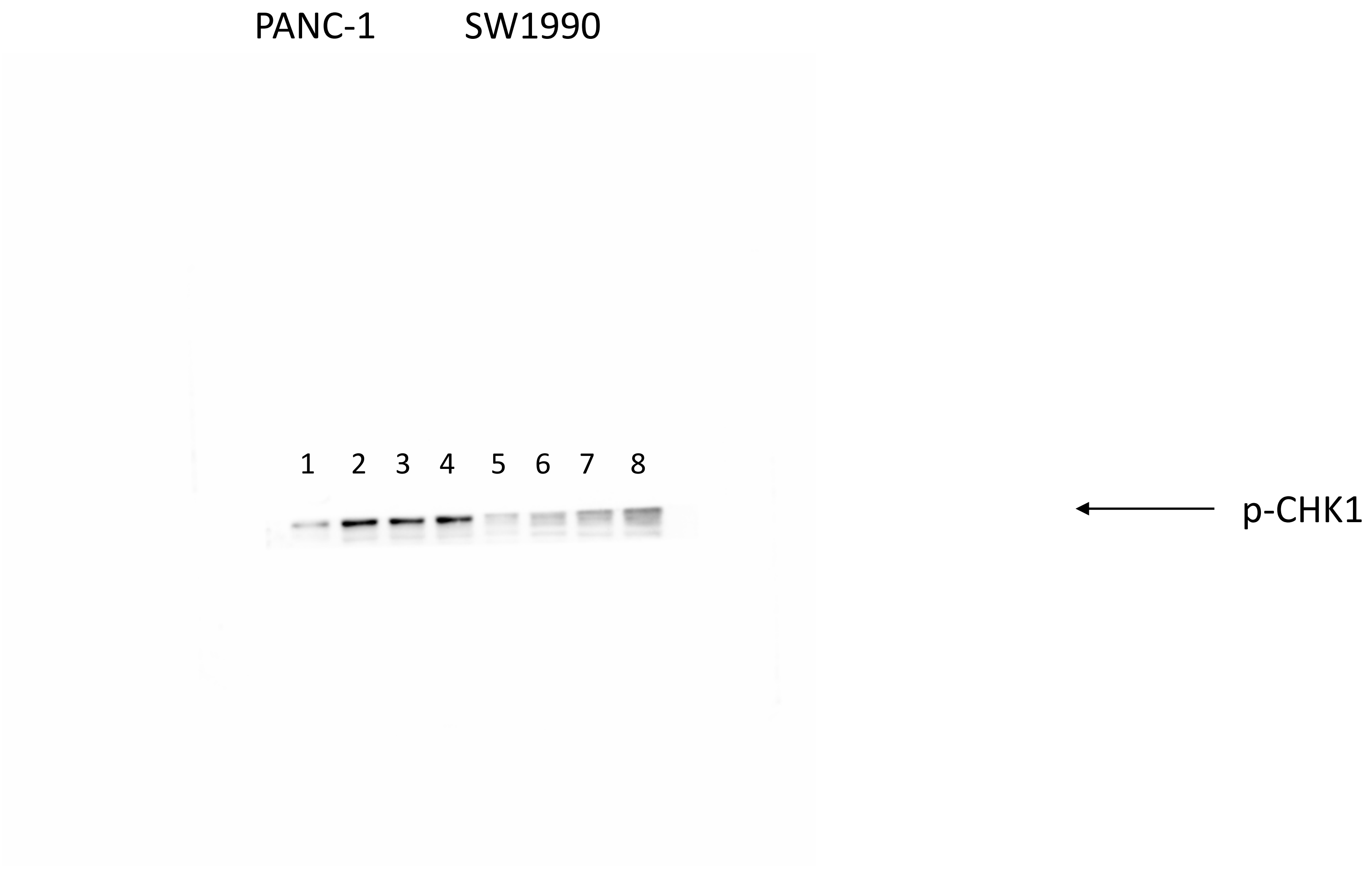

Full and uncropped western blot for Figure 2D

Lanes 1-8 are on the figure

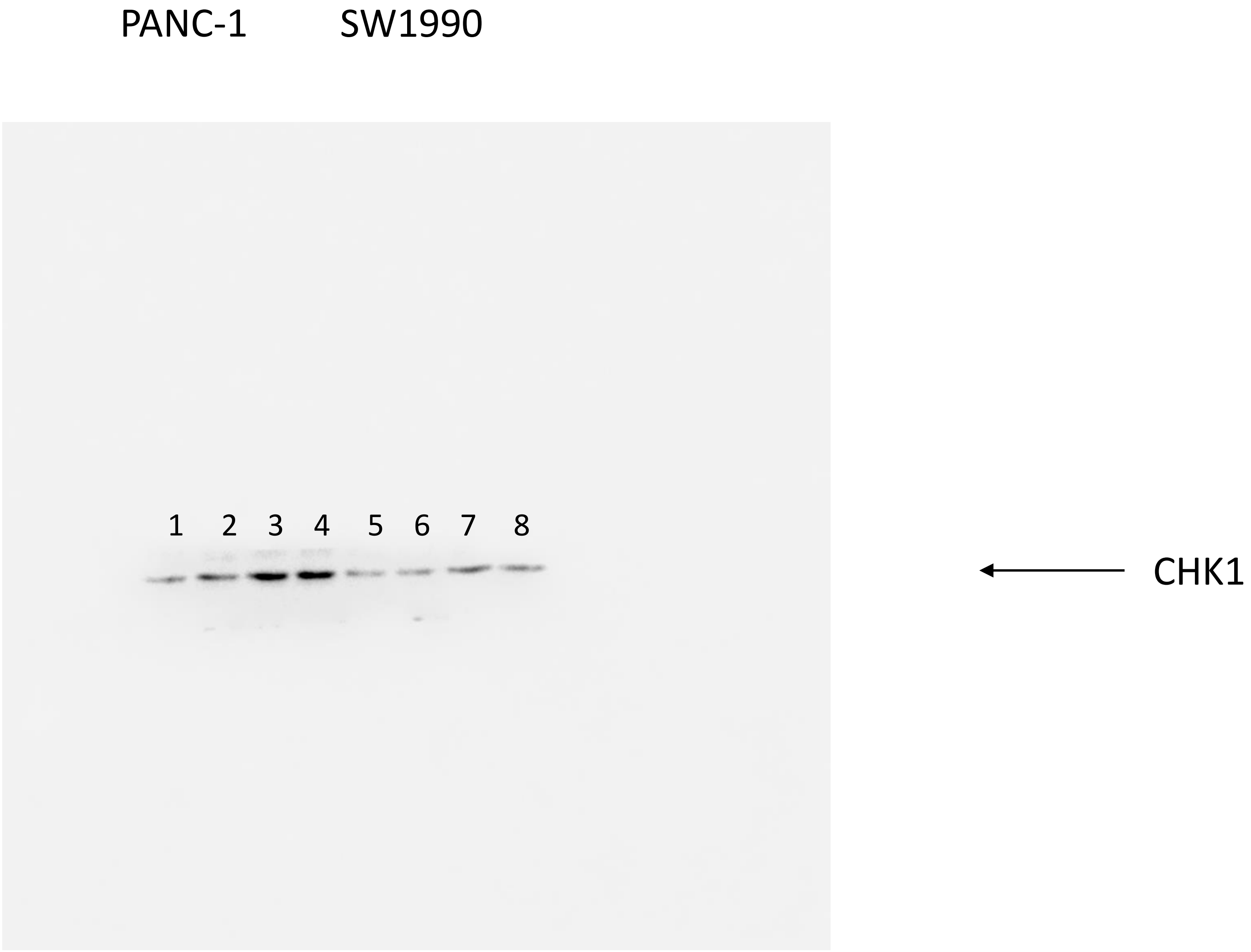

Full and uncropped western blot for Figure 2D

Lanes 1-8 are on the figure

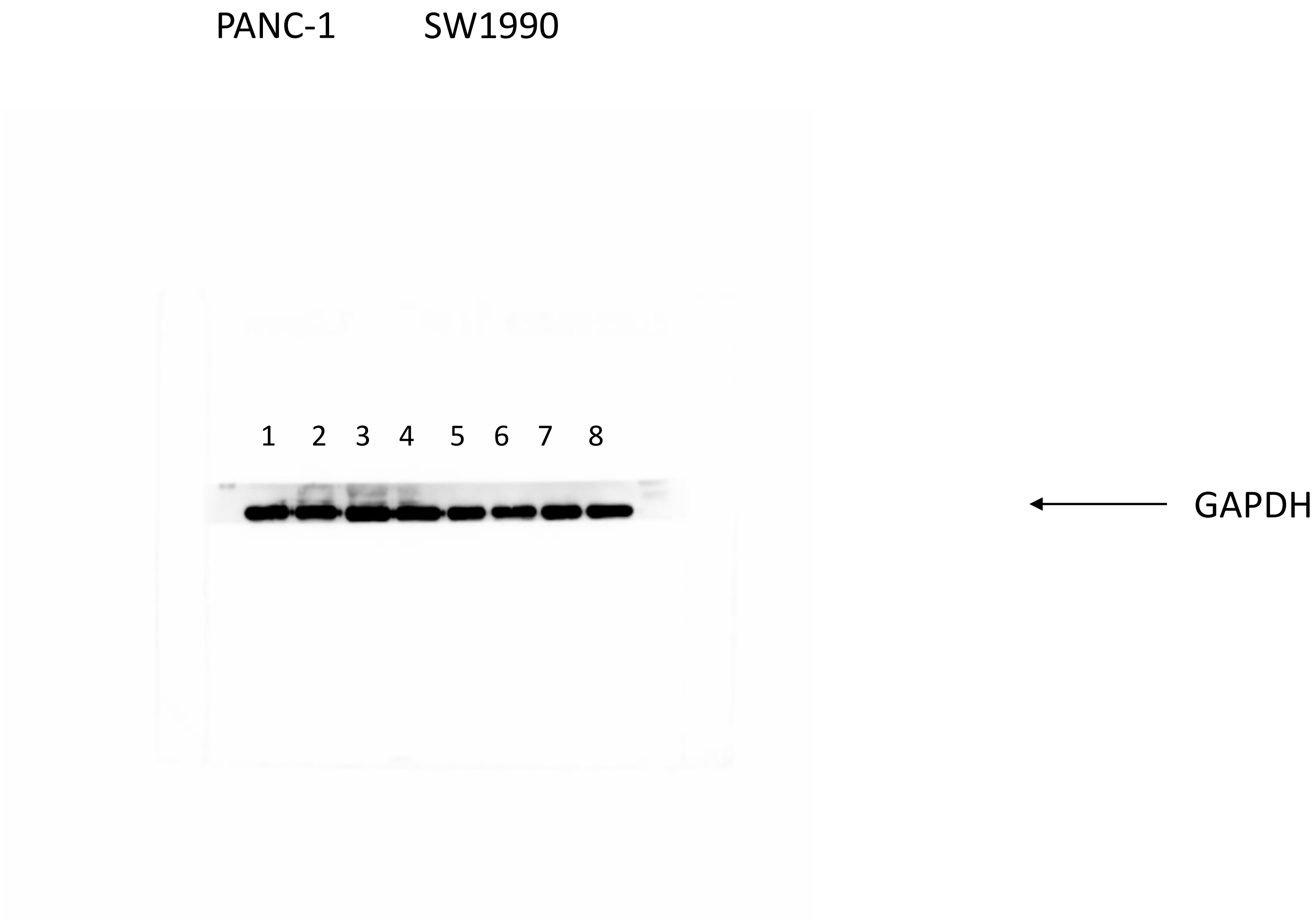

Full and uncropped western blot for Figure 2E

Lanes 1-8 are on the figure

PANC-1 SW1990

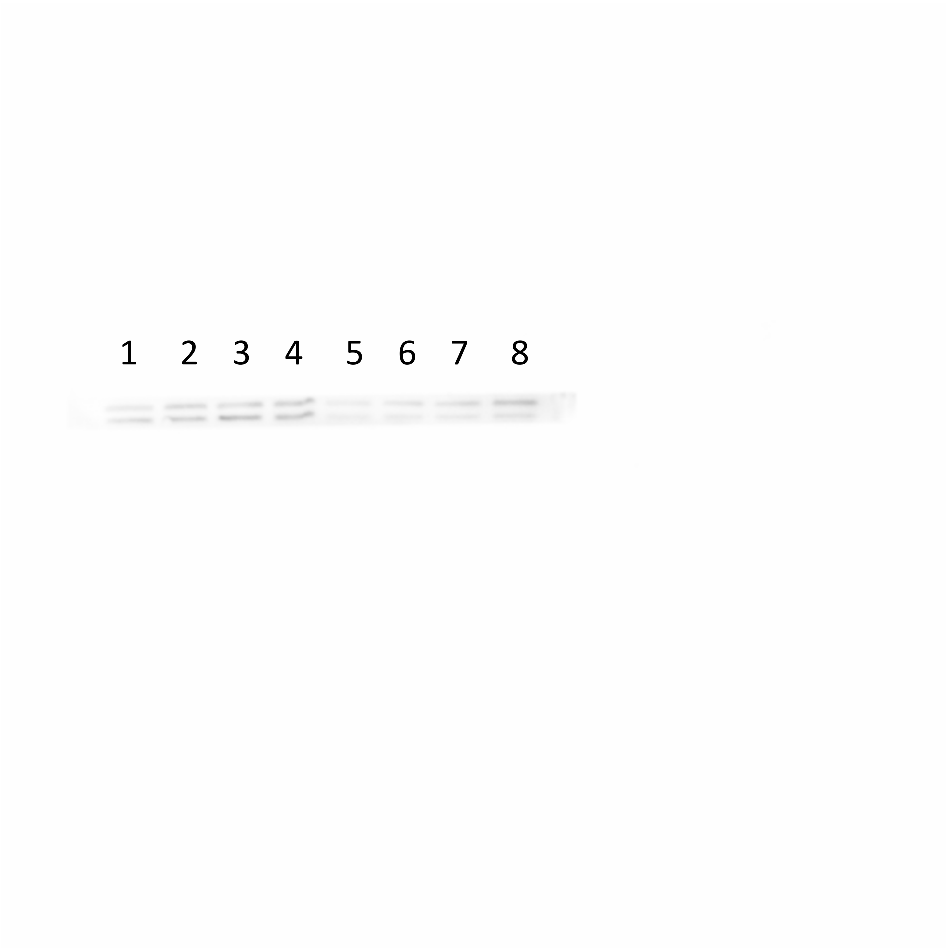

P-ATM

Full and uncropped western blot for Figure 2E

Lanes 1-8 are on the figure

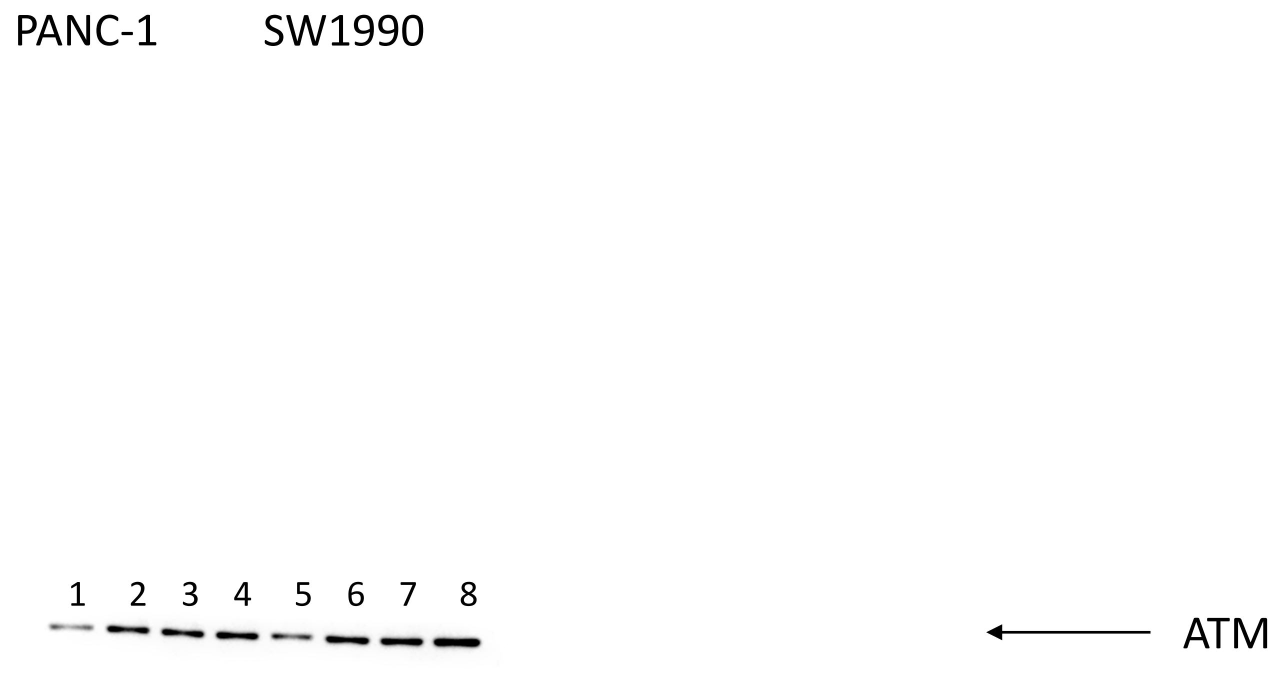

Full and uncropped western blot for Figure 2E

Lanes 1-8 are on the figure

PANC-1 SW1990

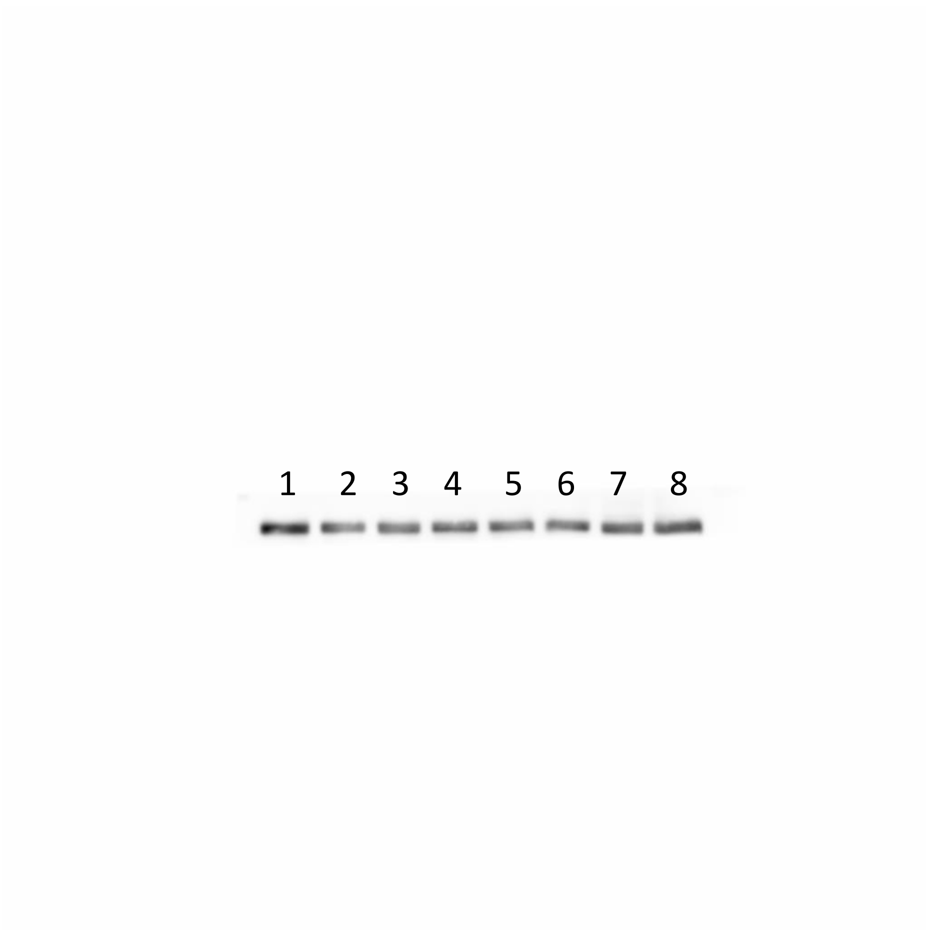

GAPDH

Full and uncropped western blot for Figure 2F

Lanes 1-8 are on the figure

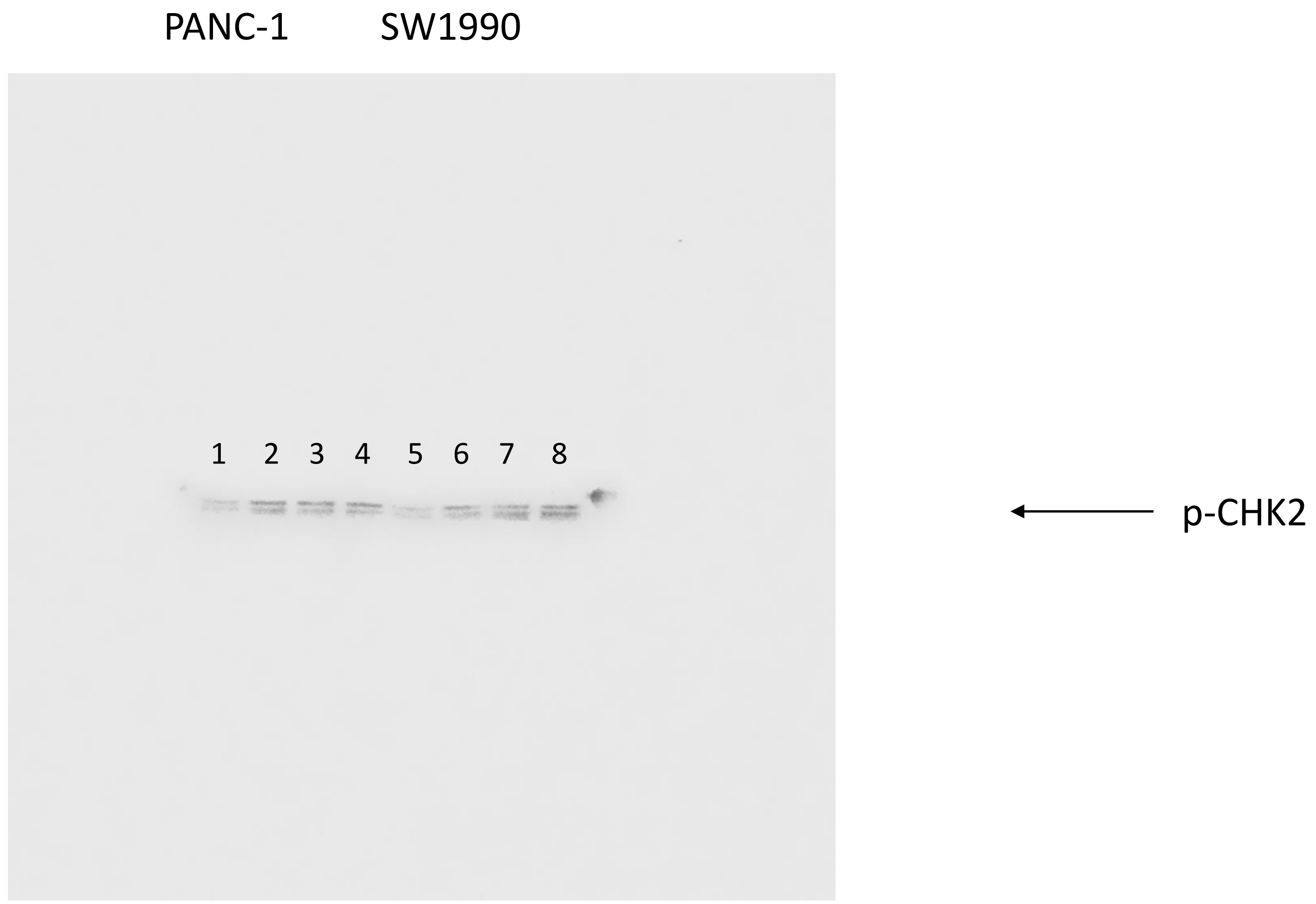

Full and uncropped western blot for Figure 2F

Lanes 1-8 are on the figure

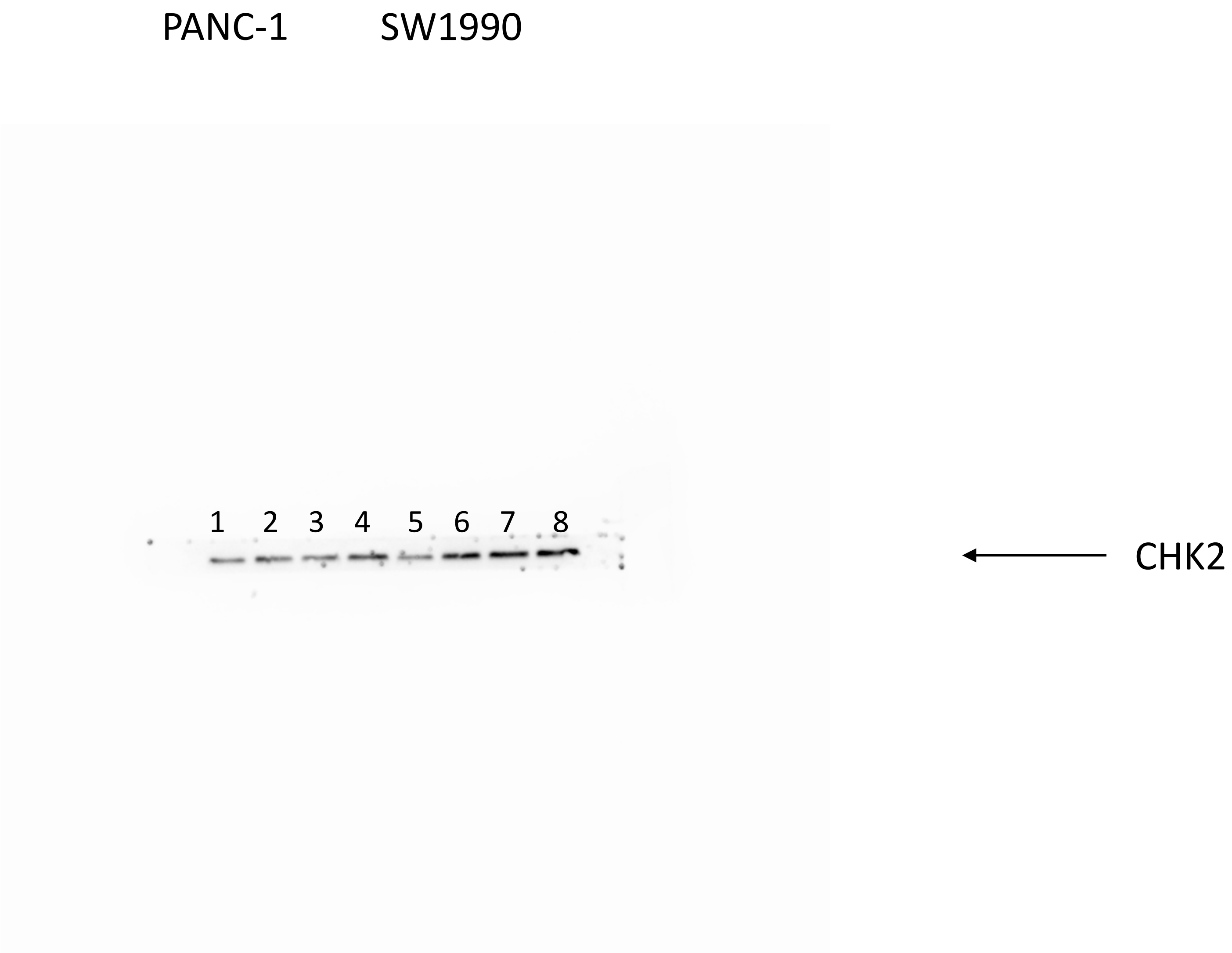

Full and uncropped western blot for Figure 2F

Lanes 1-8 are on the figure

PANC-1 SW1990

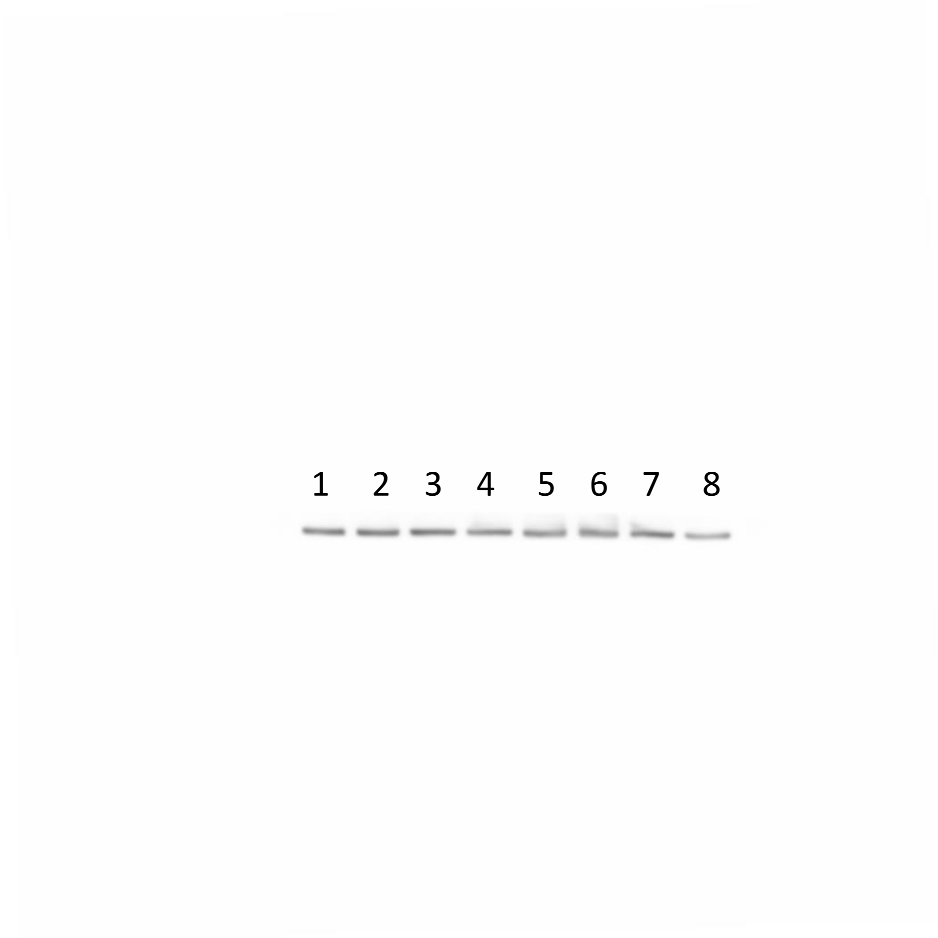

GAPDH

Full and uncropped western blot for Figure 2G

Lanes 1-8 are on the figure

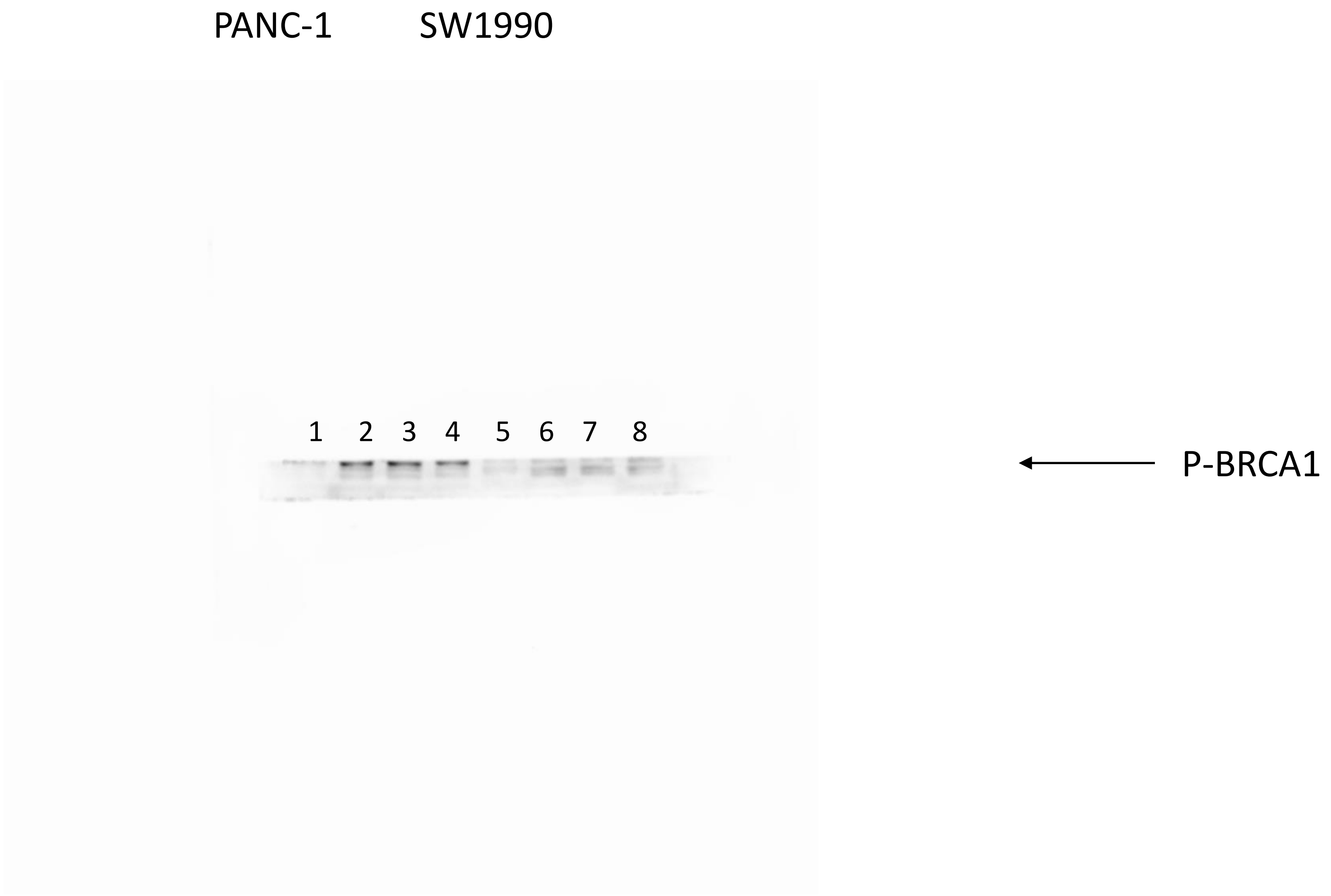

Full and uncropped western blot for Figure 2G

Lanes 1-8 are on the figure

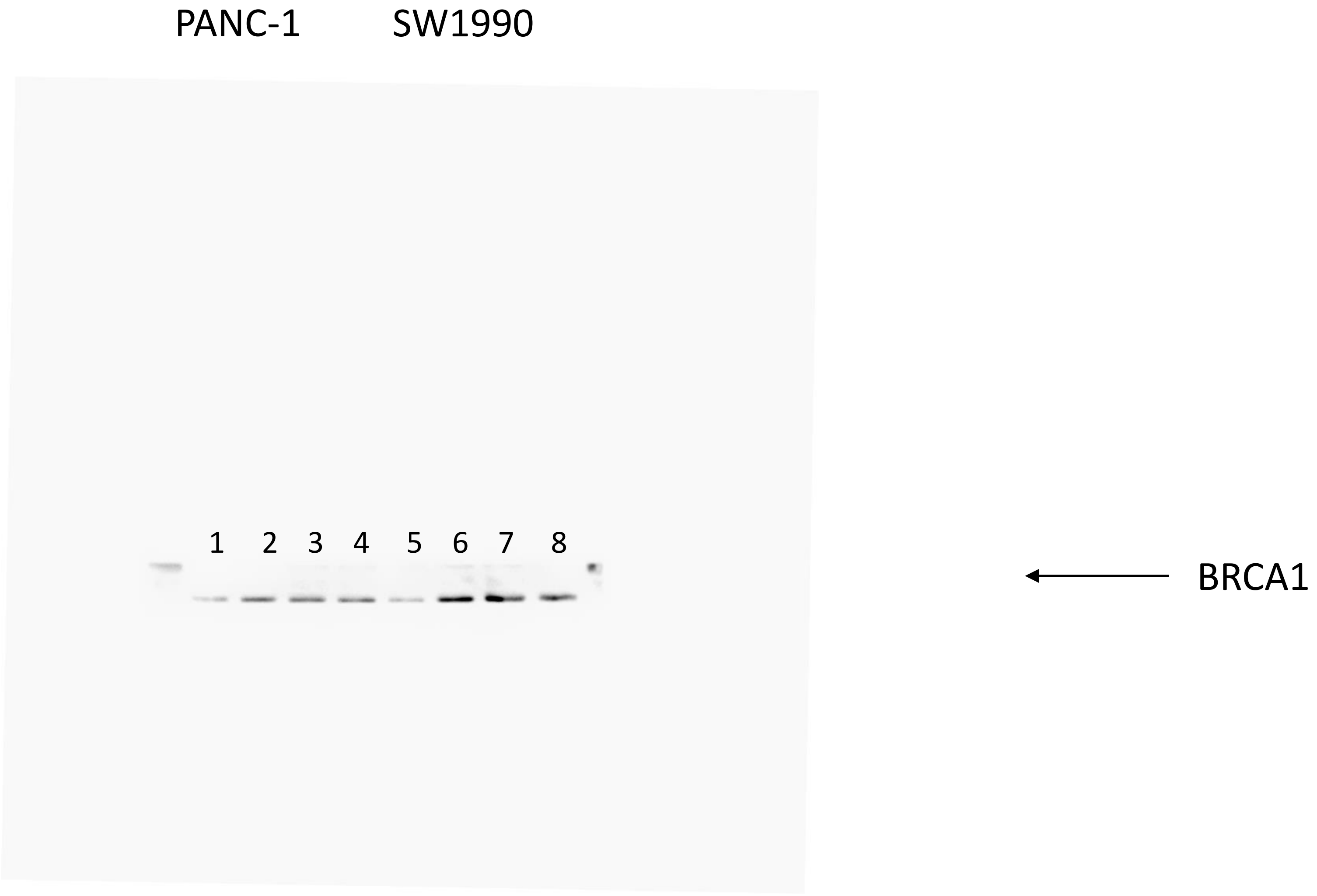

Full and uncropped western blot for Figure 2G

Lanes 1-8 are on the figure

PANC-1 SW1990

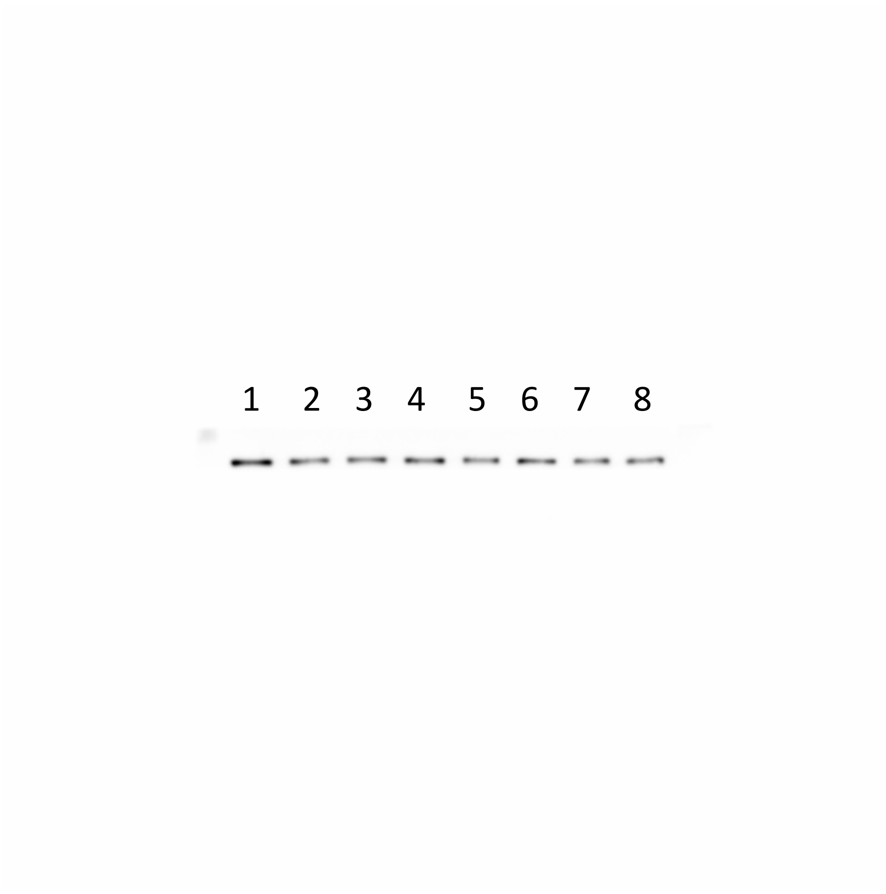

GAPDH

Full and uncropped western blot for Figure 2N

Lanes 1-8 are on the figure

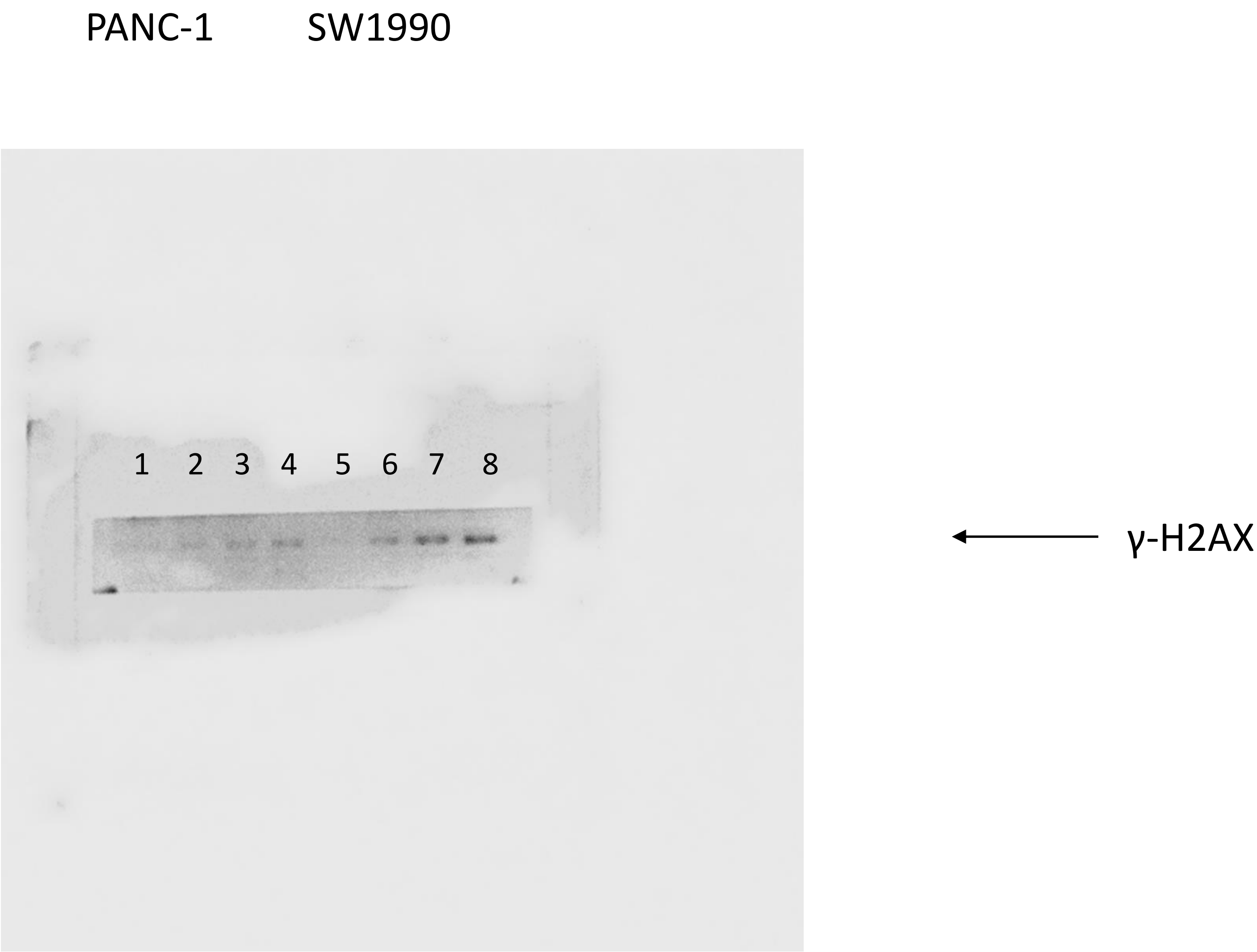

Full and uncropped western blot for Figure 2N

Lanes 1-8 are on the figure

PANC-1 SW1990

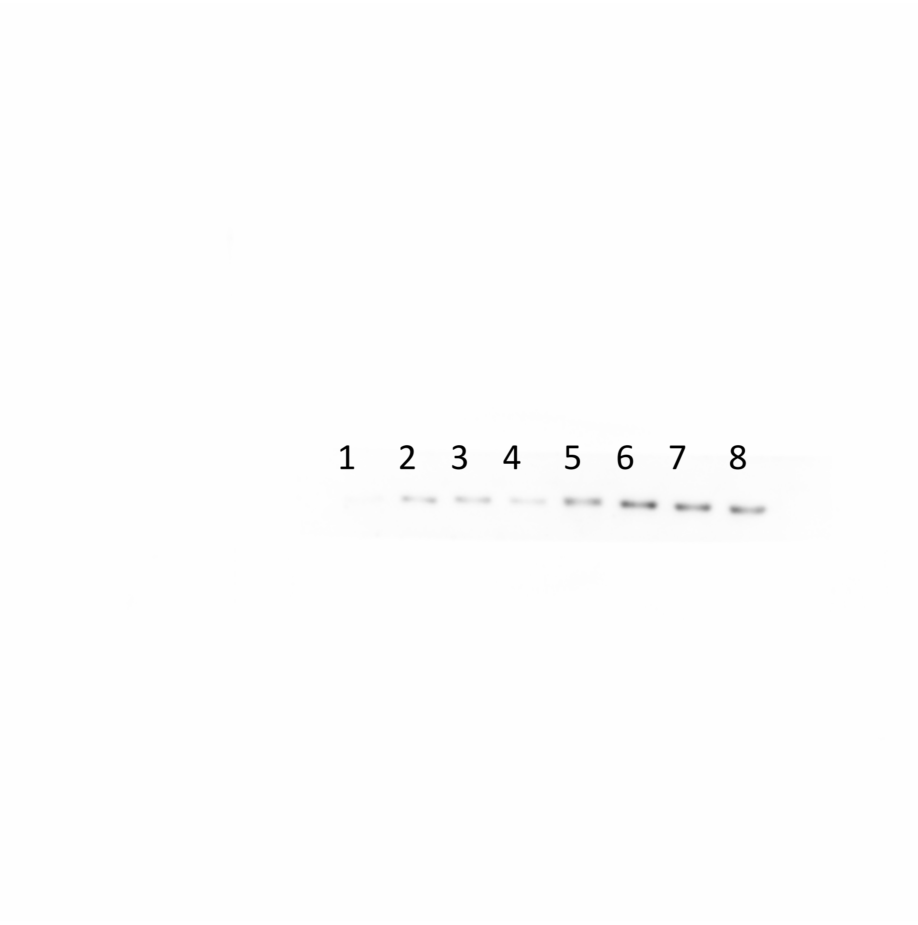

53BP1

Full and uncropped western blot for Figure 2N

Lanes 1-8 are on the figure

PANC-1 SW1990

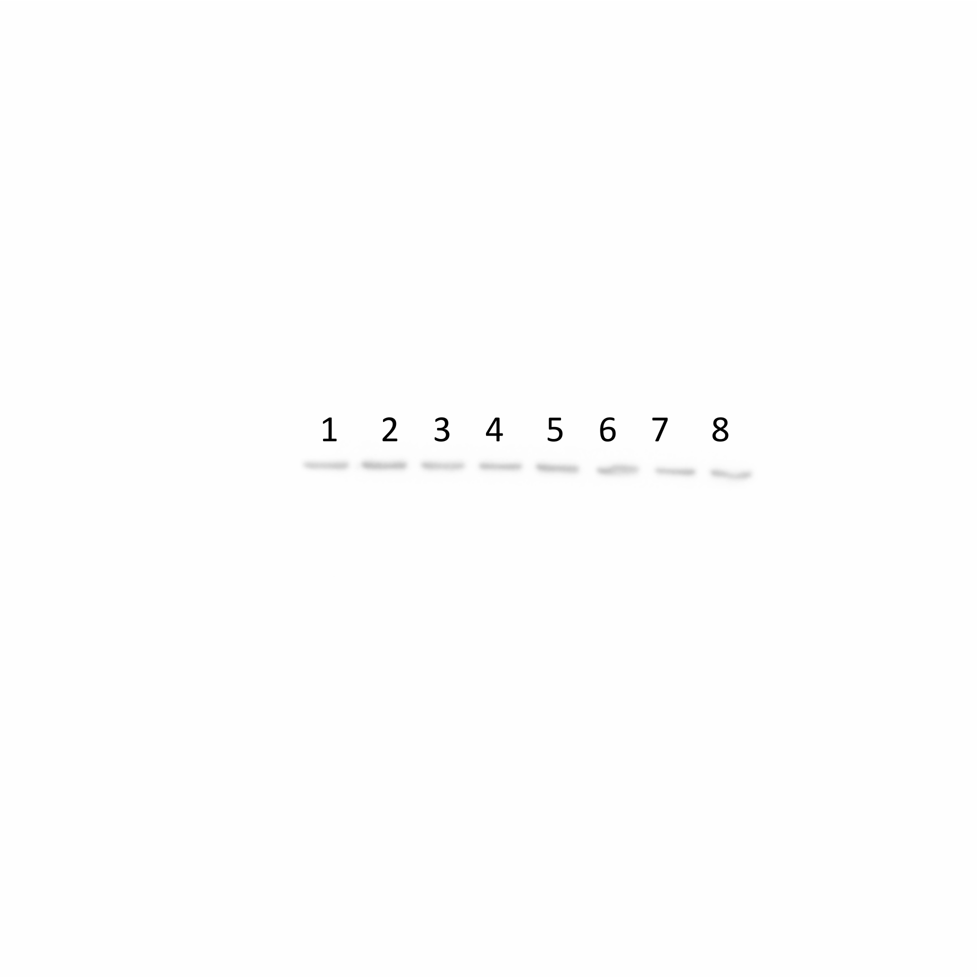

GAPDH

Full and uncropped western blot for Figure 2N

Lanes 1-8 are on the figure

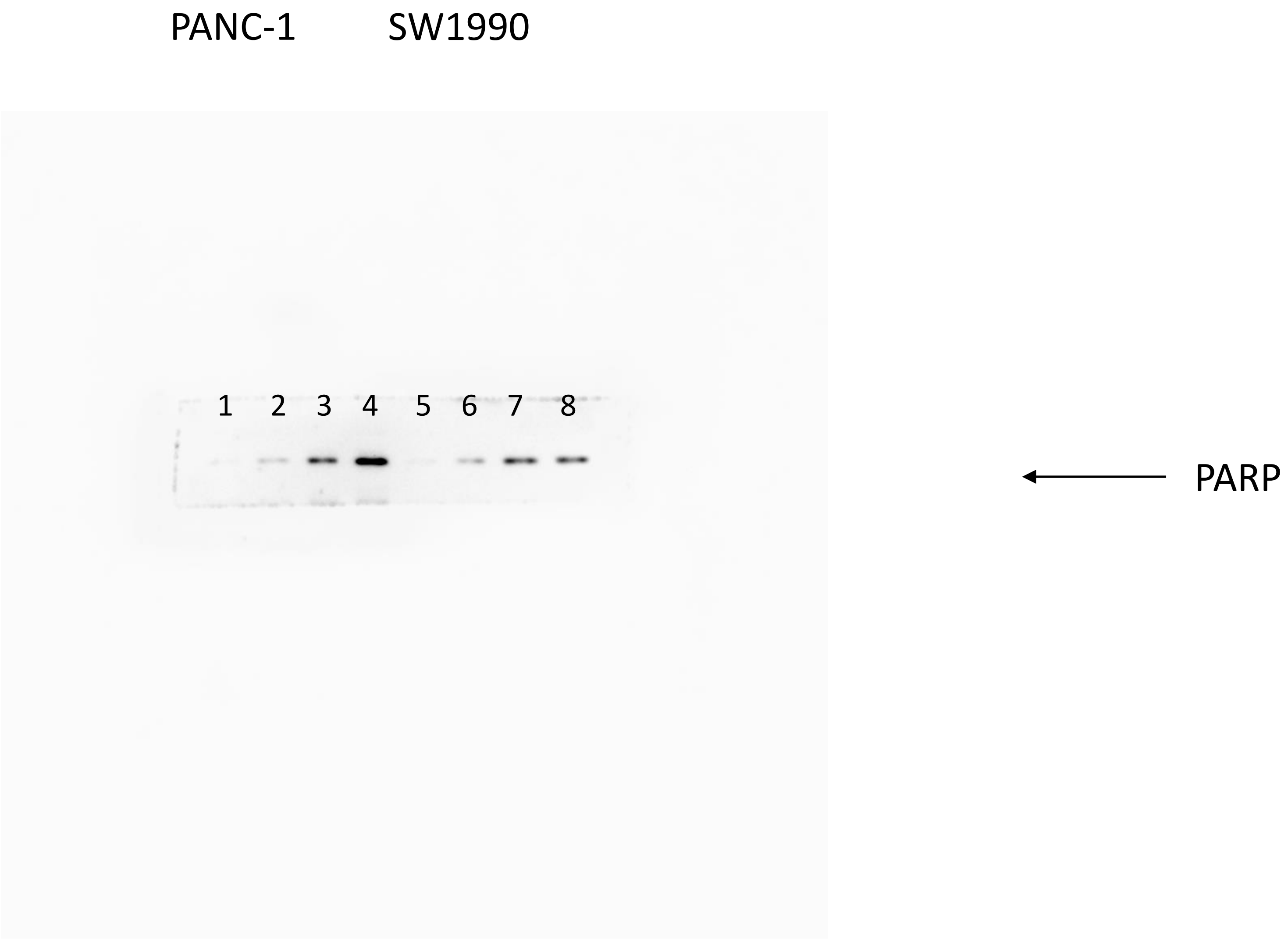

Full and uncropped western blot for Figure 2N

Lanes 1-8 are on the figure

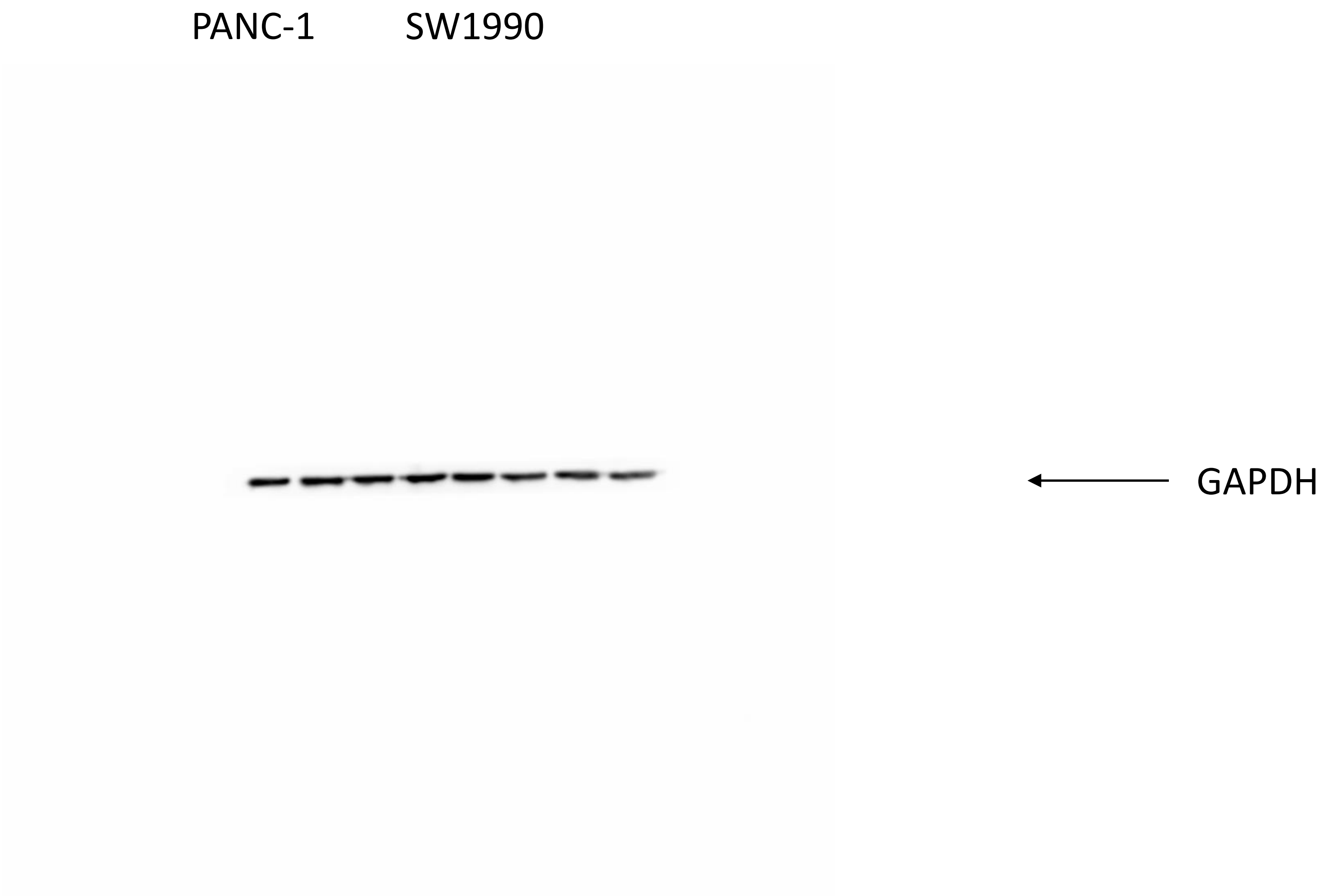

Full and uncropped western blot for Figure 3F

Lanes 1-8 are on the figure

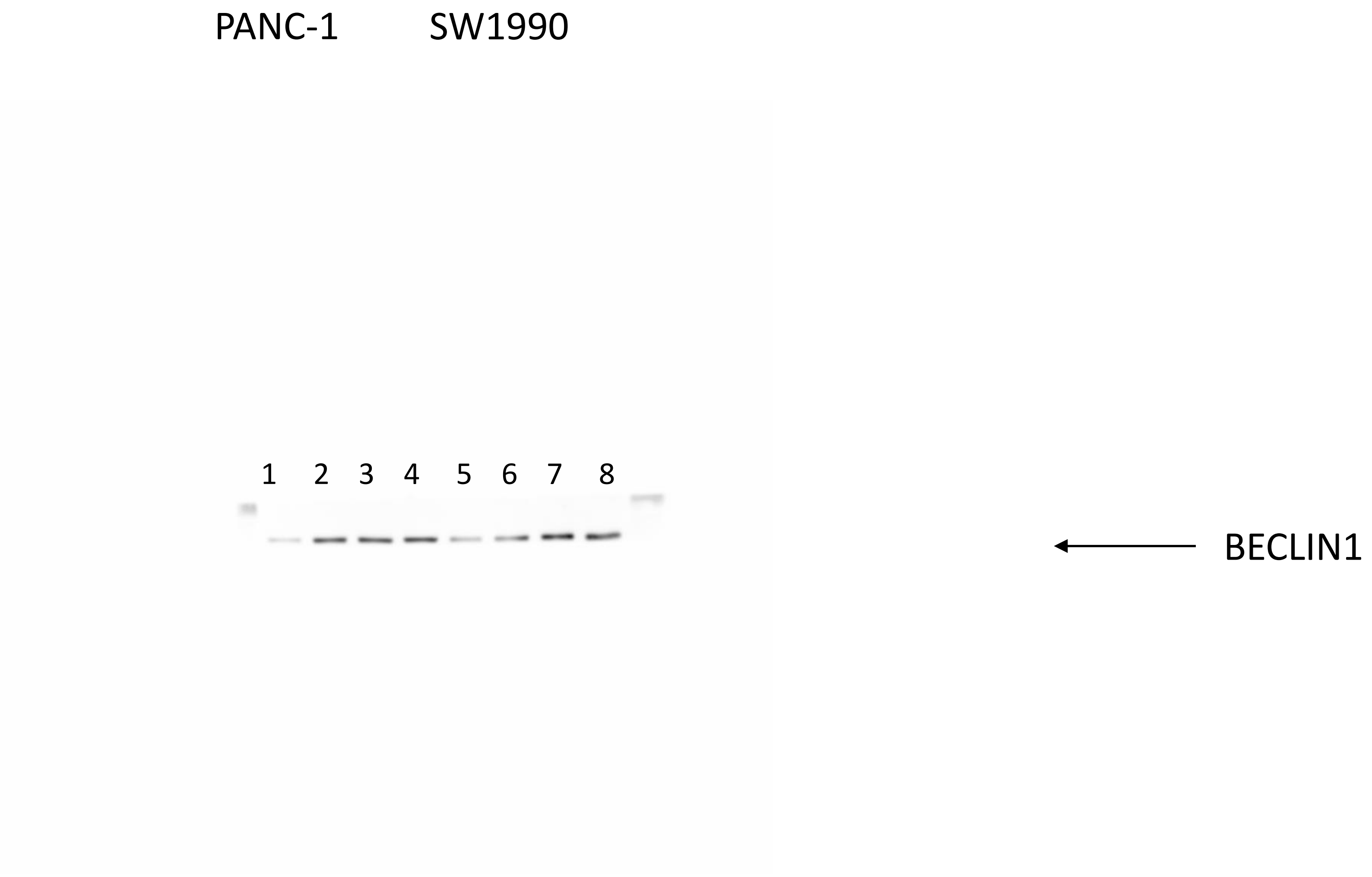

Full and uncropped western blot for Figure 3F

Lanes 1-8 are on the figure

PANC-1 SW1990

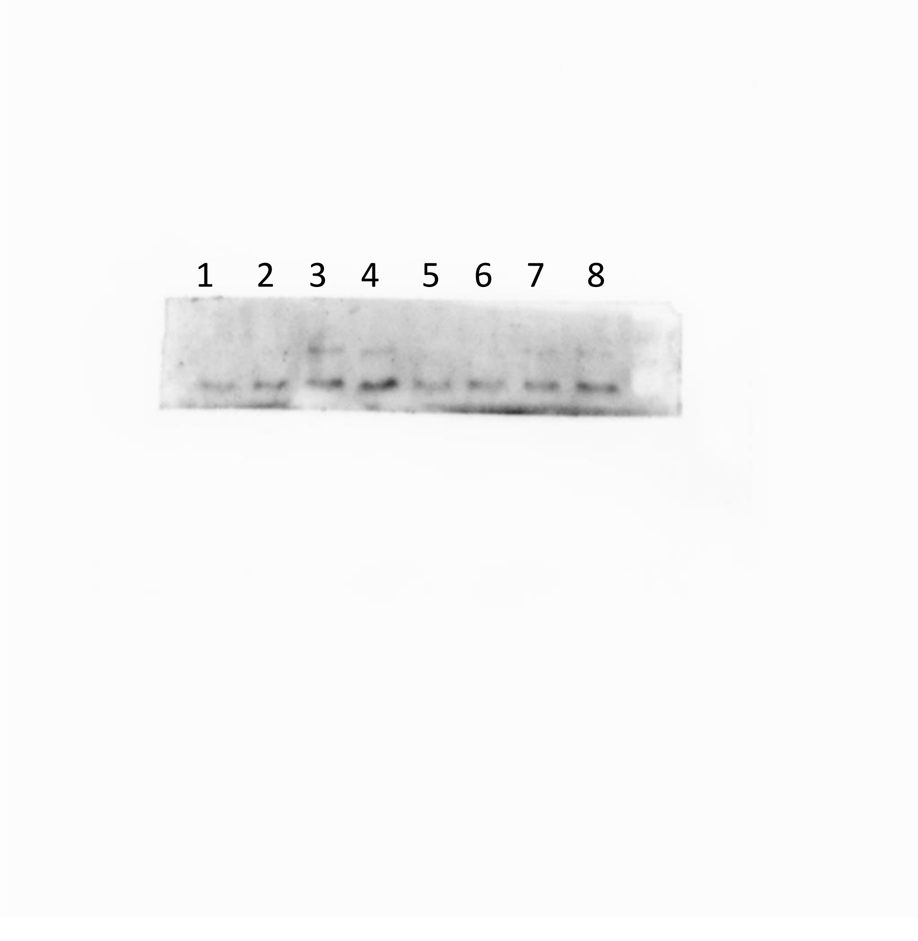

LC3B

Full and uncropped western blot for Figure 3F

Lanes 1-8 are on the figure

PANC-1 SW1990

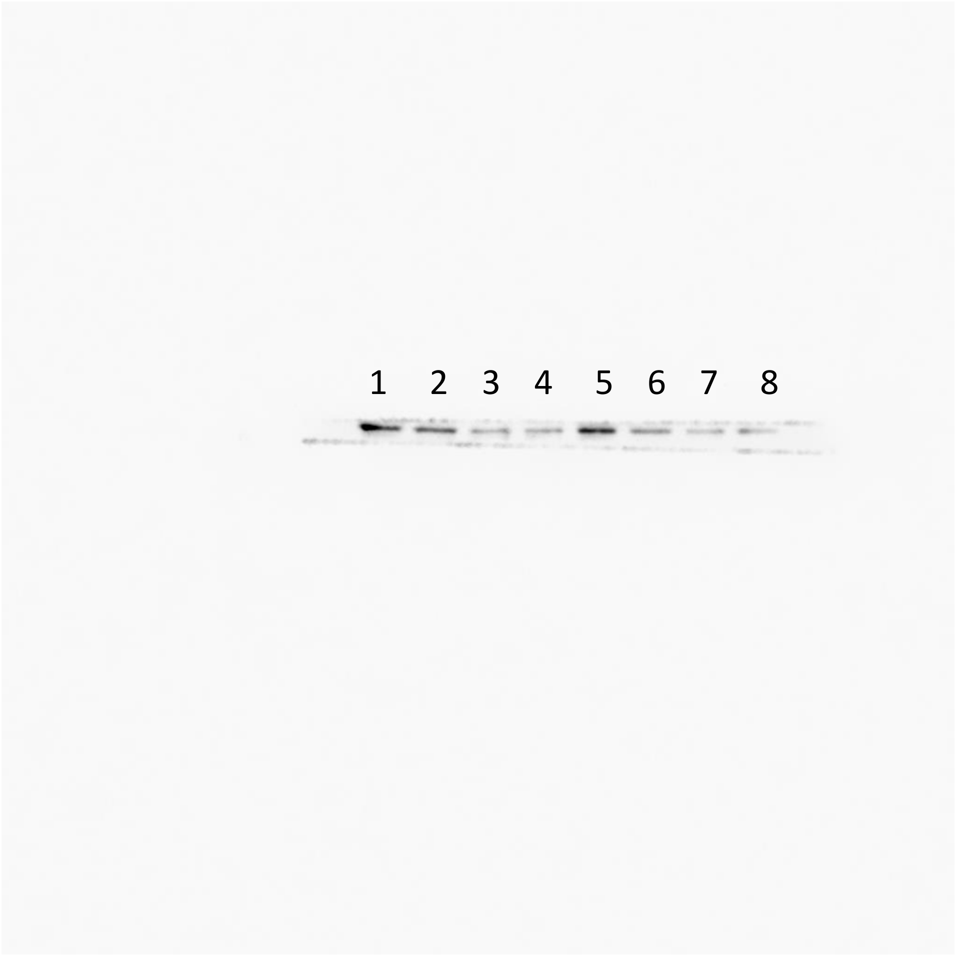

Full and uncropped western blot for Figure 3F

Lanes 1-8 are on the figure

PANC-1 SW1990

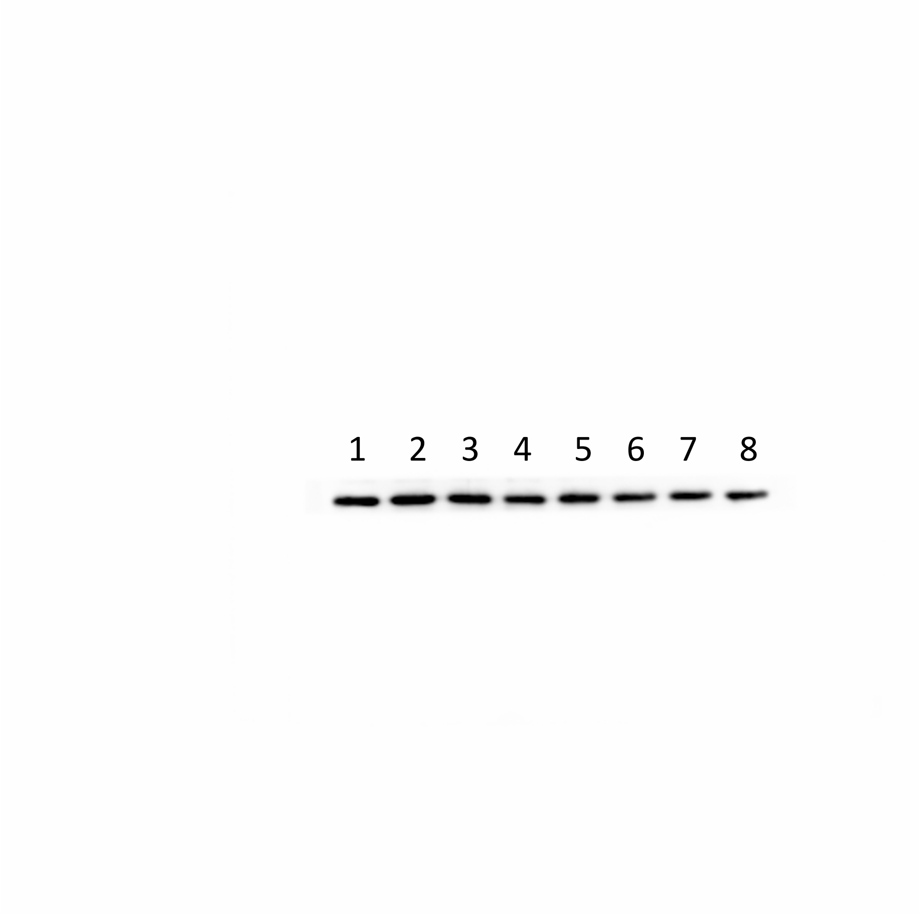

GAPDH

Full and uncropped western blot for Figure 3G

Lanes 1-8 are on the figure

PANC-1 SW1990

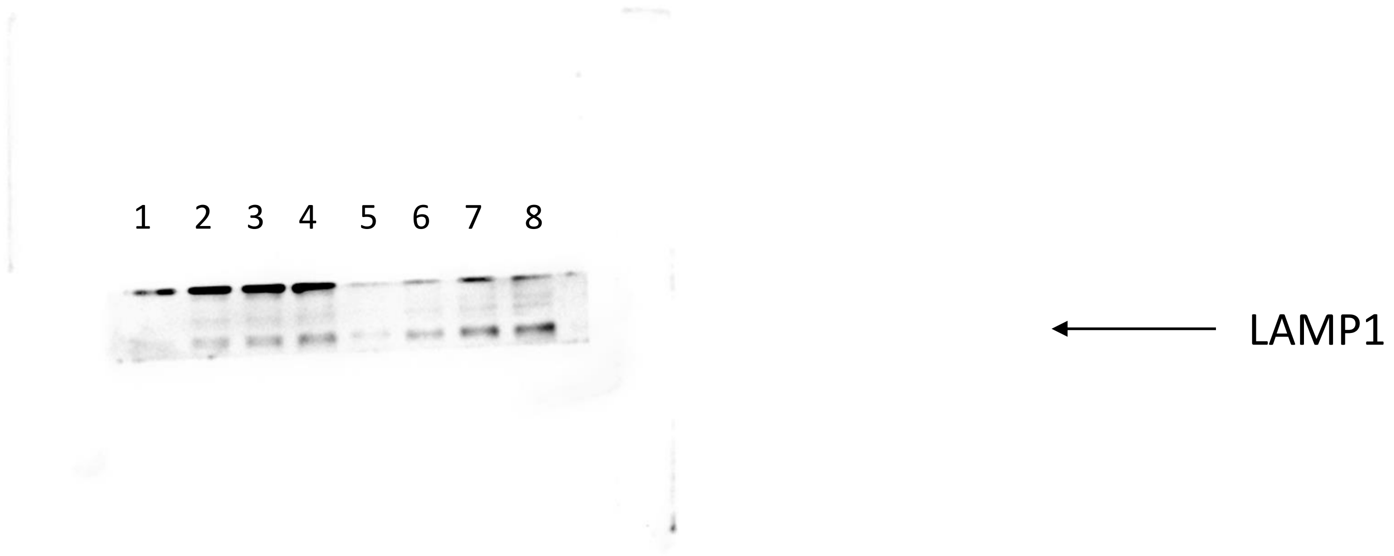

Full and uncropped western blot for Figure 3G

Lanes 1-8 are on the figure

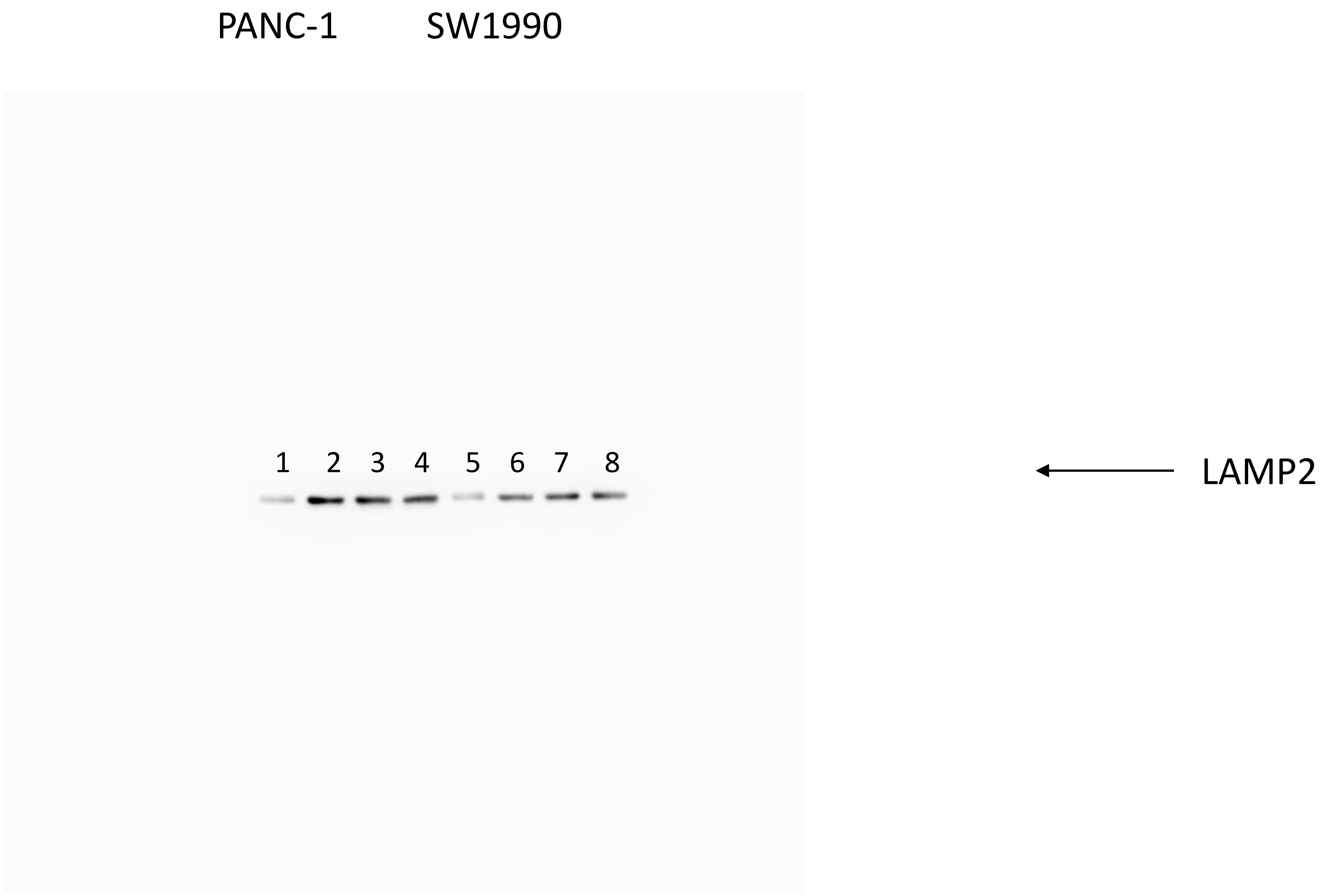

Full and uncropped western blot for Figure 3G

Lanes 1-8 are on the figure

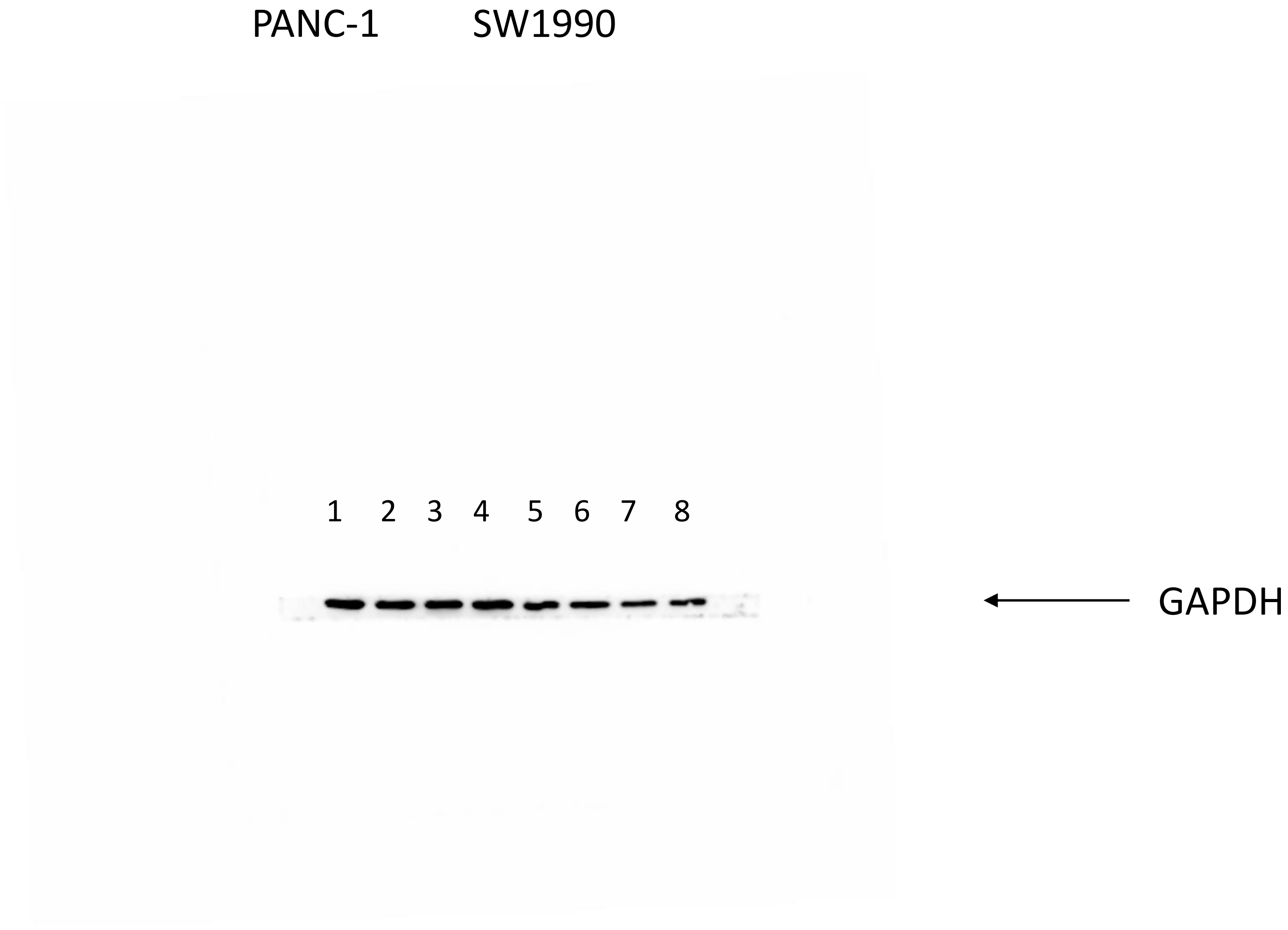

Full and uncropped western blot for Figure 3H

Lanes 1-8 are on the figure

PANC-1 SW1990

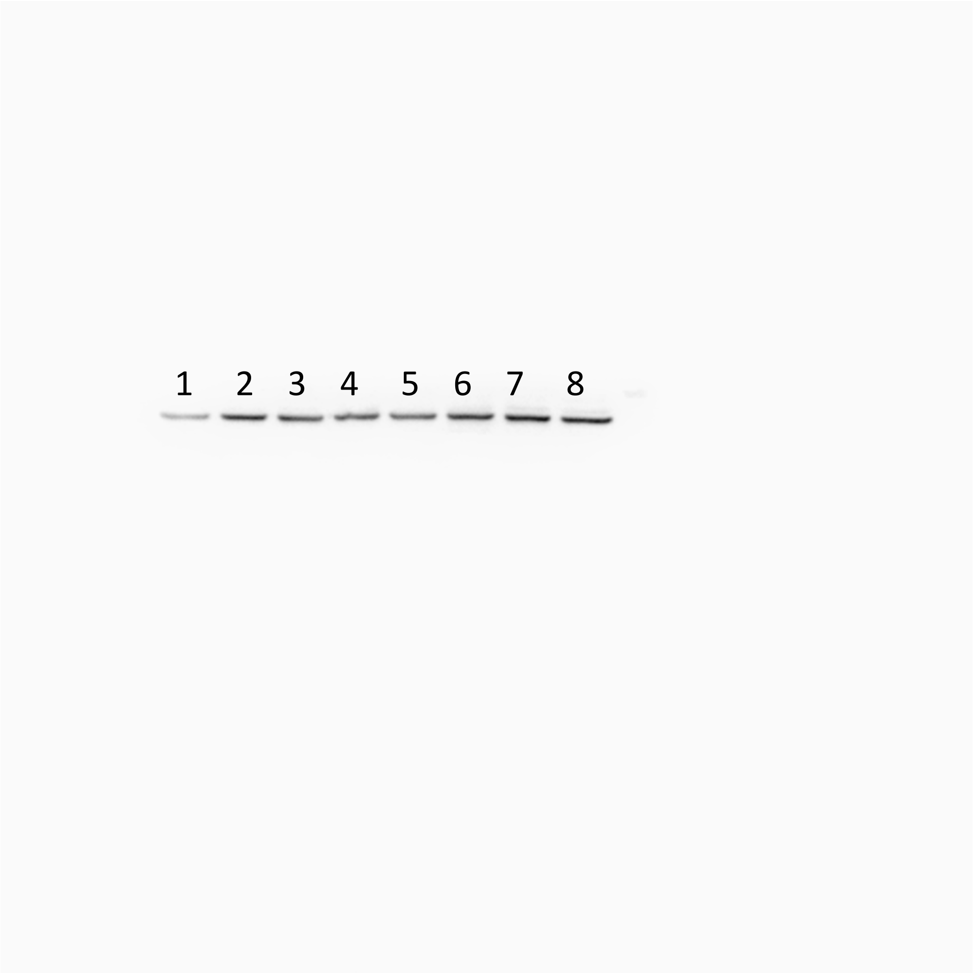

Parkin

Full and uncropped western blot for Figure 3H

Lanes 1-8 are on the figure

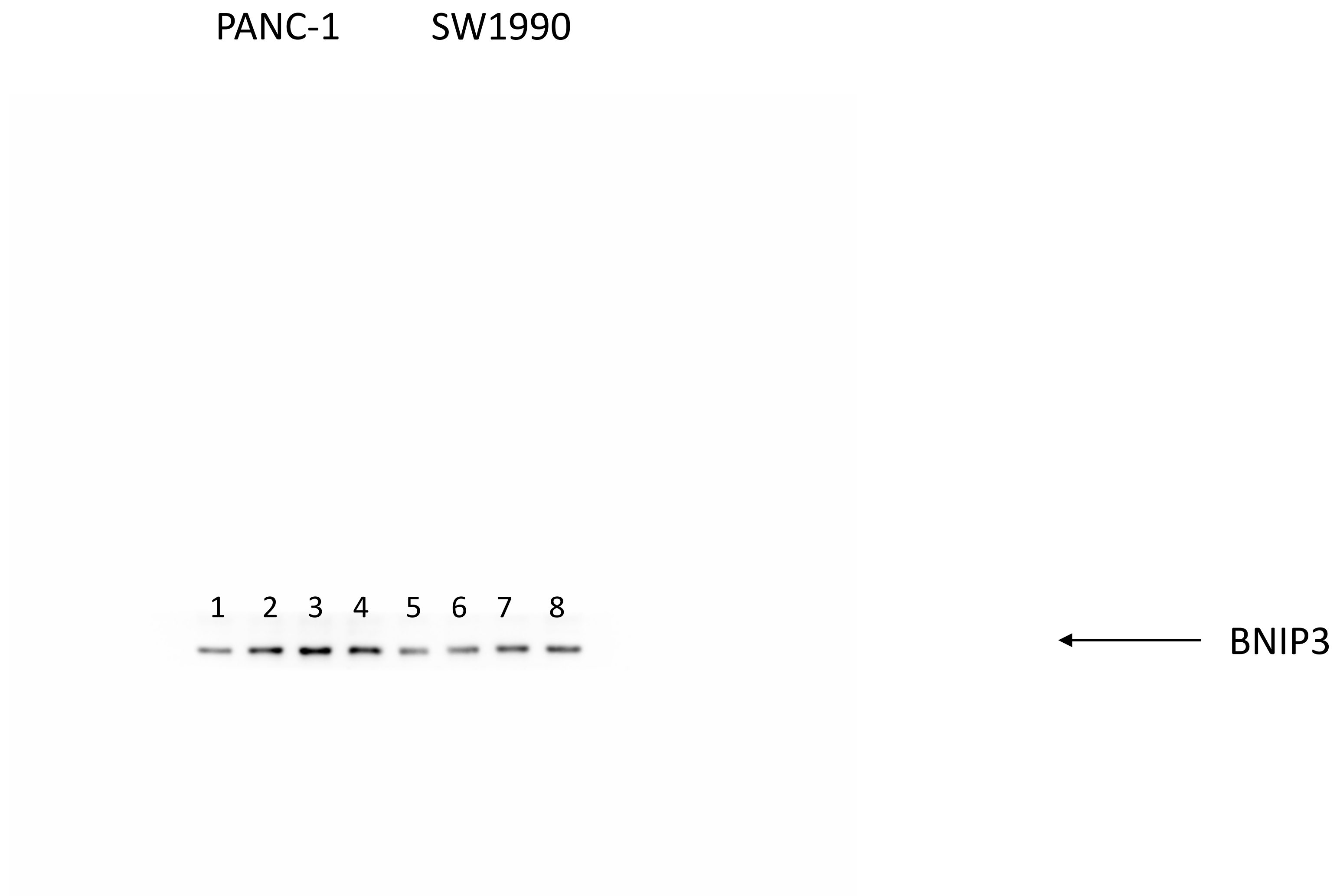

Full and uncropped western blot for Figure 3H

Lanes 1-8 are on the figure

PANC-1 SW1990

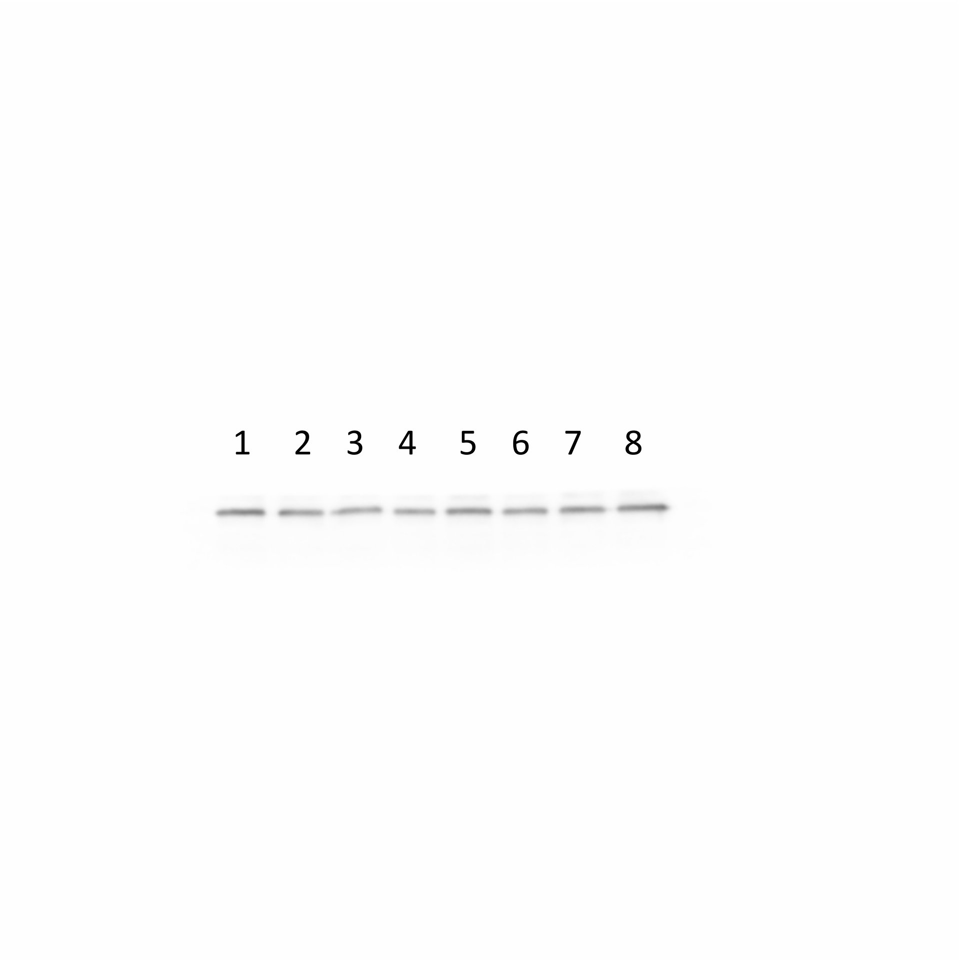

GAPDH

Full and uncropped western blot for Figure 4A

Lanes 1-8 are on the figure

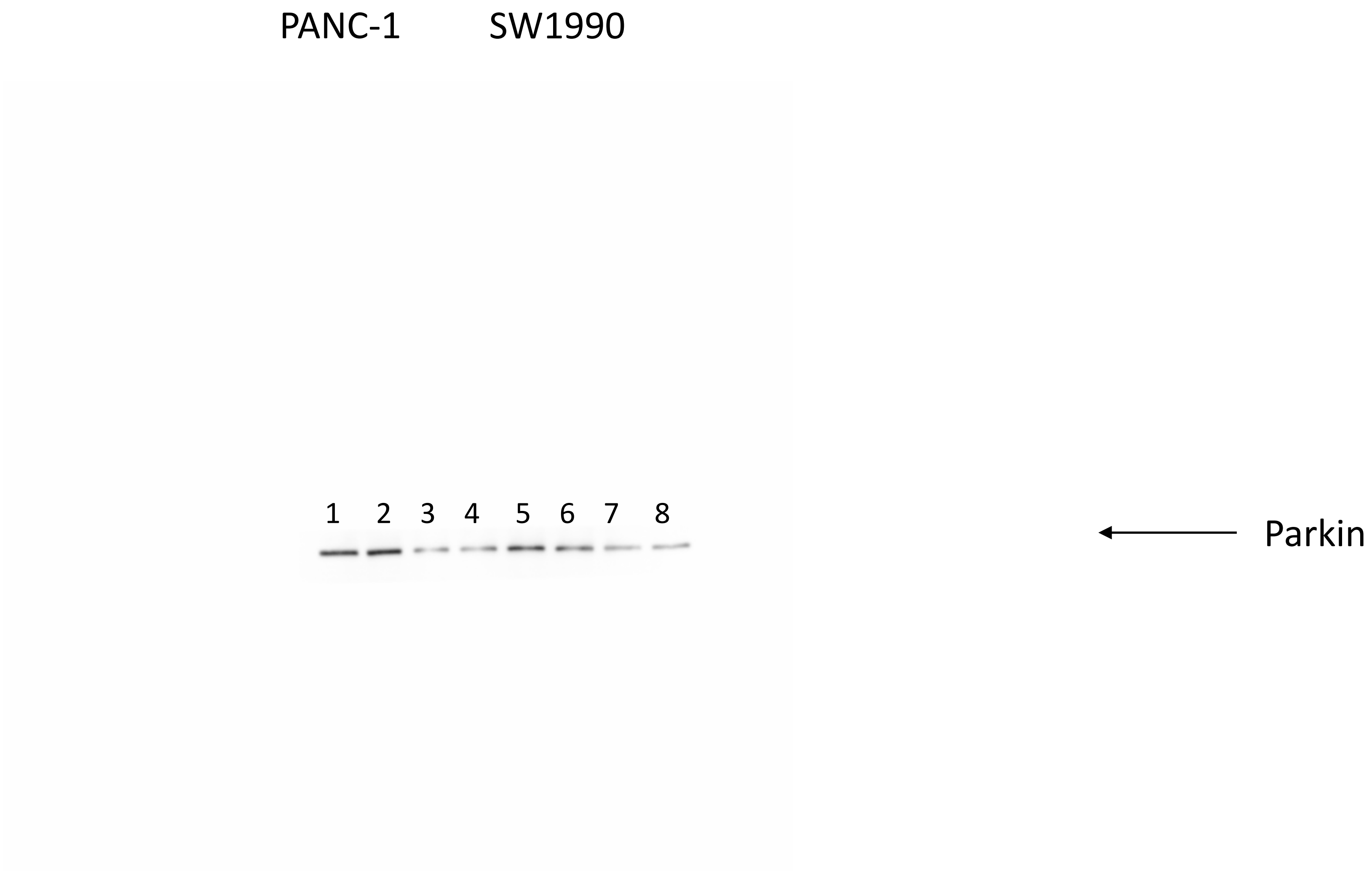

Full and uncropped western blot for Figure 4A

Lanes 1-8 are on the figure

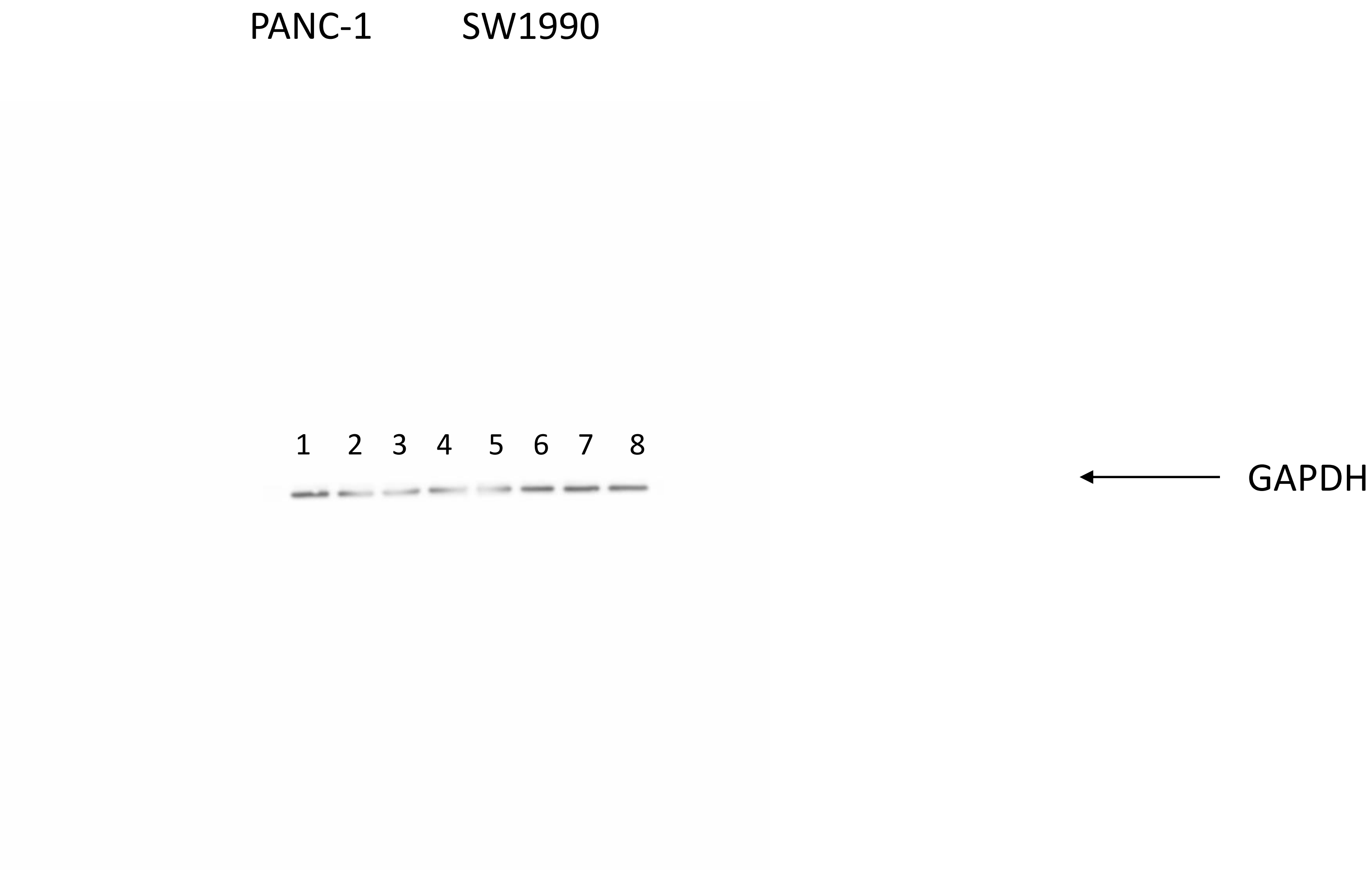

Full and uncropped western blot for Figure 4A

Lanes 1-8 are on the figure

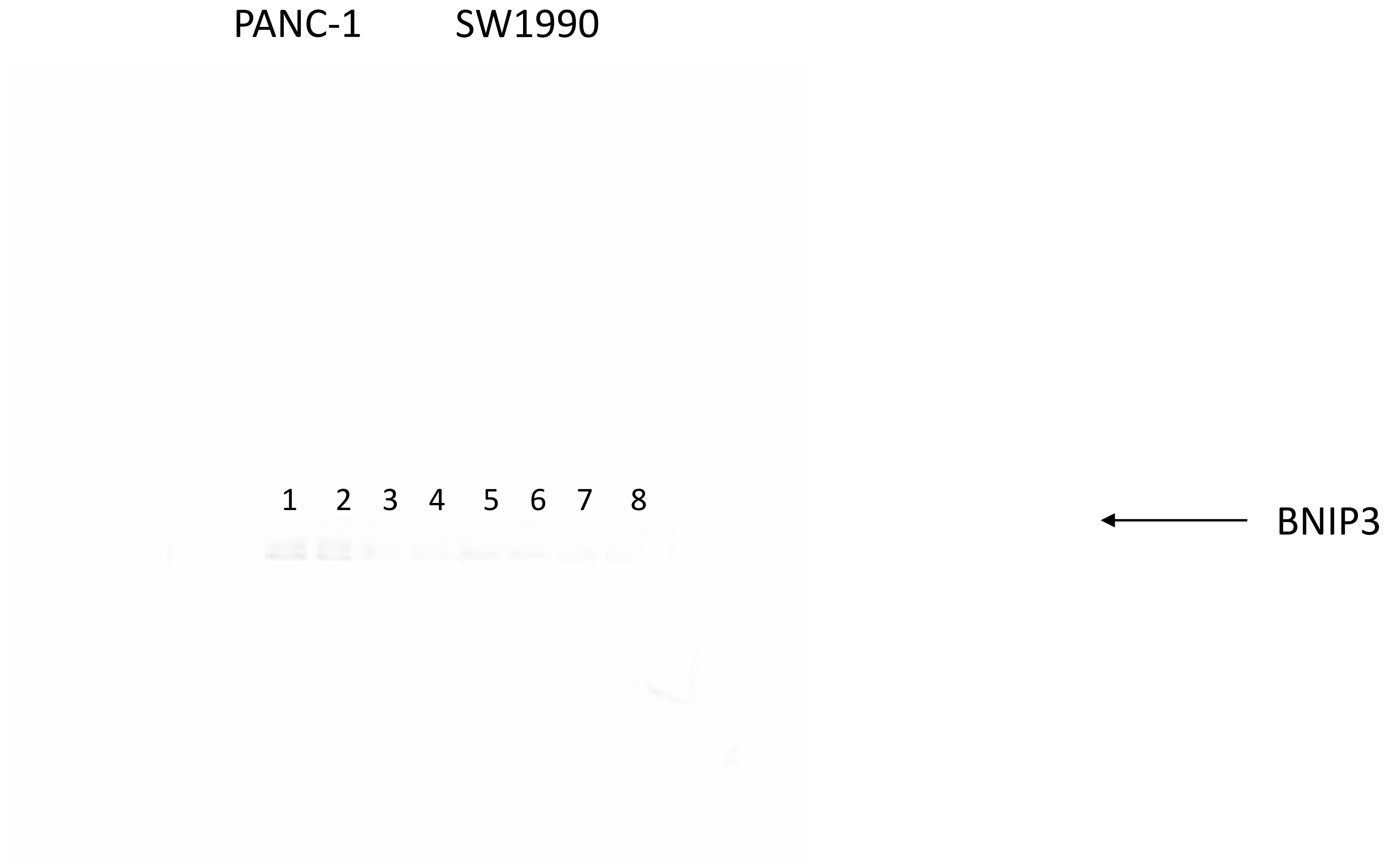

Full and uncropped western blot for Figure 4A

Lanes 1-8 are on the figure

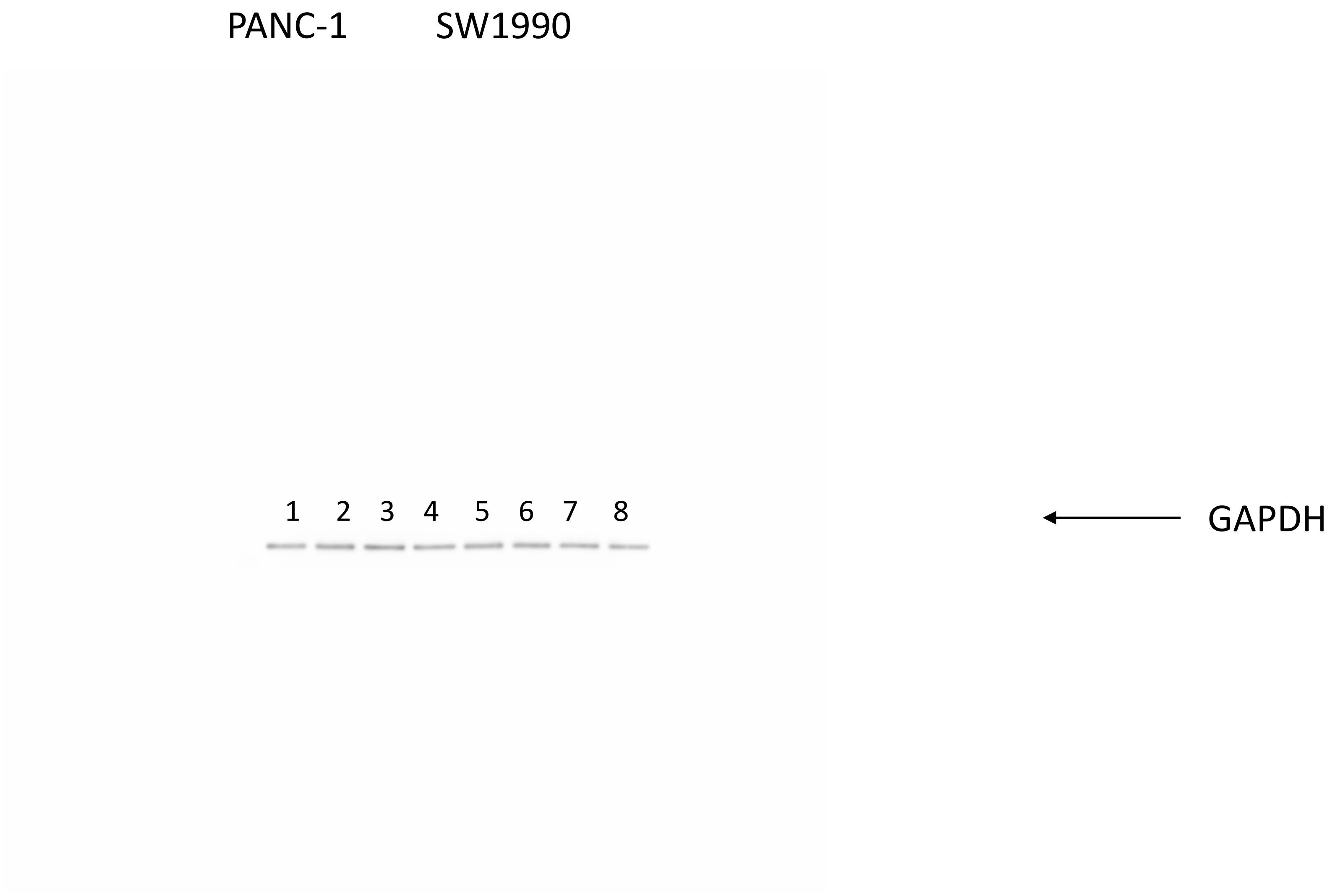

Full and uncropped western blot for Figure 4H

Lanes 1-8 are on the figure

PANC-1 SW1990

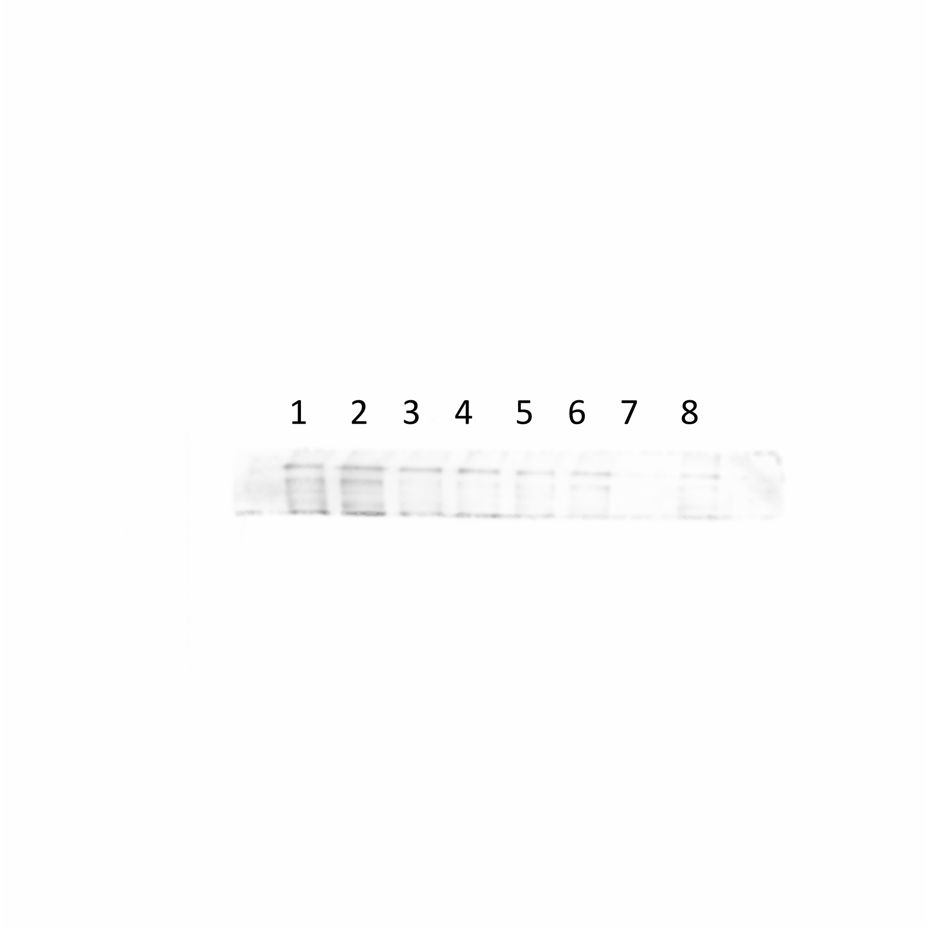

Parkin

Full and uncropped western blot for Figure 4H

Lanes 1-8 are on the figure

PANC-1 SW1990

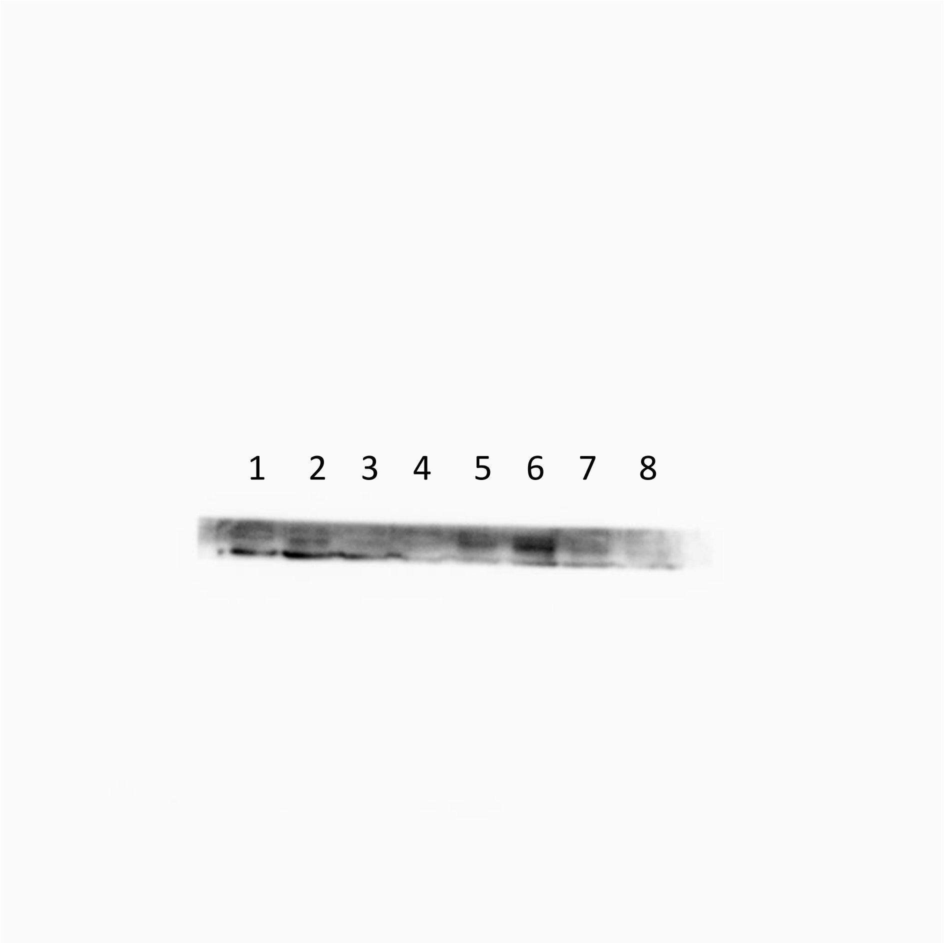

γ-H2AX

Full and uncropped western blot for Figure 4H

Lanes 1-8 are on the figure

PANC-1 SW1990

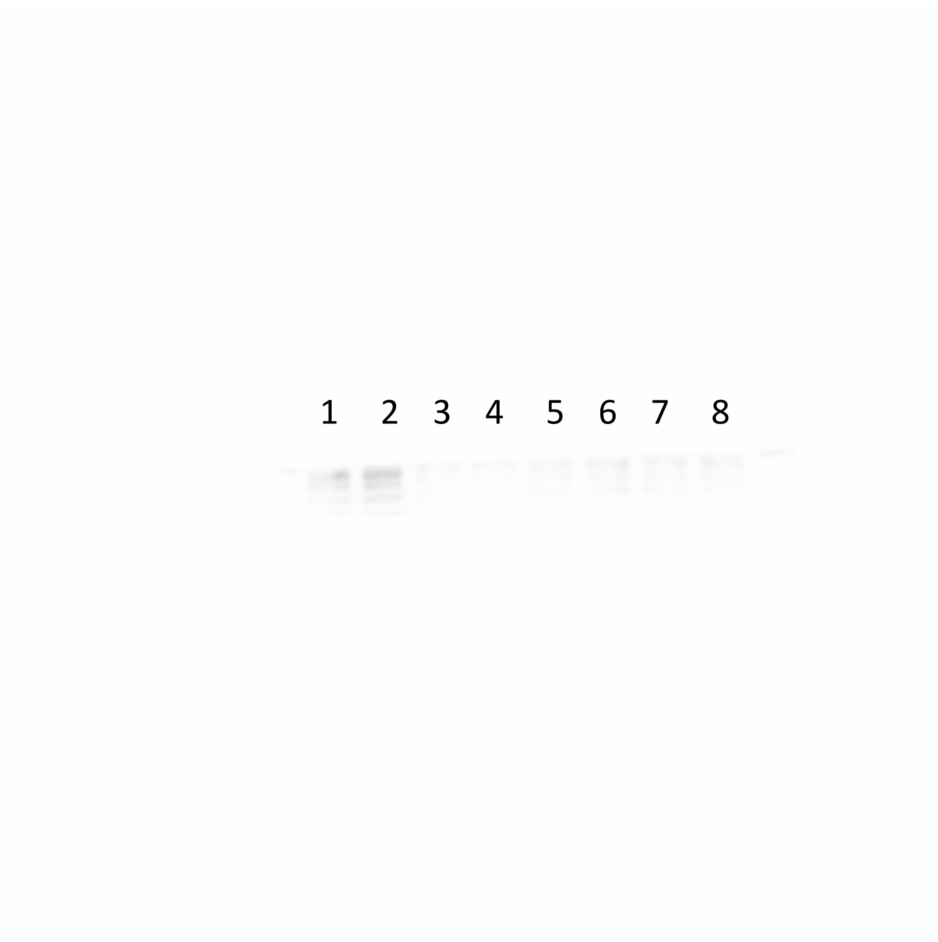

53BP1

Full and uncropped western blot for Figure 4H

Lanes 1-8 are on the figure

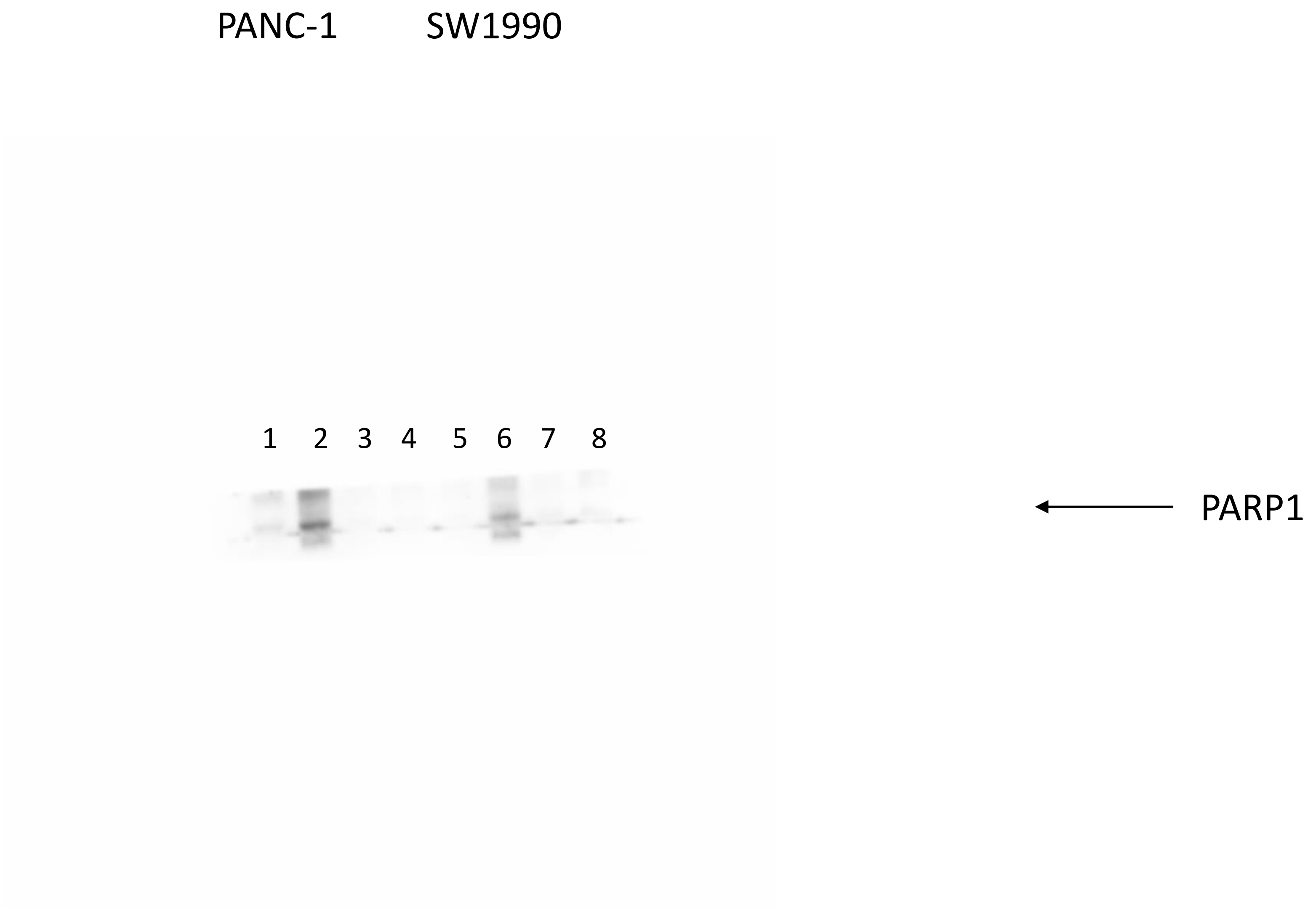

Full and uncropped western blot for Figure 4H

Lanes 1-8 are on the figure

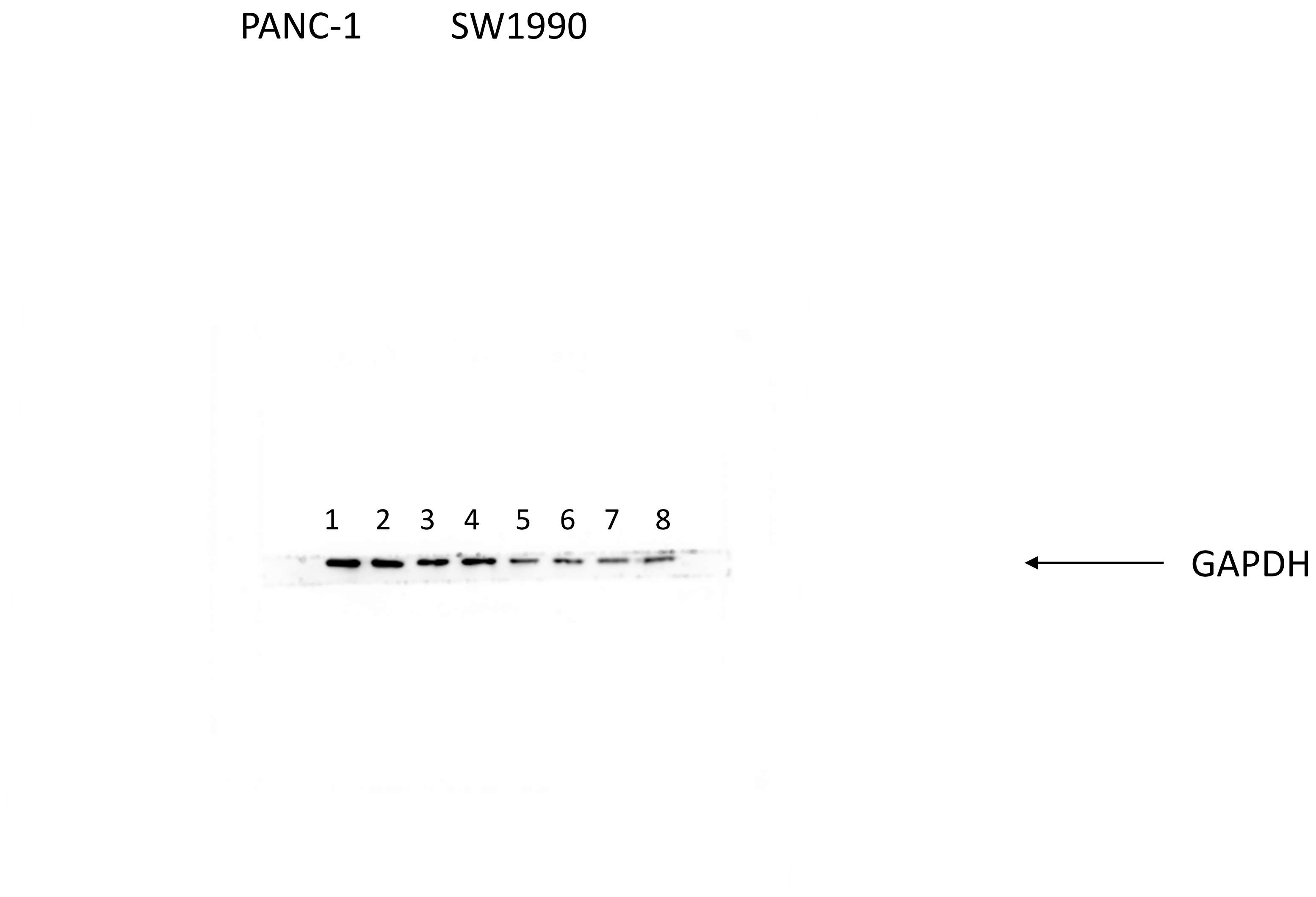

Full and uncropped western blot for Figure 4H

Lanes 1-8 are on the figure

PANC-1 SW1990

1 2 3 4 5 6 7 8

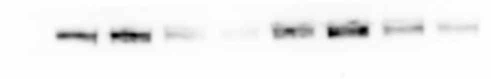

← BNIP3

Full and uncropped western blot for Figure 4H

Lanes 1-8 are on the figure

PANC-1 SW1990

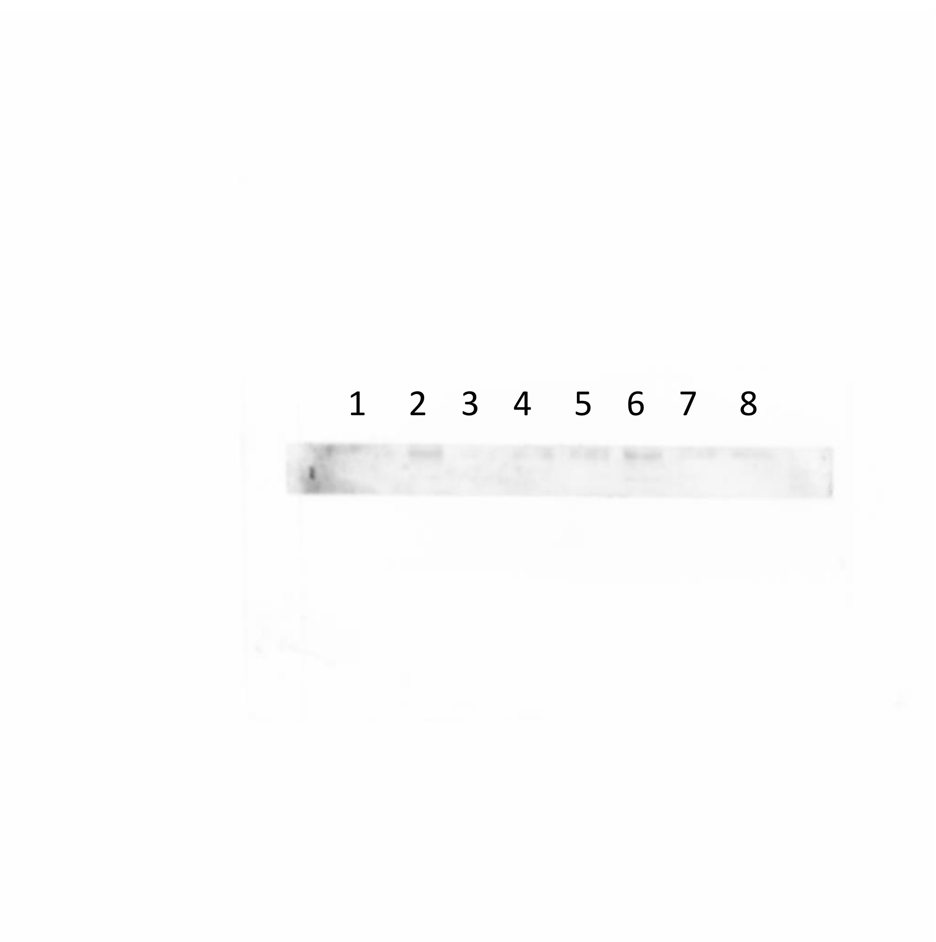

γ-H2AX

Full and uncropped western blot for Figure 4H

Lanes 1-8 are on the figure

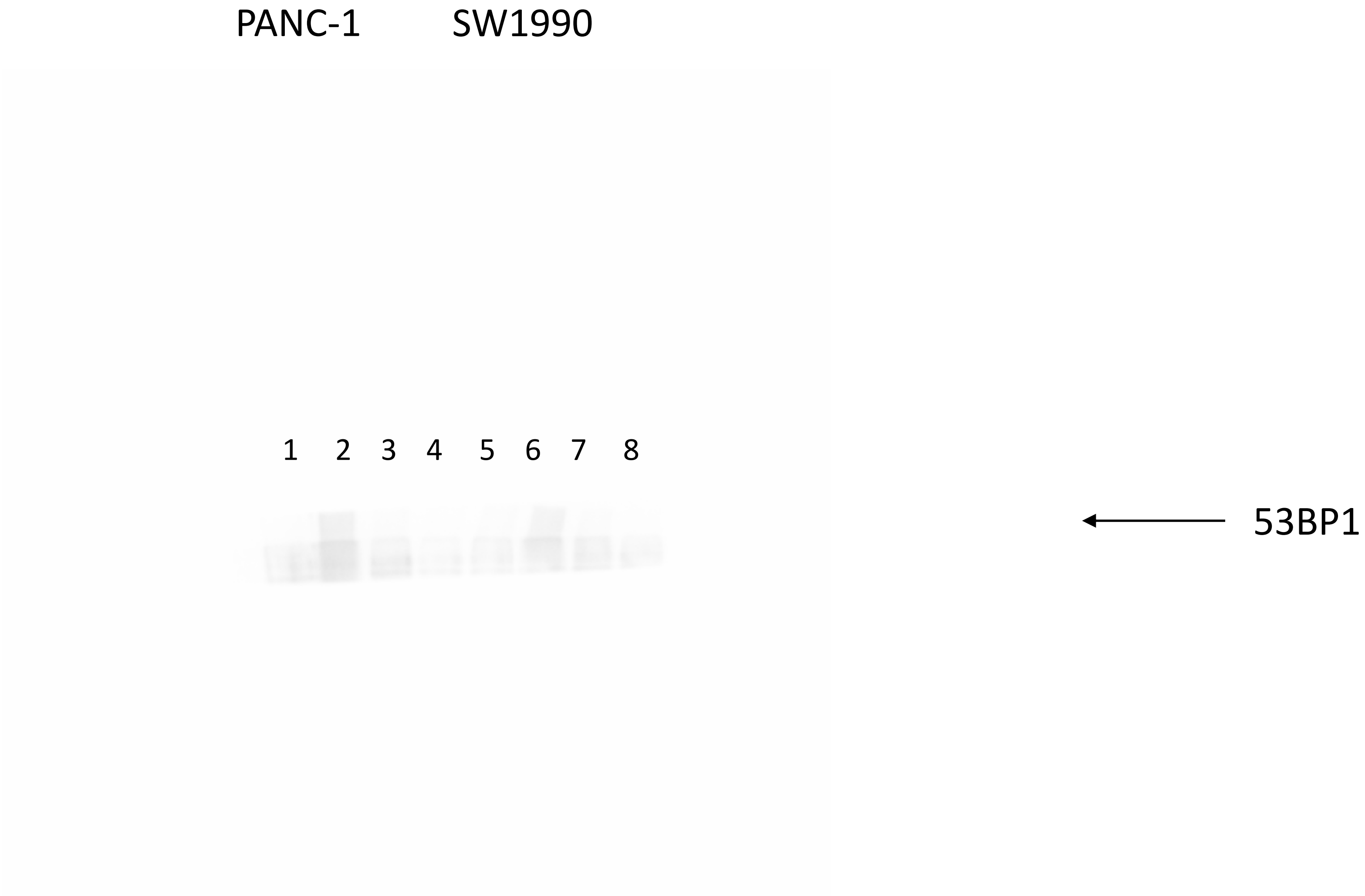

Full and uncropped western blot for Figure 4H

Lanes 1-8 are on the figure

PANC-1 SW1990

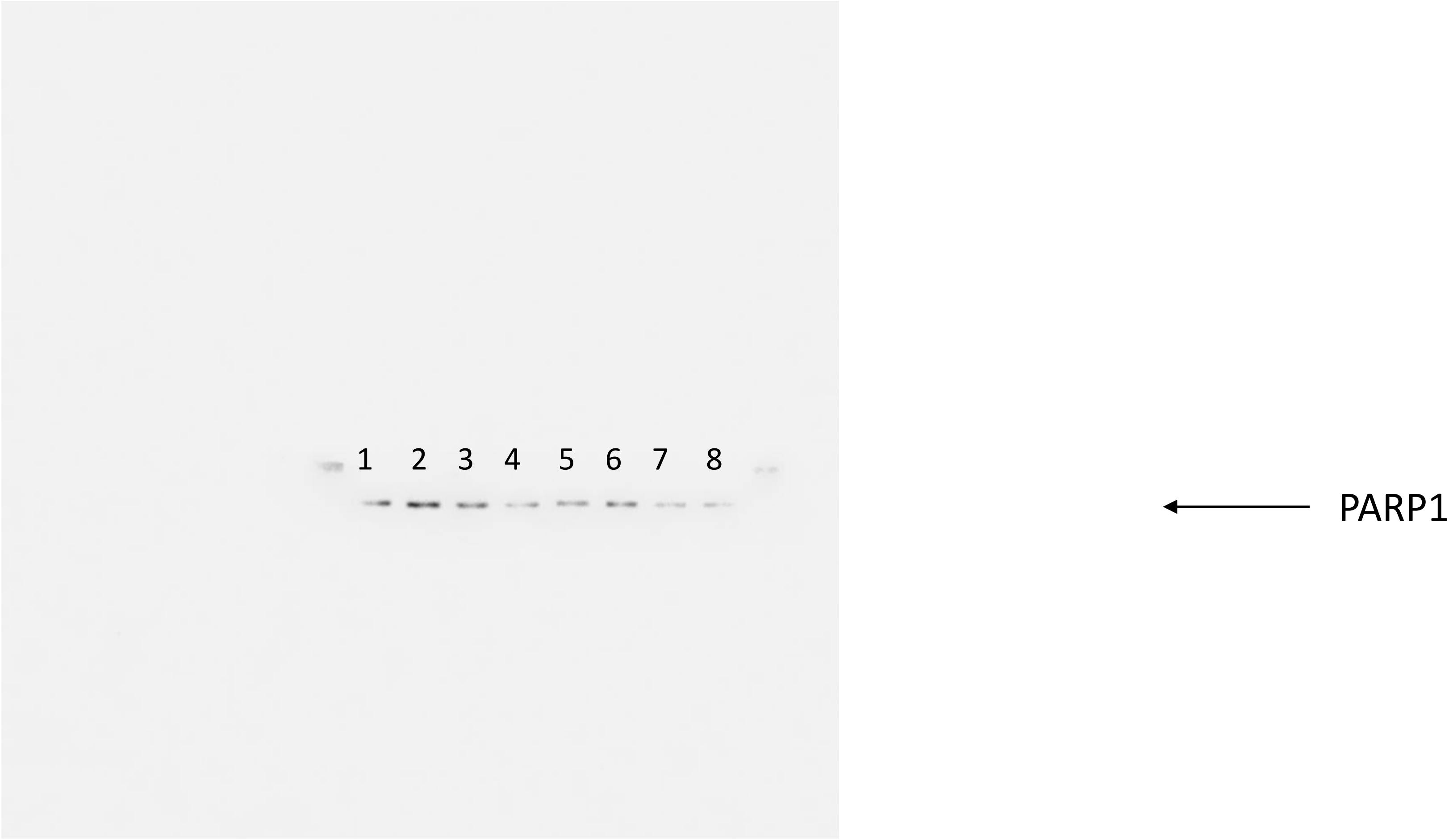

Full and uncropped western blot for Figure 4H

Lanes 1-8 are on the figure

PANC-1 SW1990

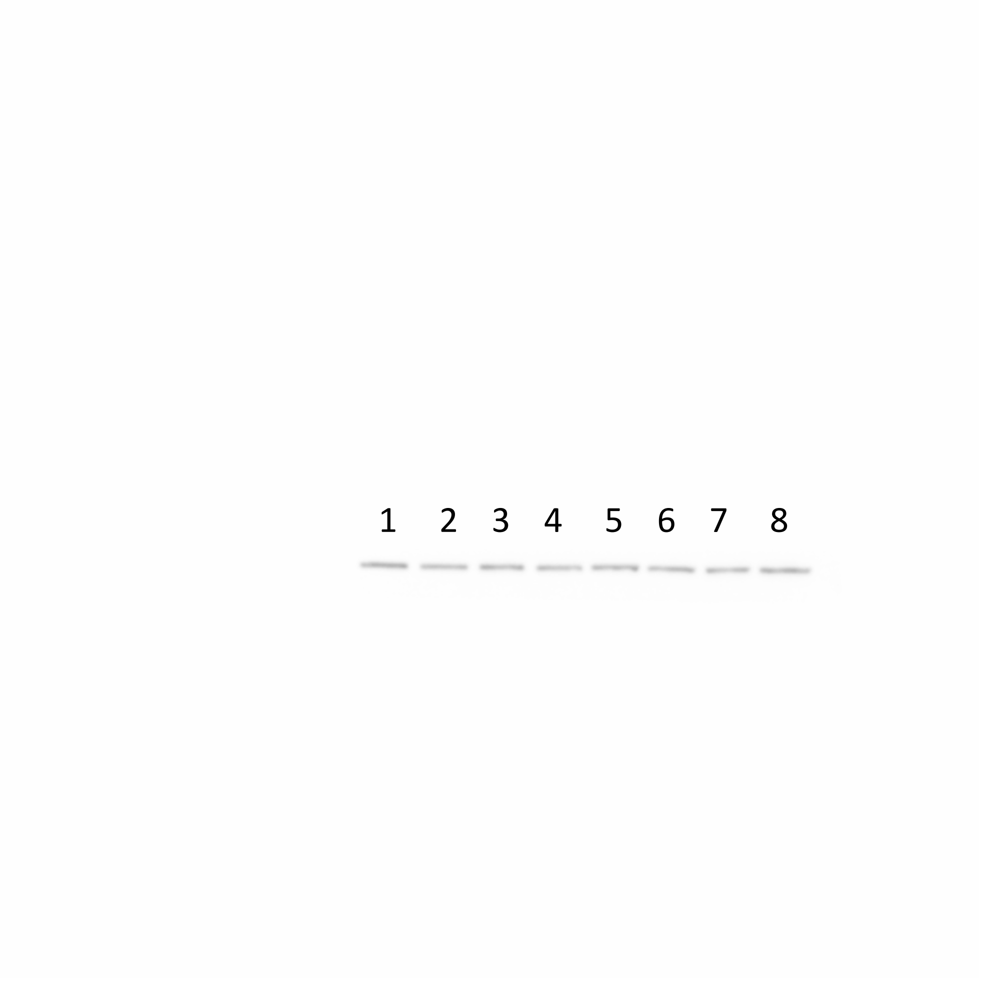

GAPDH

Full and uncropped western blot for Figure 4I

Lanes 1-8 are on the figure

PANC-1 SW1990

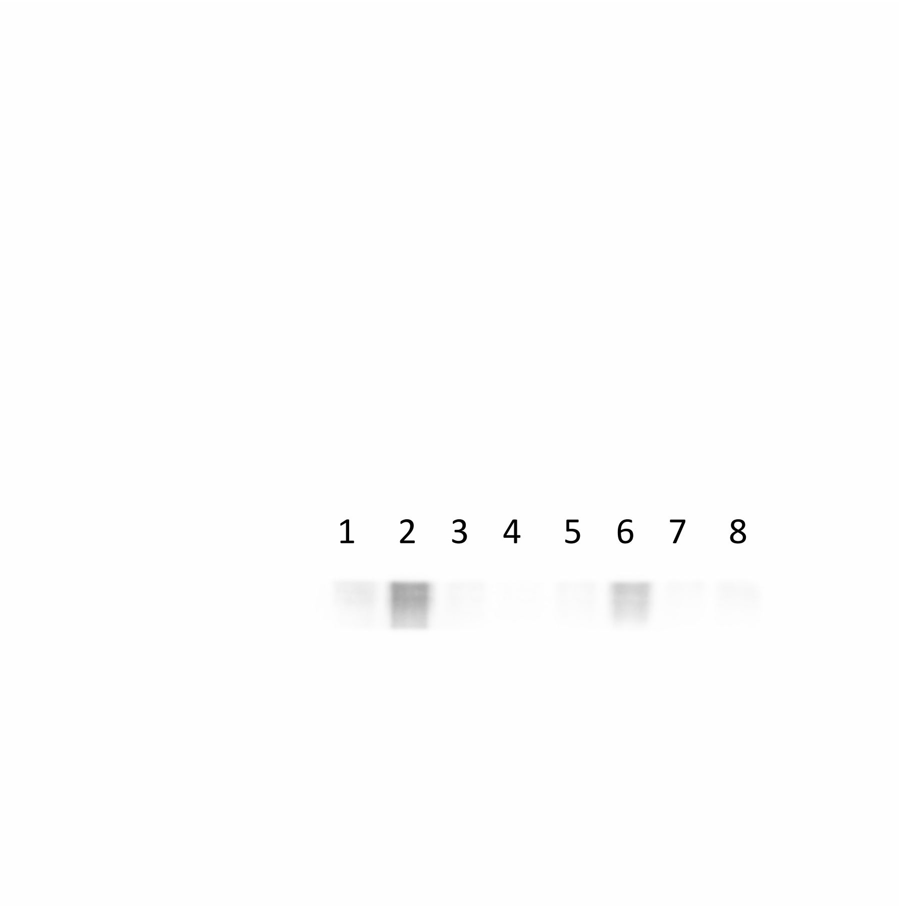

P-ATR

Full and uncropped western blot for Figure 4I

Lanes 1-8 are on the figure

PANC-1 SW1990

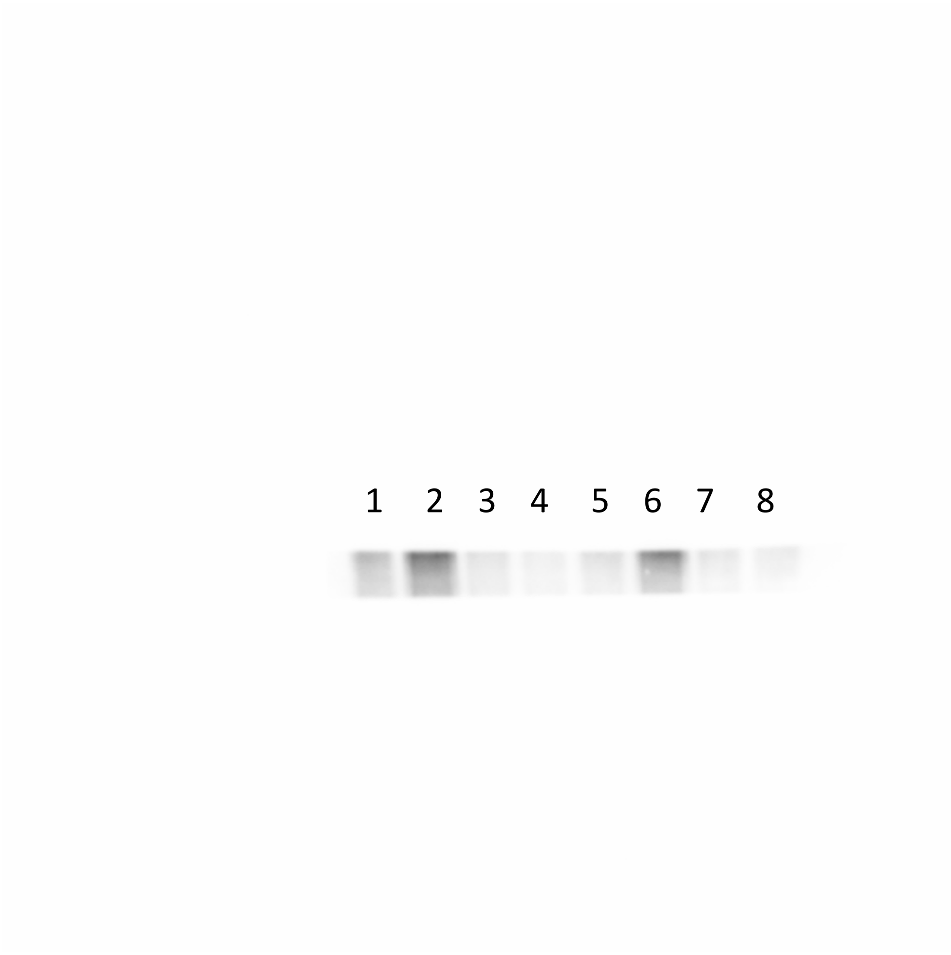

ATR

Full and uncropped western blot for Figure 4I

Lanes 1-8 are on the figure

PANC-1 SW1990

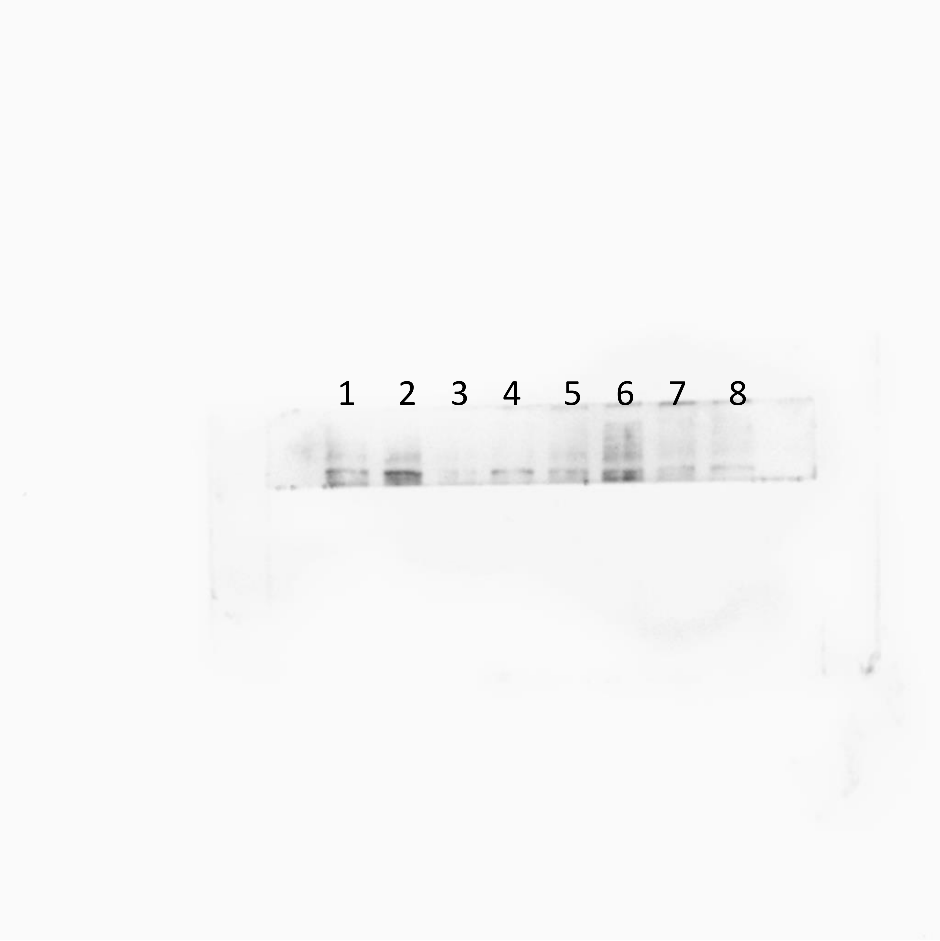

P-ATM

Full and uncropped western blot for Figure 4I

Lanes 1-8 are on the figure

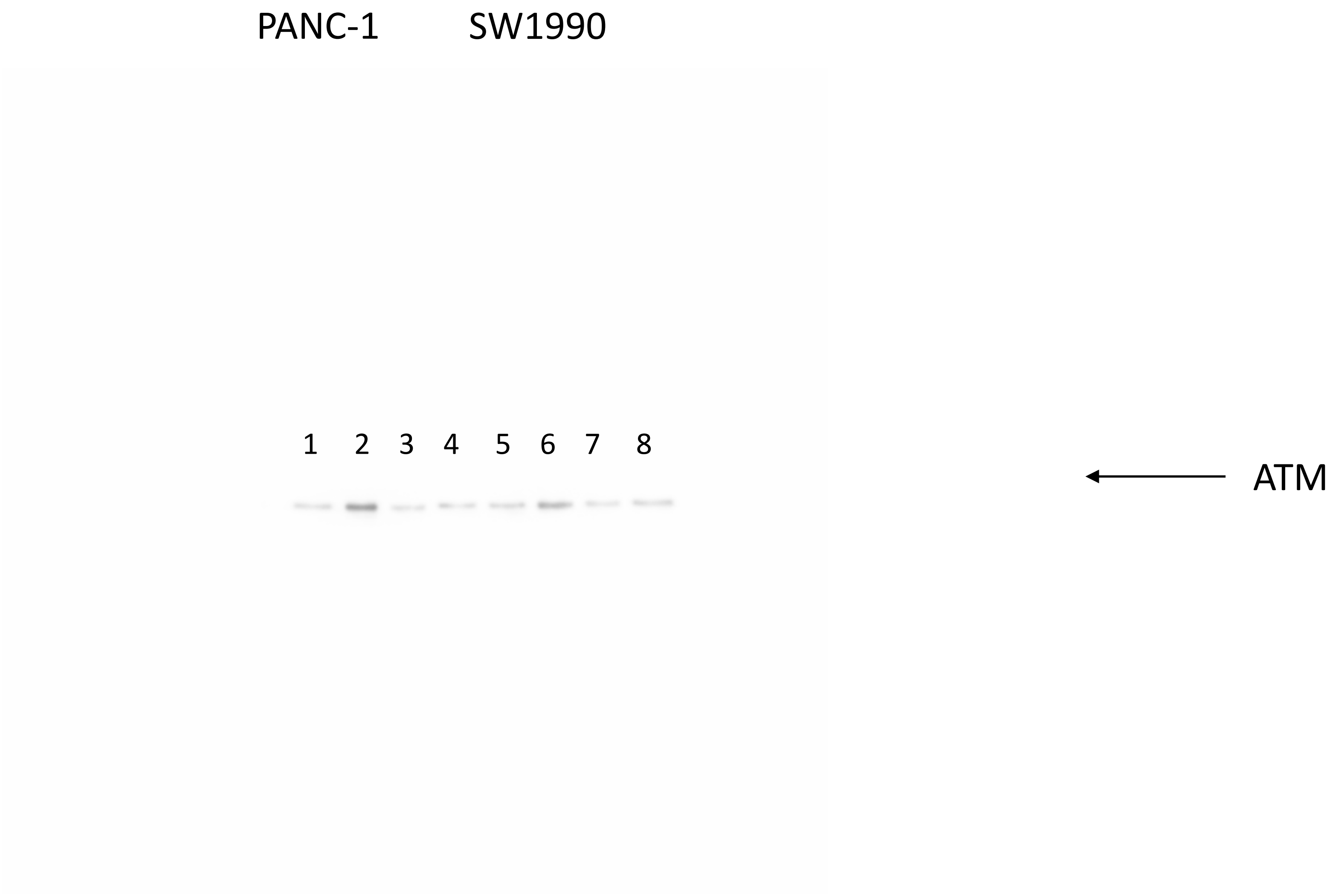

Full and uncropped western blot for Figure 4I

Lanes 1-8 are on the figure

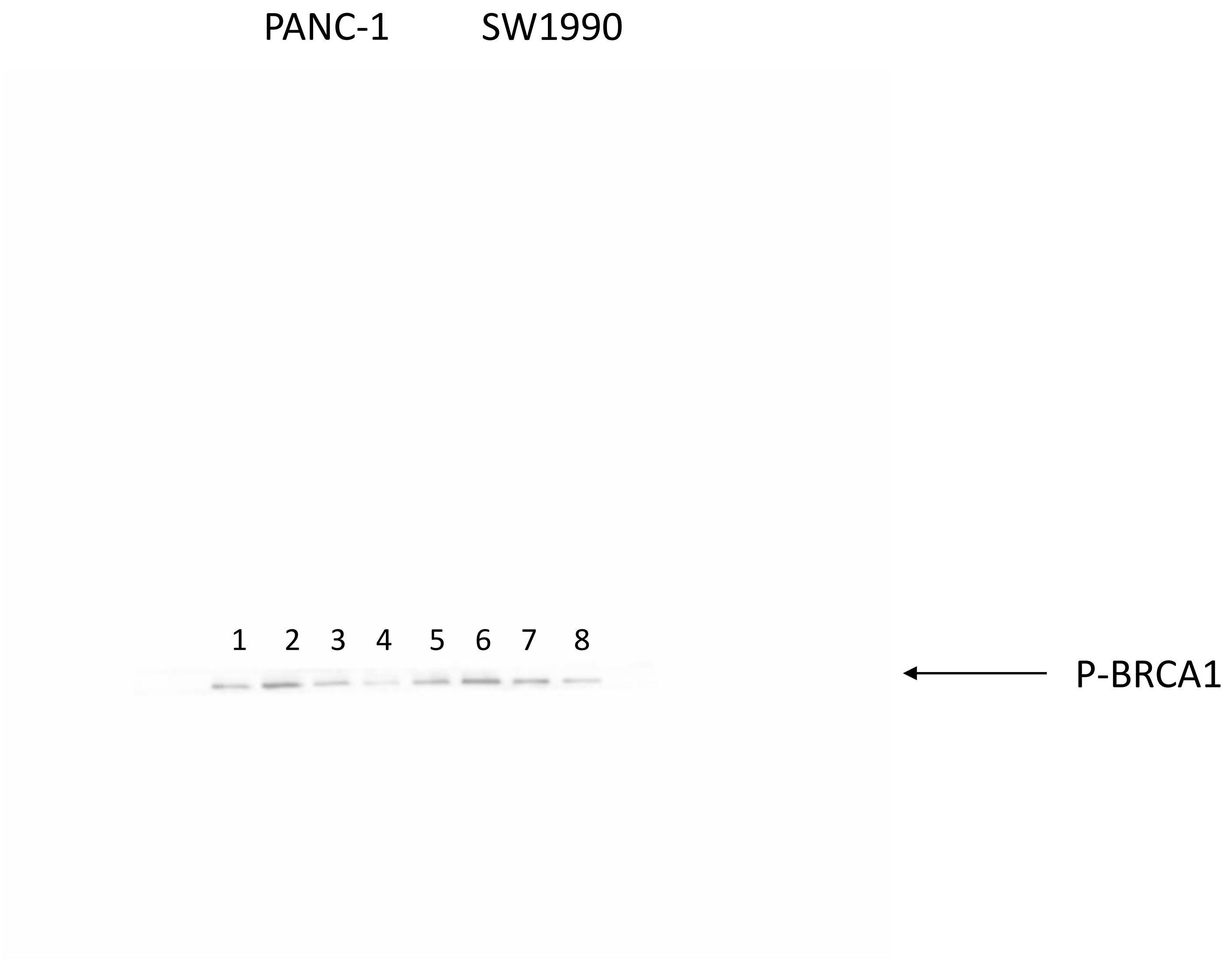

Full and uncropped western blot for Figure 4I

Lanes 1-8 are on the figure

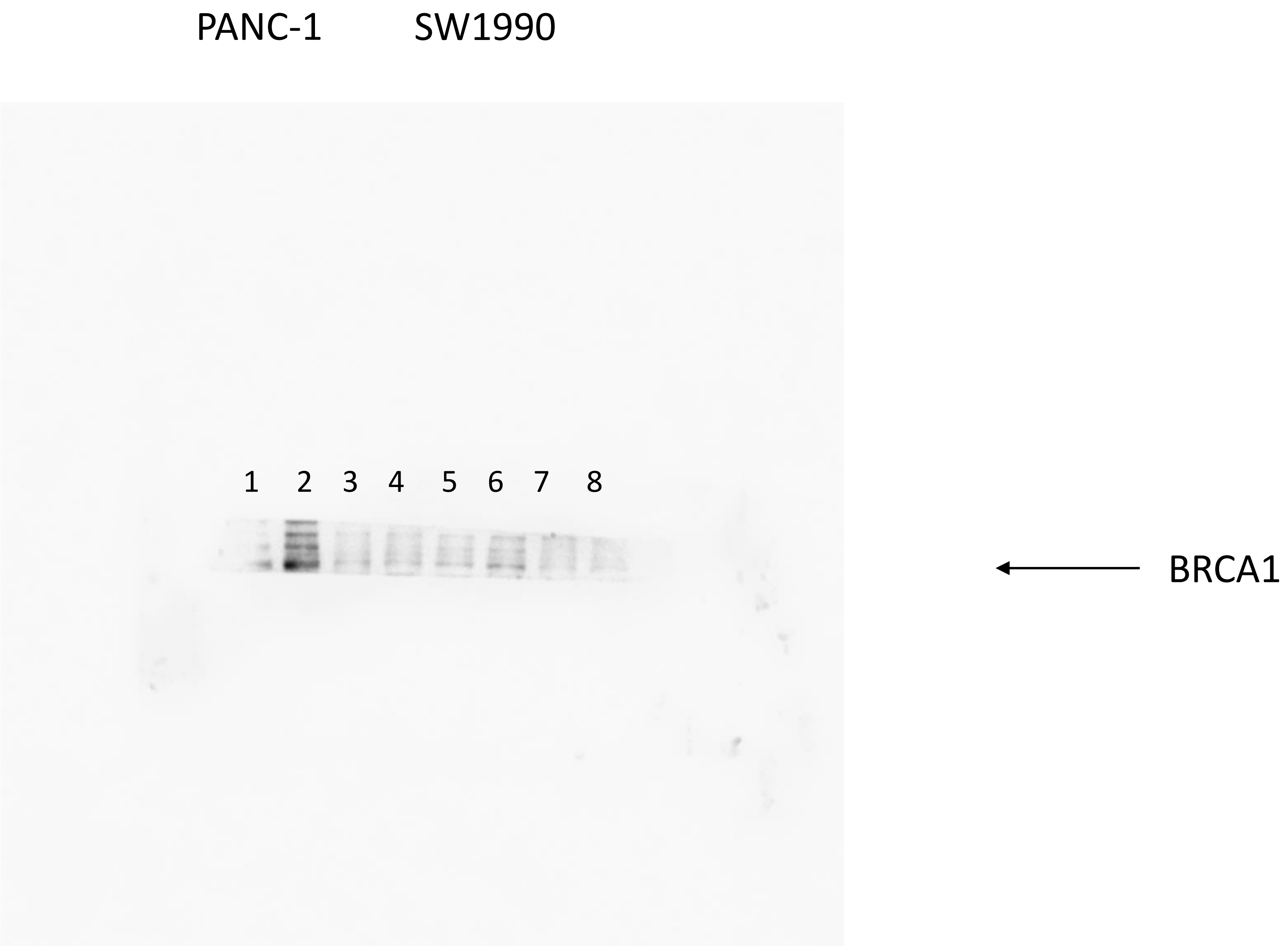

Full and uncropped western blot for Figure 4I

Lanes 1-8 are on the figure

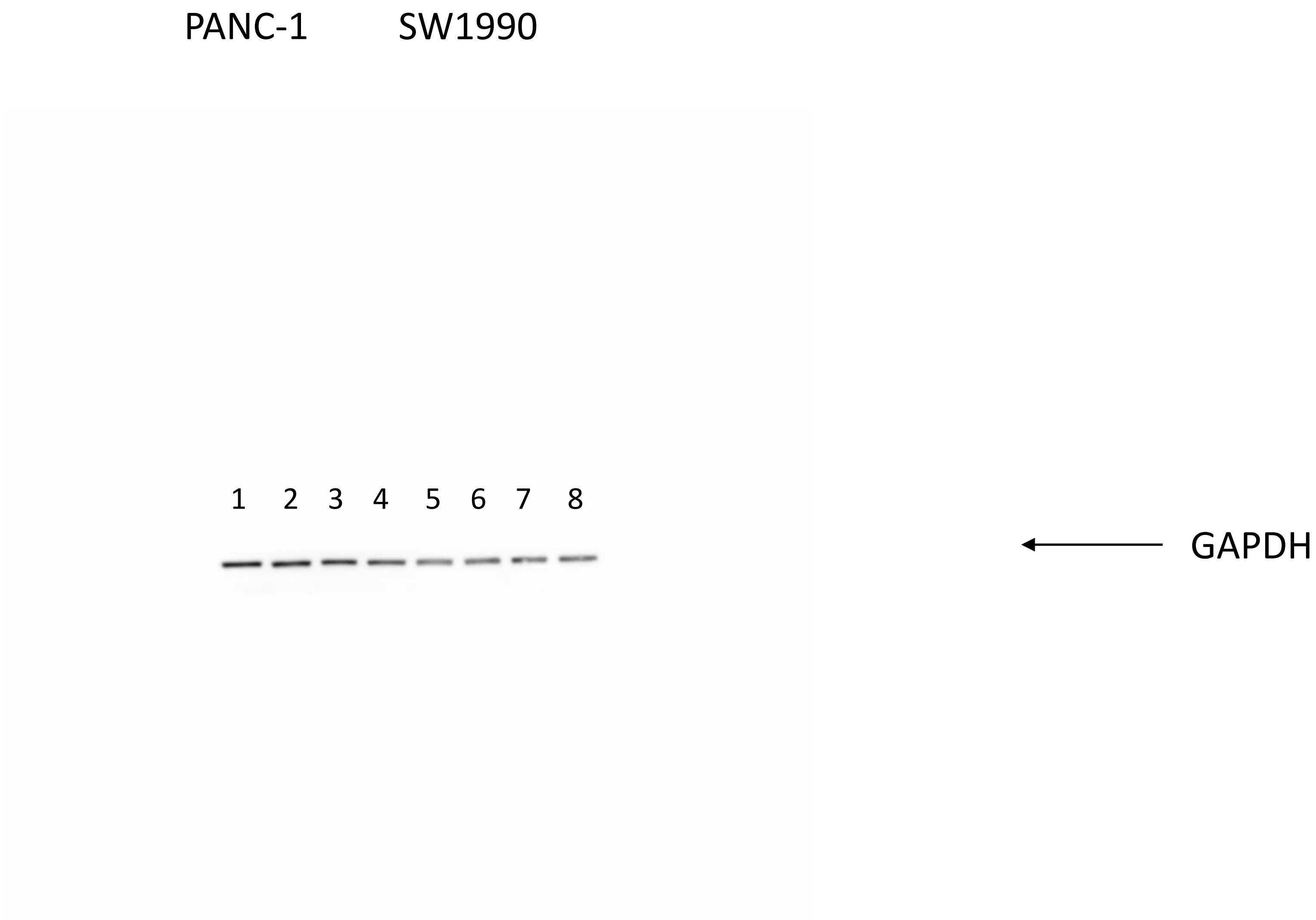

Full and uncropped western blot for Figure 4I

Lanes 1-8 are on the figure

PANC-1 SW1990

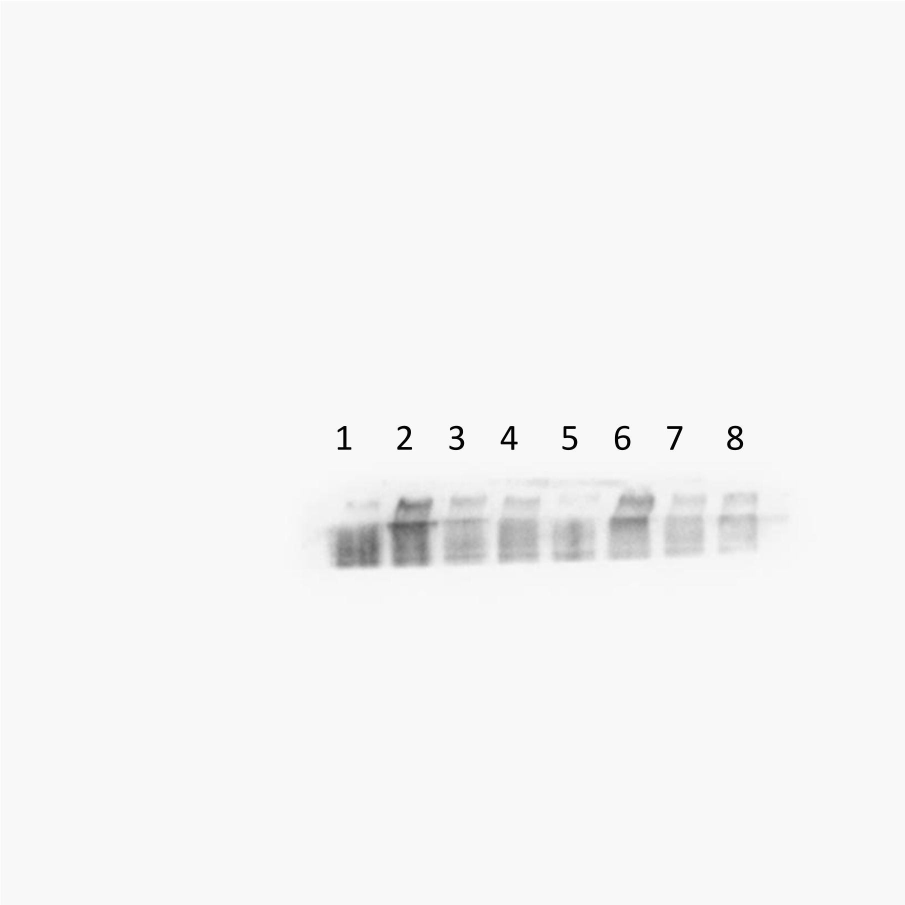

← P-ATR

Full and uncropped western blot for Figure 4I

Lanes 1-8 are on the figure

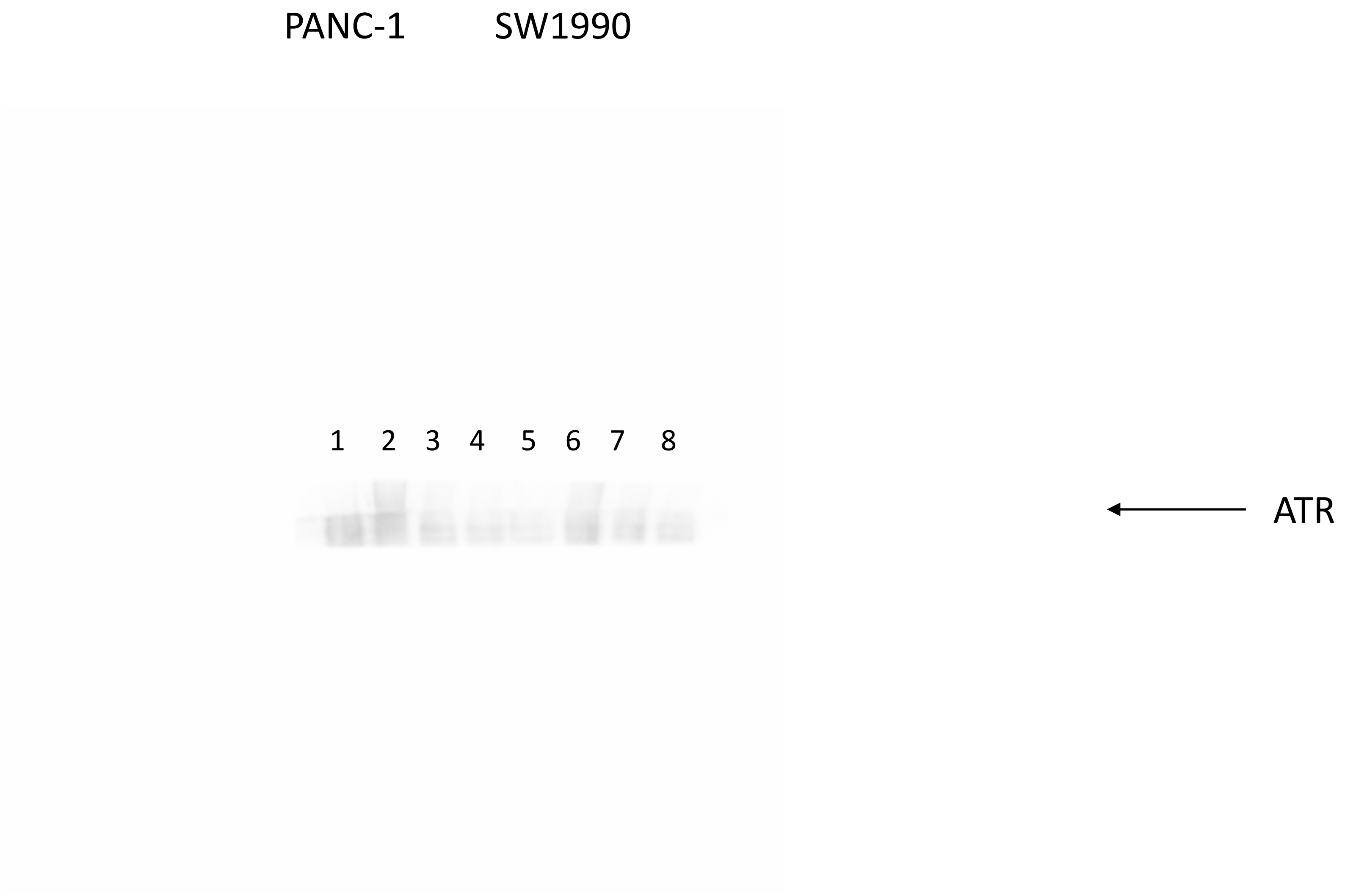

Full and uncropped western blot for Figure 4I

Lanes 1-8 are on the figure

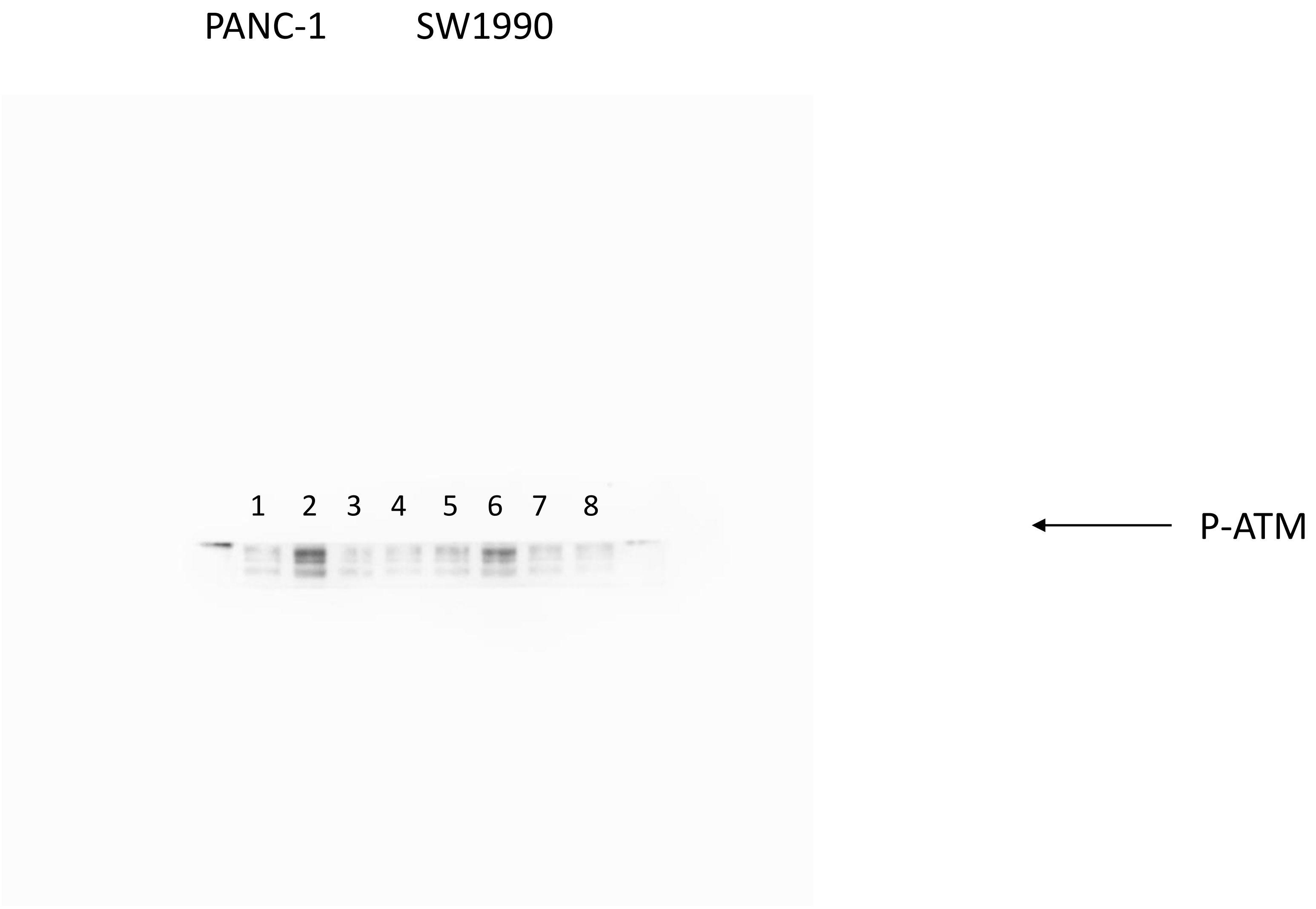

Full and uncropped western blot for Figure 4I

Lanes 1-8 are on the figure

PANC-1 SW1990

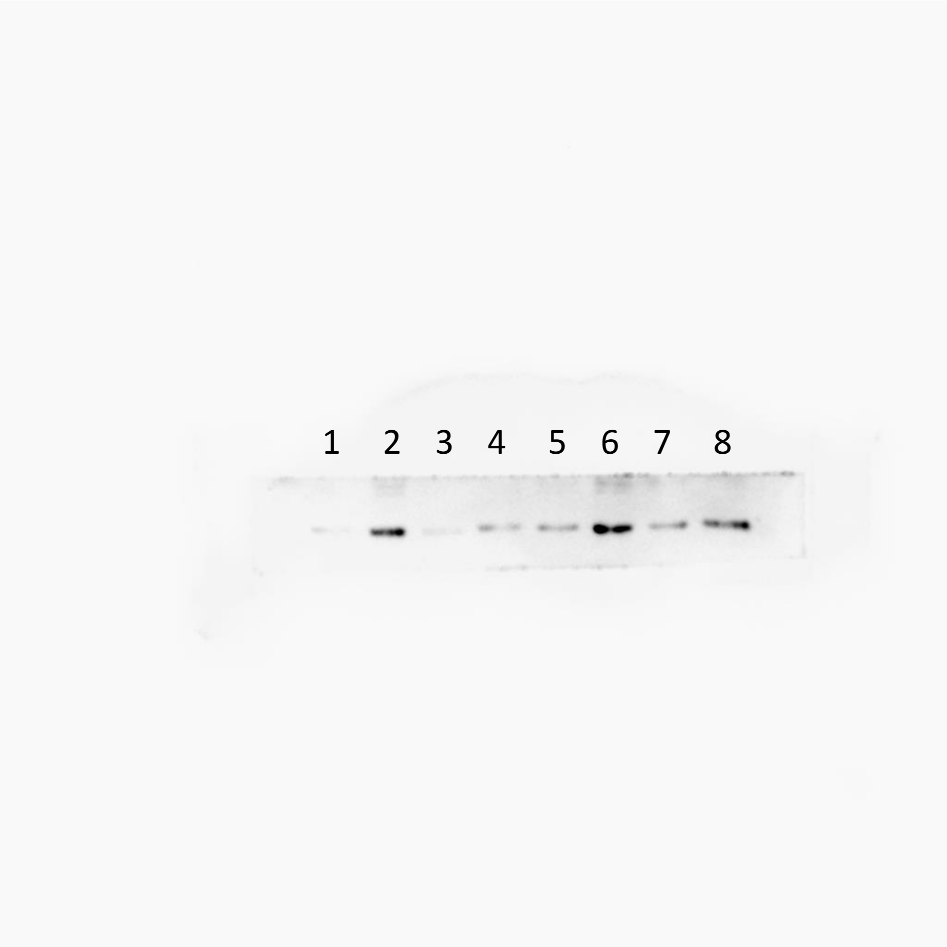

ATM

Full and uncropped western blot for Figure 4I

Lanes 1-8 are on the figure

PANC-1 SW1990

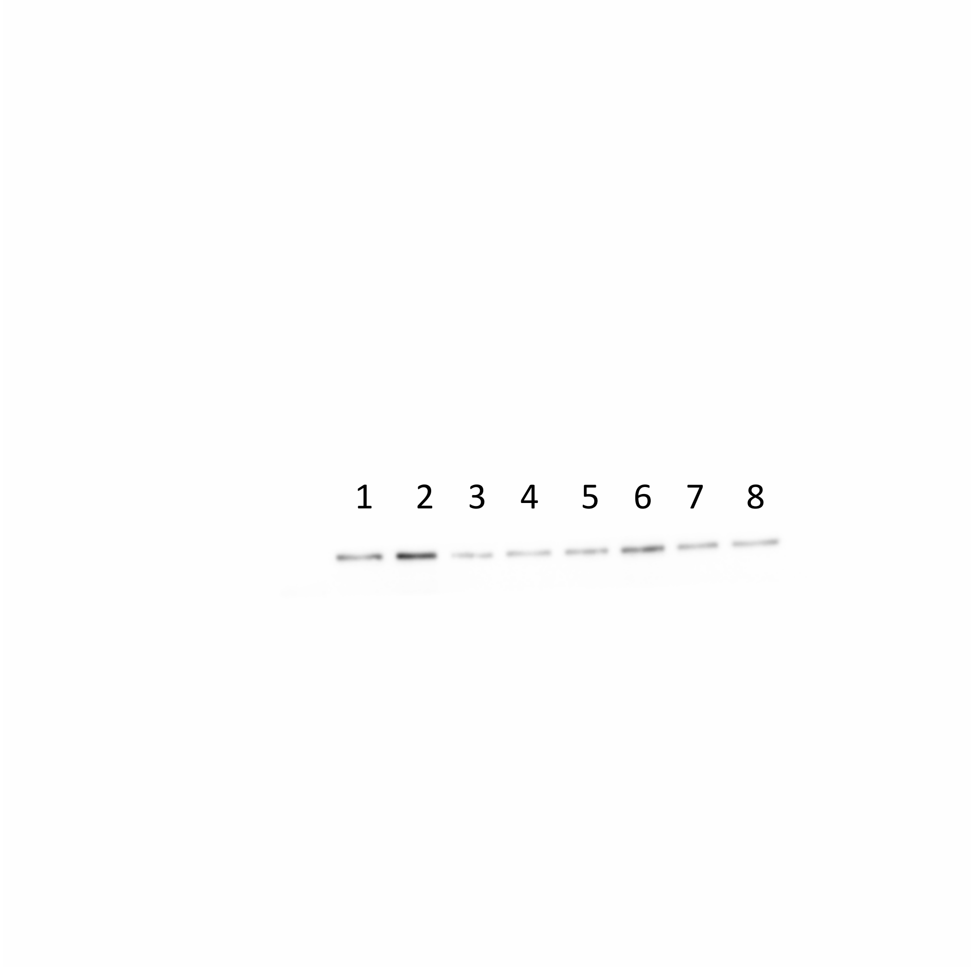

P-BRCA1

Full and uncropped western blot for Figure 4I

Lanes 1-8 are on the figure

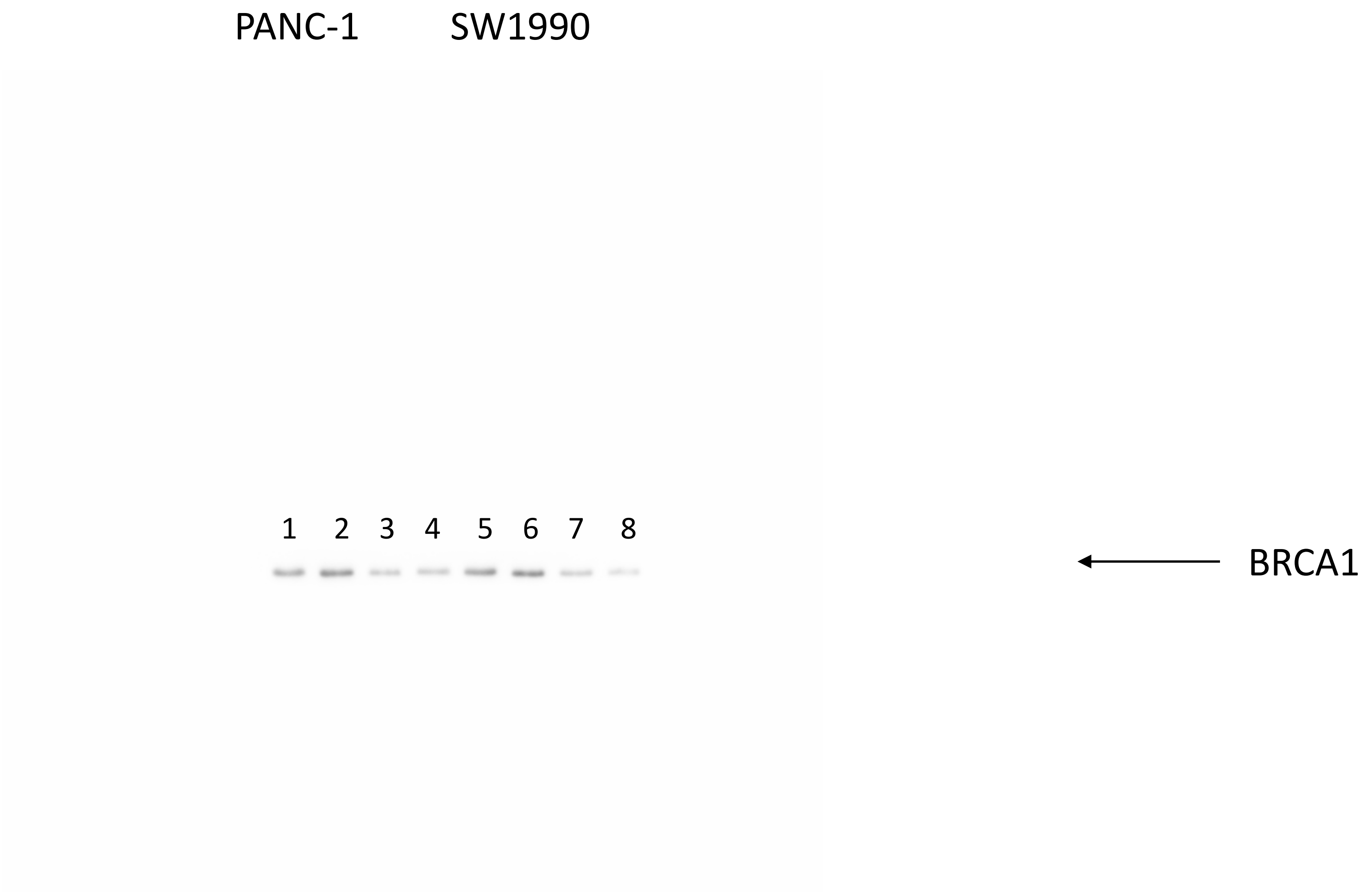

Full and uncropped western blot for Figure 4I

Lanes 1-8 are on the figure

PANC-1 SW1990

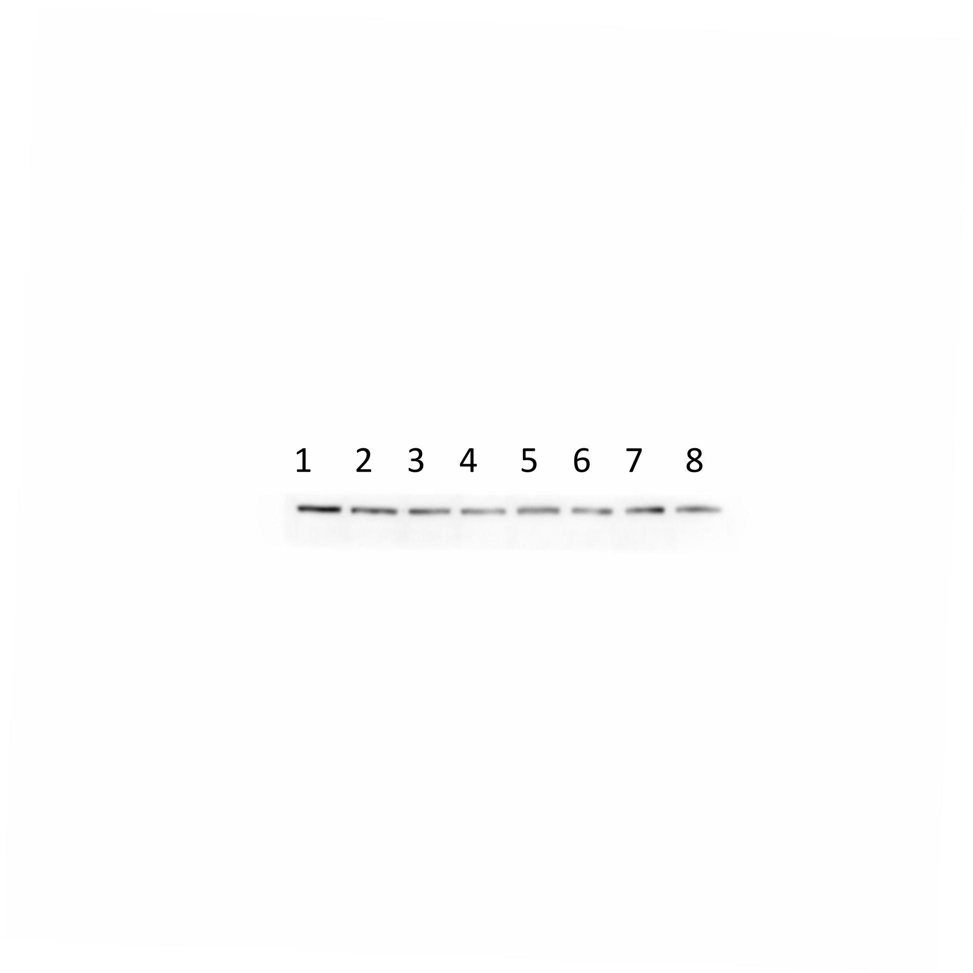

GAPDH

Full and uncropped western blot for Figure 4J

Lanes 1-8 are on the figure

PANC-1 SW1990

1 2 3 4 5 6 7 8

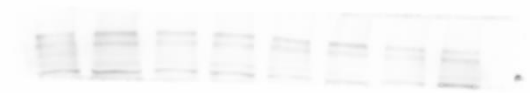

P-ATR

Full and uncropped western blot for Figure 4J

Lanes 1-8 are on the figure

PANC-1 SW1990

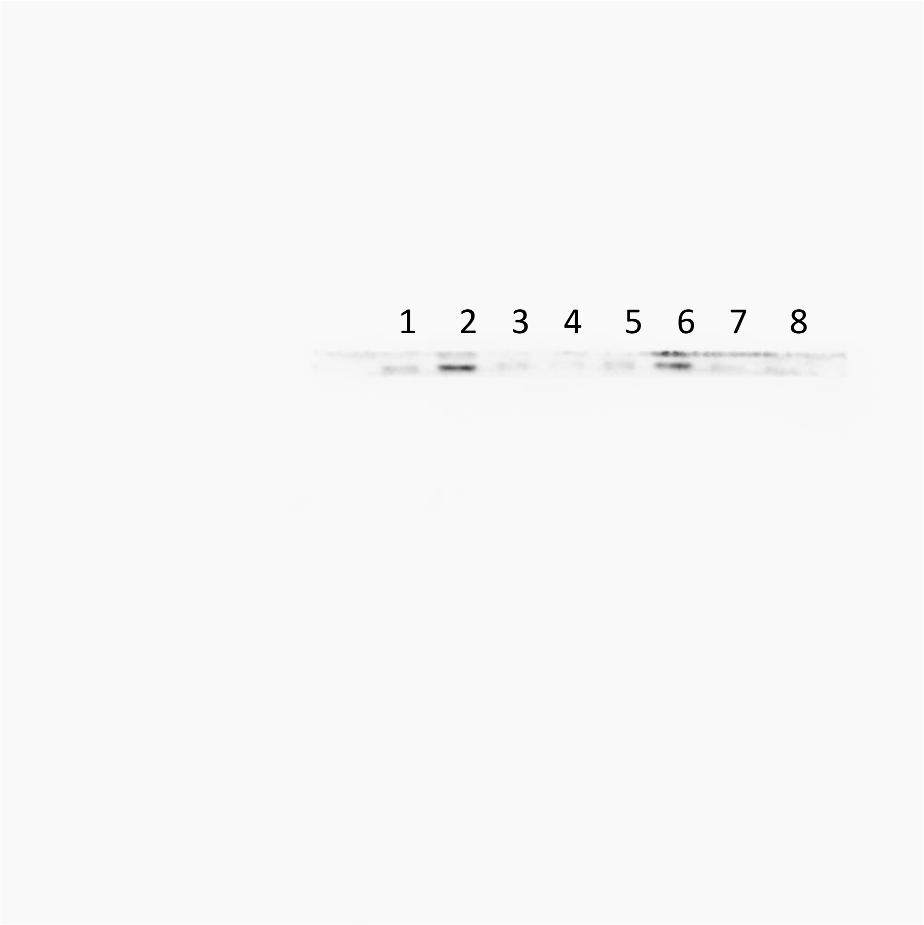

ATR

Full and uncropped western blot for Figure 4J

Lanes 1-8 are on the figure

PANC-1 SW1990

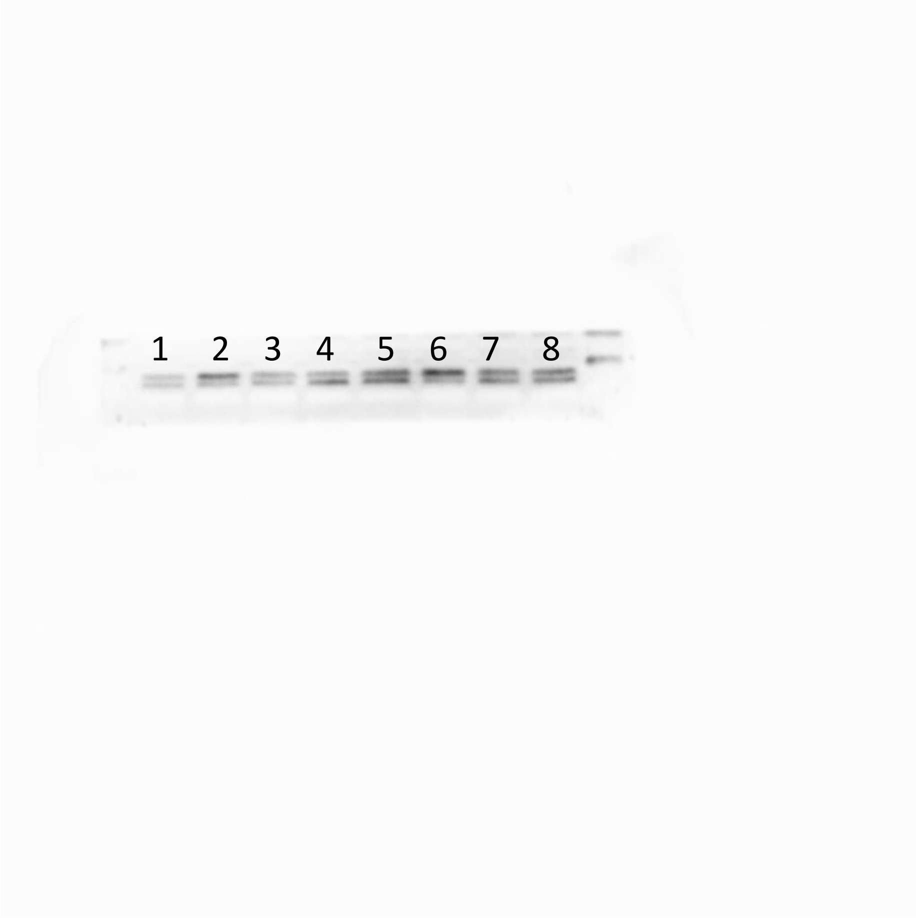

P-CHK1

Full and uncropped western blot for Figure 4J

Lanes 1-8 are on the figure

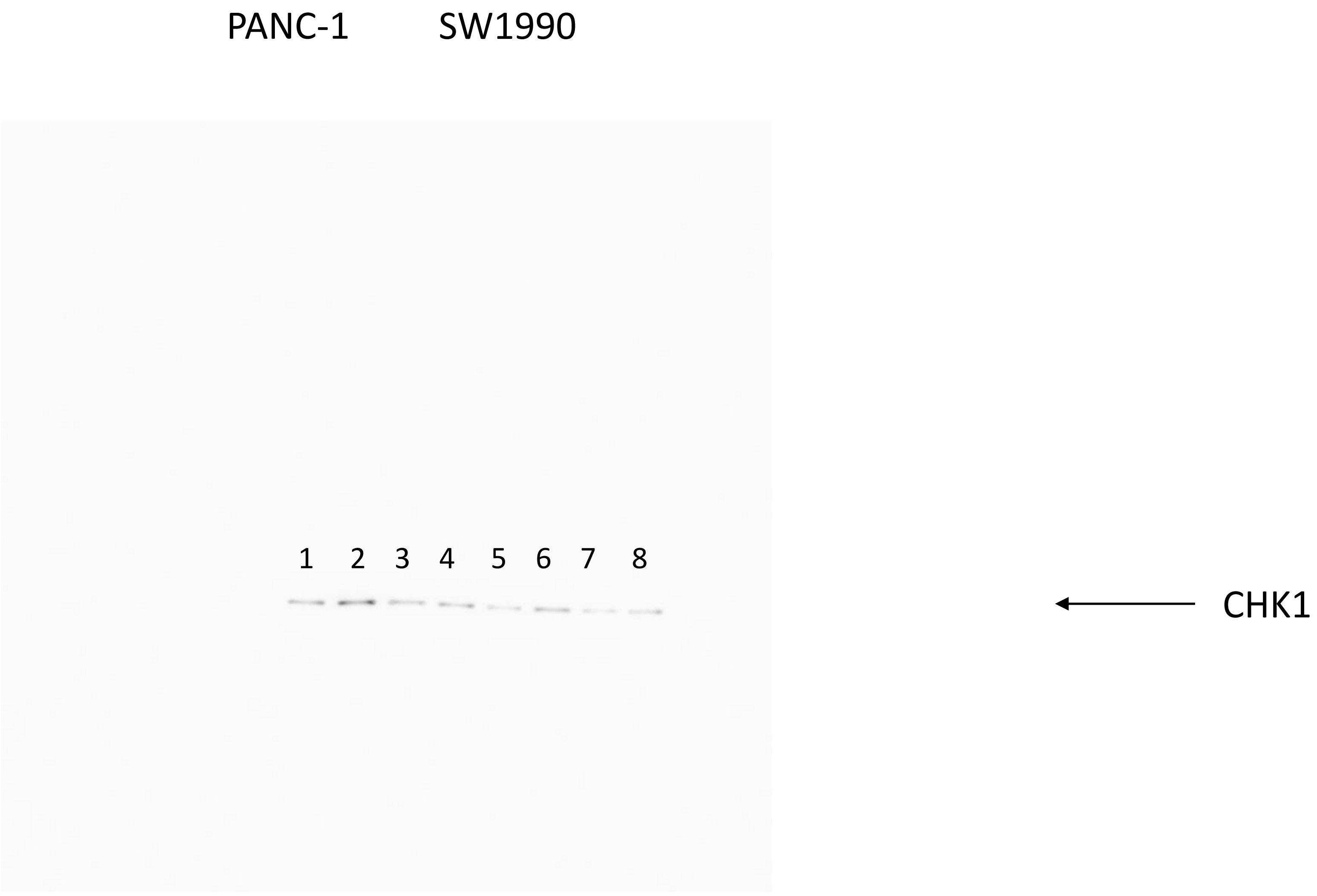

Full and uncropped western blot for Figure 4J

Lanes 1-8 are on the figure

PANC-1 SW1990

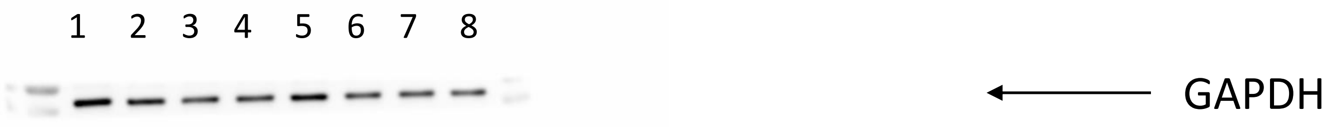

Full and uncropped western blot for Figure 4K

Lanes 1-8 are on the figure

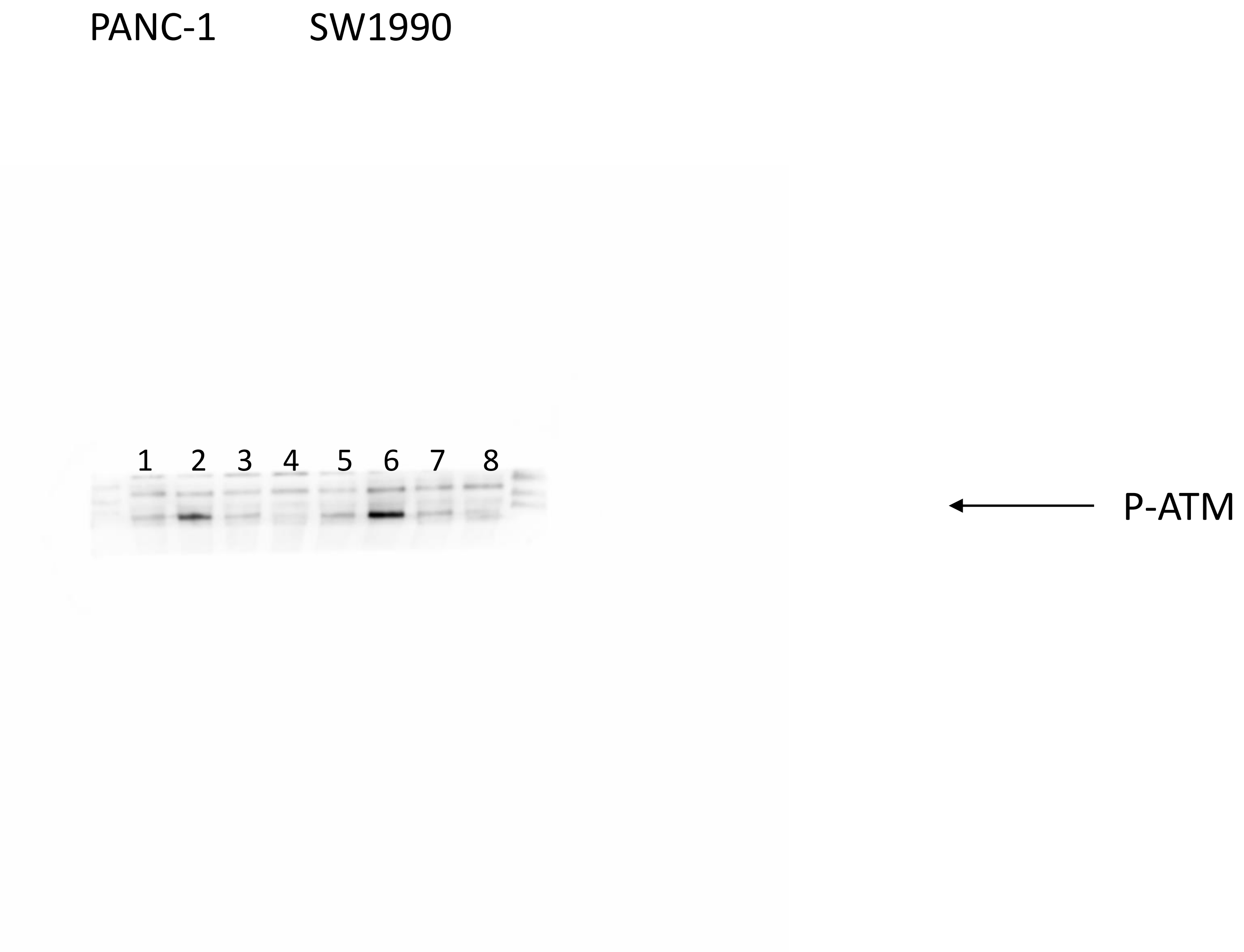

Full and uncropped western blot for Figure 4K

Lanes 1-8 are on the figure

PANC-1 SW1990

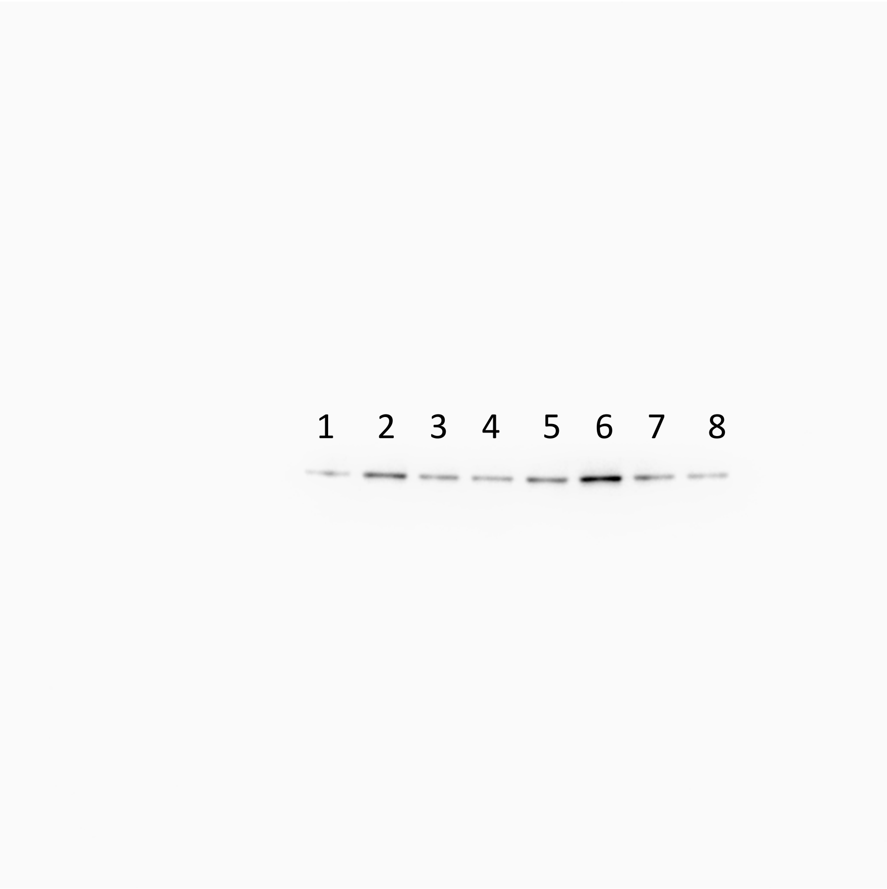

ATM

Full and uncropped western blot for Figure 4K

Lanes 1-8 are on the figure

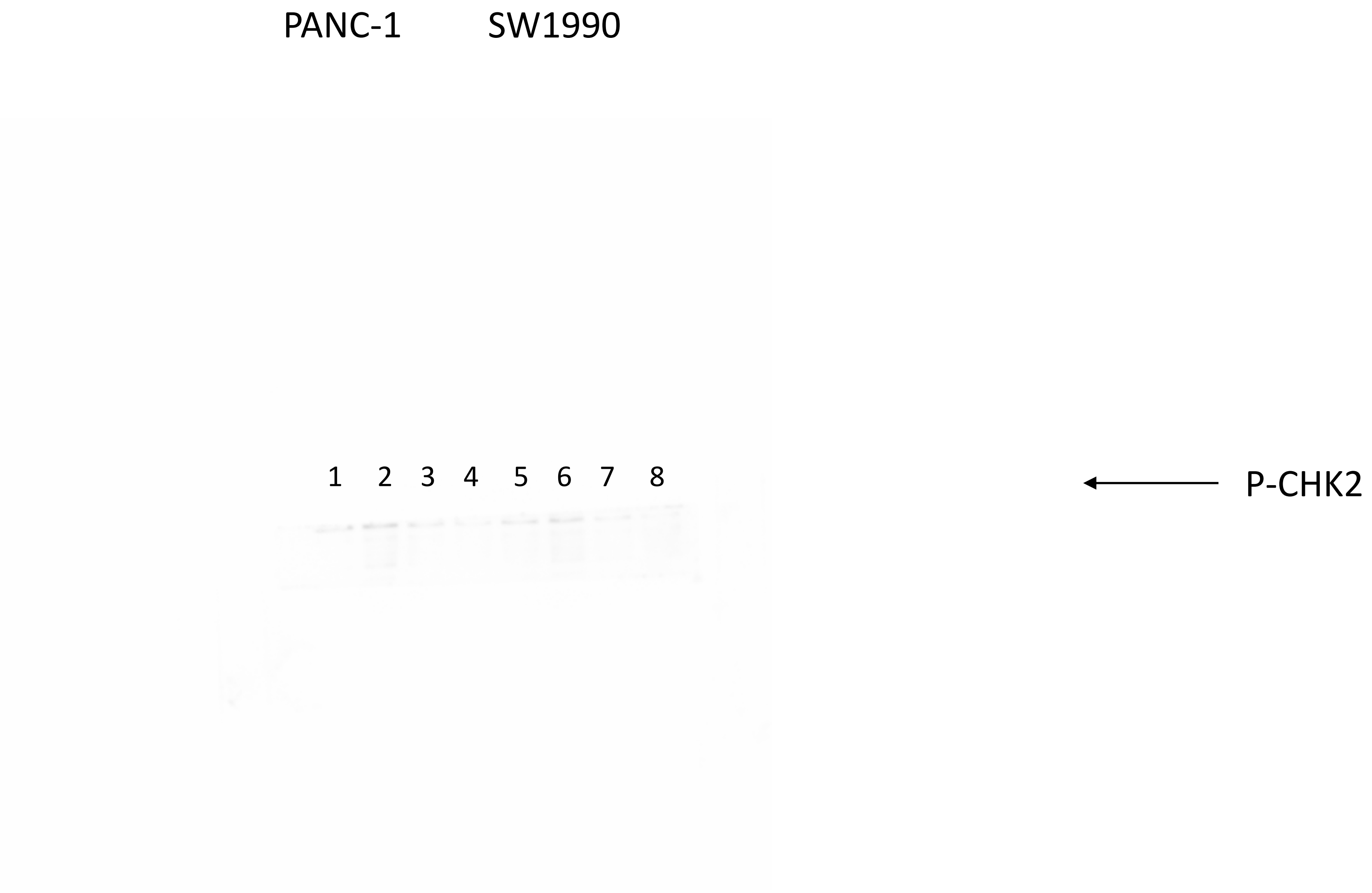

Full and uncropped western blot for Figure 4K

Lanes 1-8 are on the figure

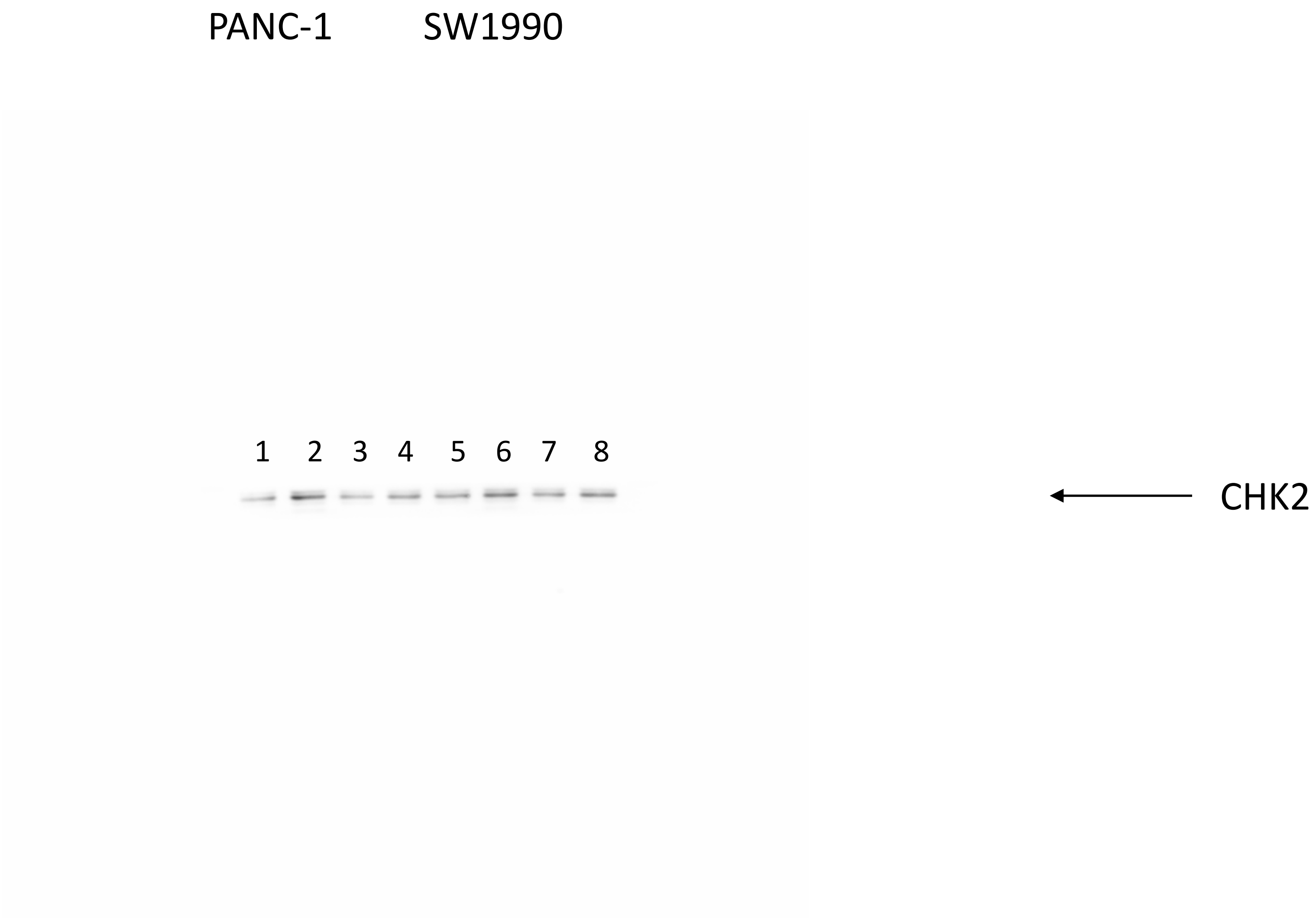

Full and uncropped western blot for Figure 4K

Lanes 1-8 are on the figure

PANC-1 SW1990

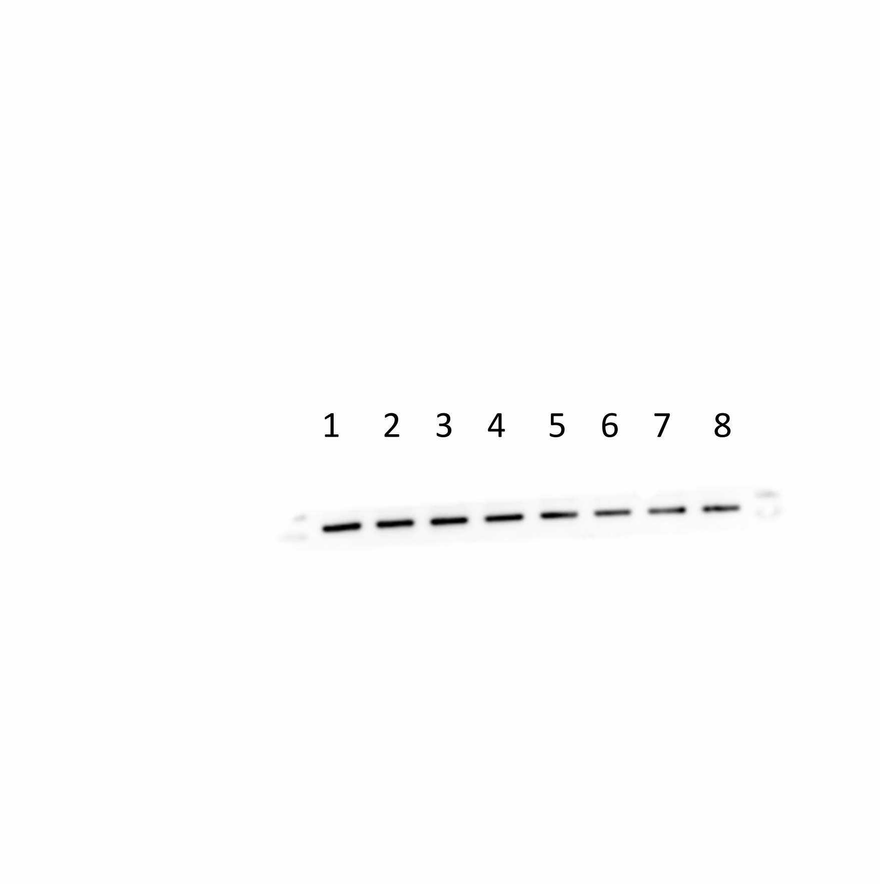

← GAPDH

Full and uncropped western blot for Figure 4L

Lanes 1-8 are on the figure

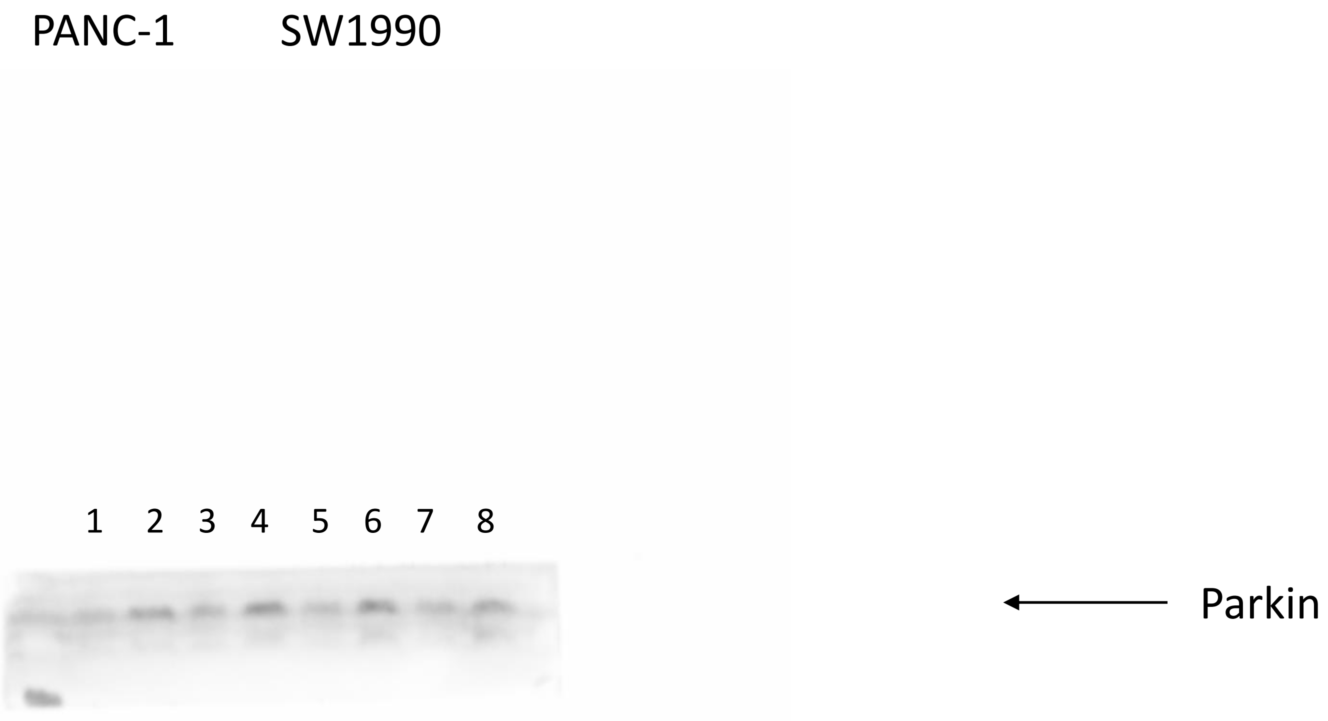

Full and uncropped western blot for Figure 4L

Lanes 1-8 are on the figure

PANC-1 SW1990

1 2 3 4 5 6 7 8

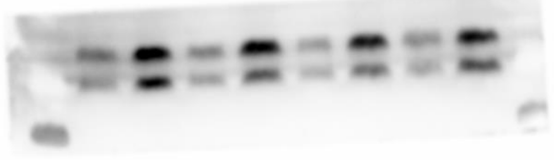

BNIP3

Full and uncropped western blot for Figure 4L

Lanes 1-8 are on the figure

PANC-1 SW1990

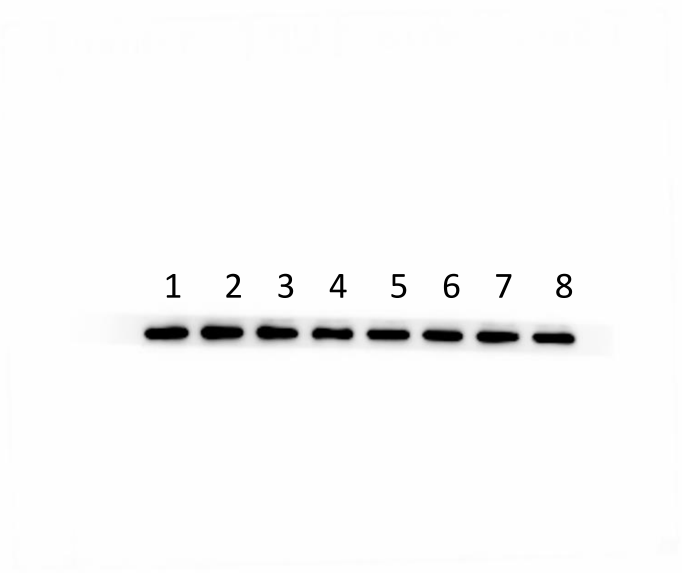

GAPDH

Full and uncropped western blot for Figure 4M

Lanes 1-8 are on the figure

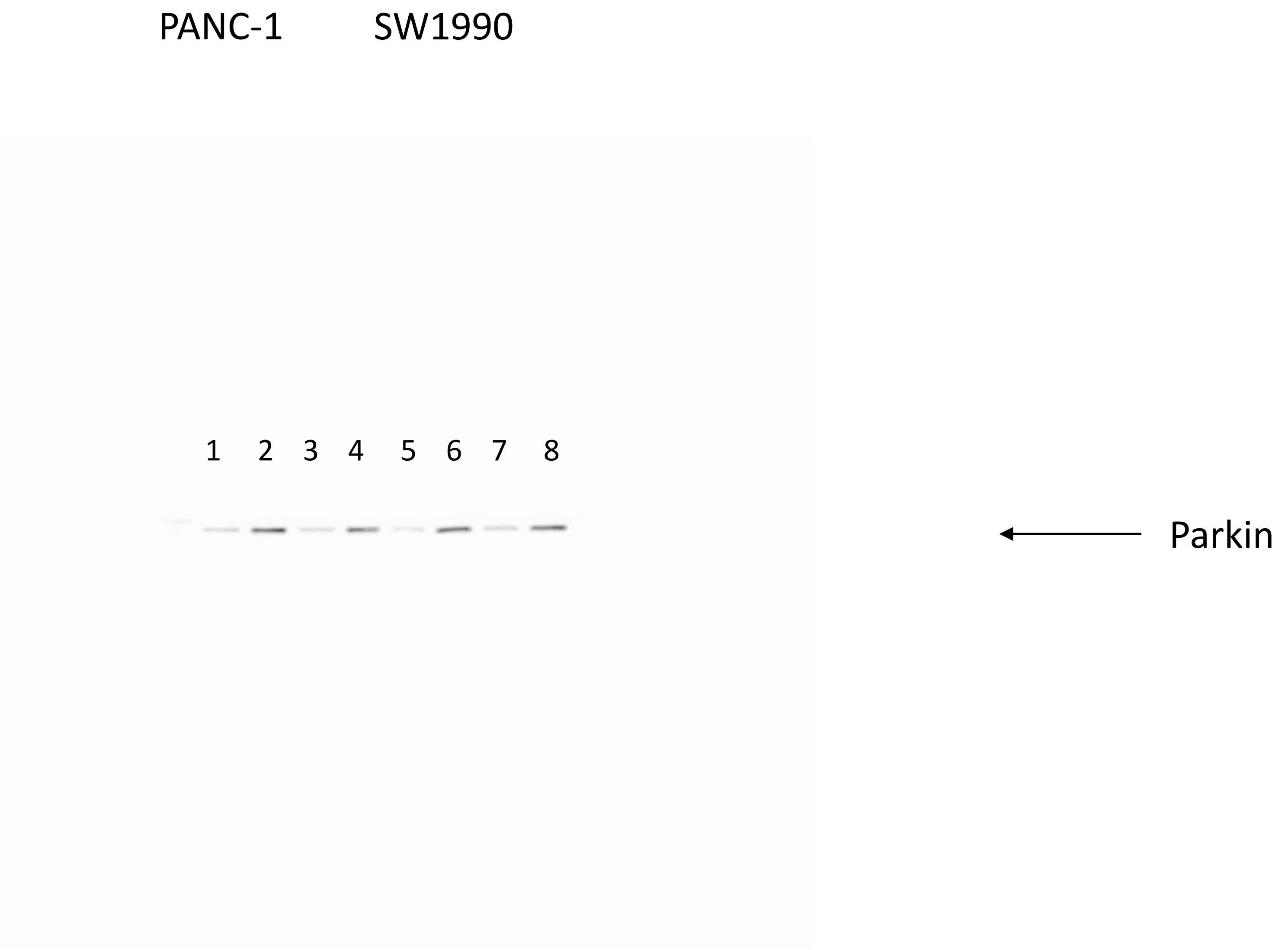

Full and uncropped western blot for Figure 4M

Lanes 1-8 are on the figure

PANC-1 SW1990

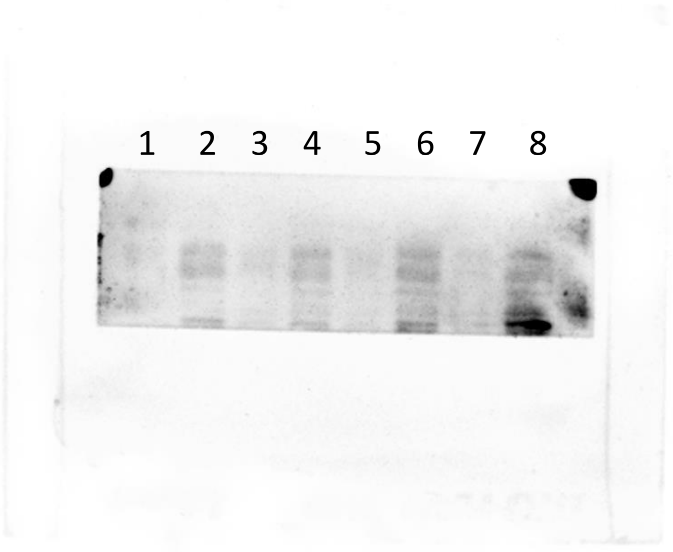

BNIP3

Full and uncropped western blot for Figure 4M

Lanes 1-8 are on the figure

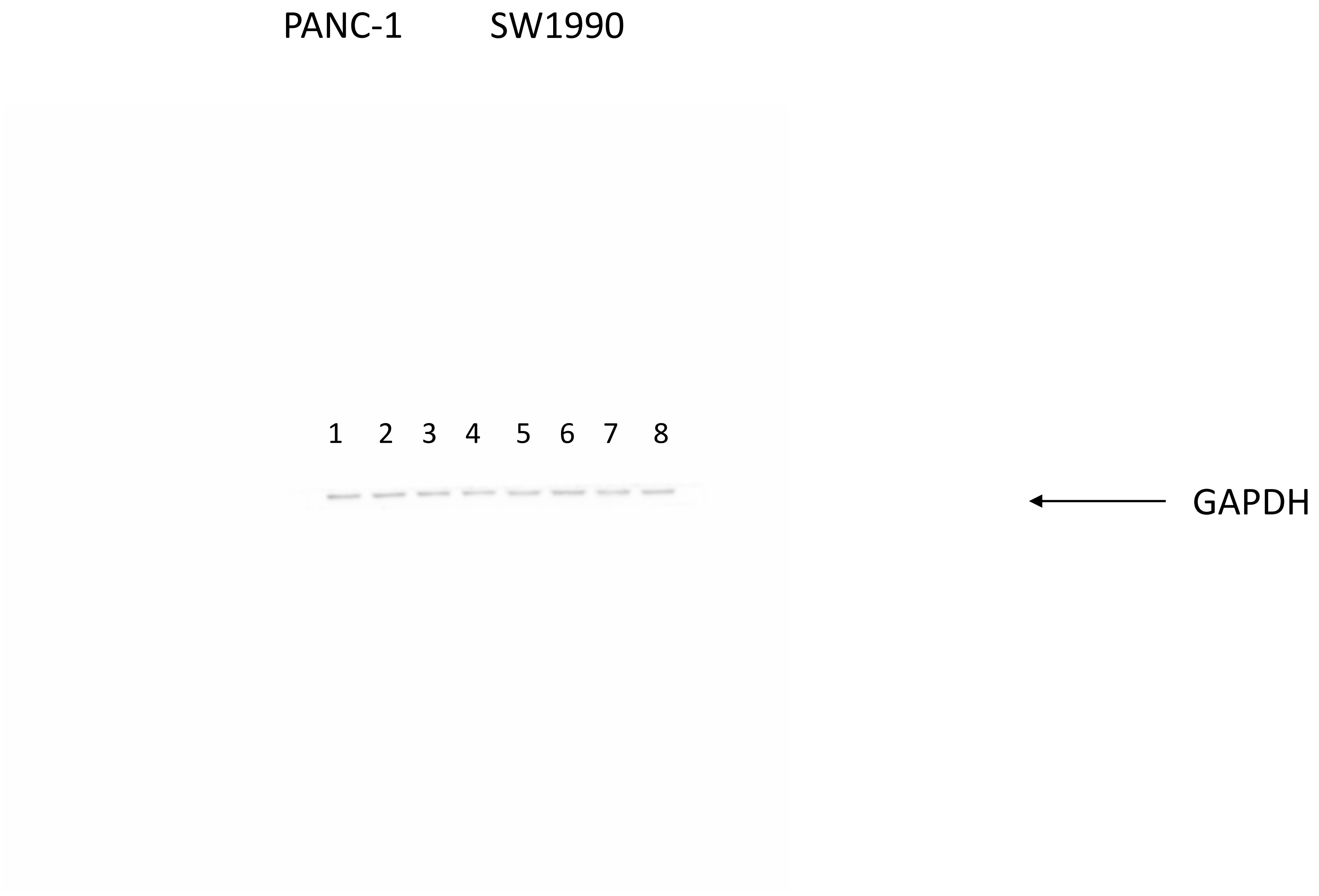

Full and uncropped western blot for Figure 5A

Lanes 1-8 are on the figure

B16 S91

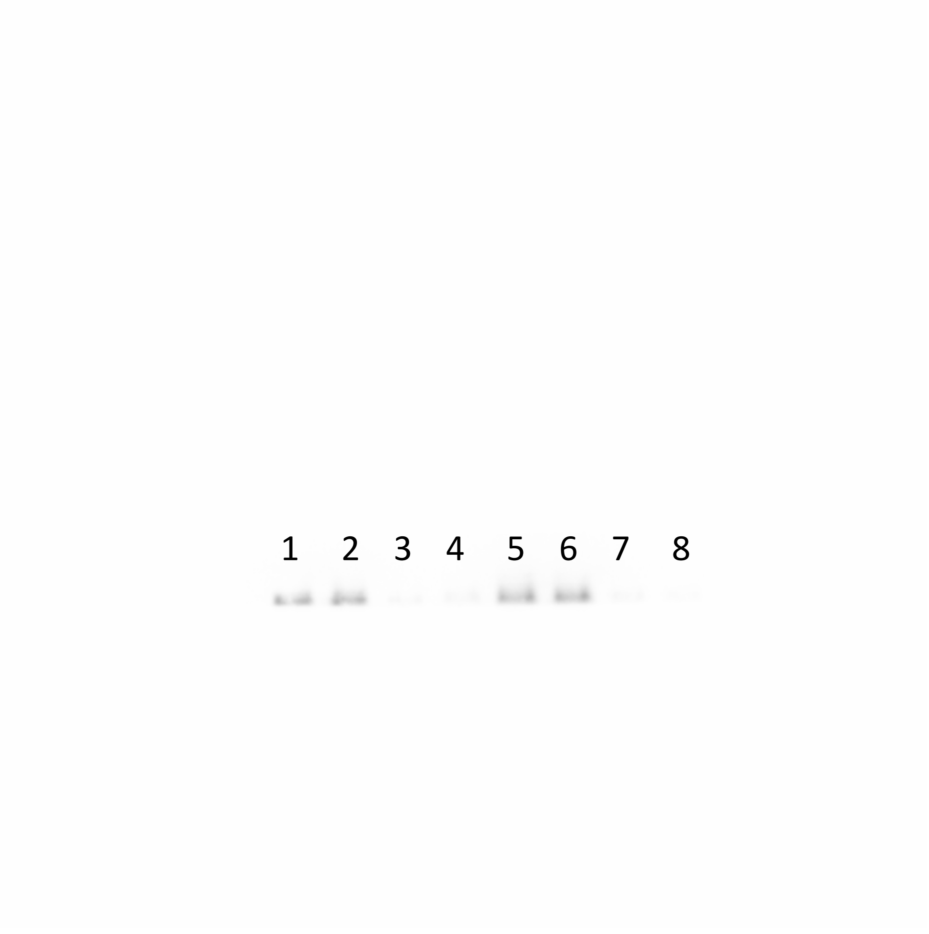

← Parkin

Full and uncropped western blot for Figure 5A

Lanes 1-8 are on the figure

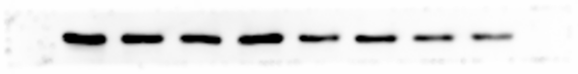

Full and uncropped western blot for Figure 5A

Lanes 1-8 are on the figure

B16 S91

1 2 3 4 5 6 7 8

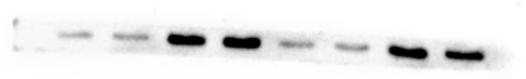

← Parkin

Full and uncropped western blot for Figure 5A

Lanes 1-8 are on the figure

B16 S91

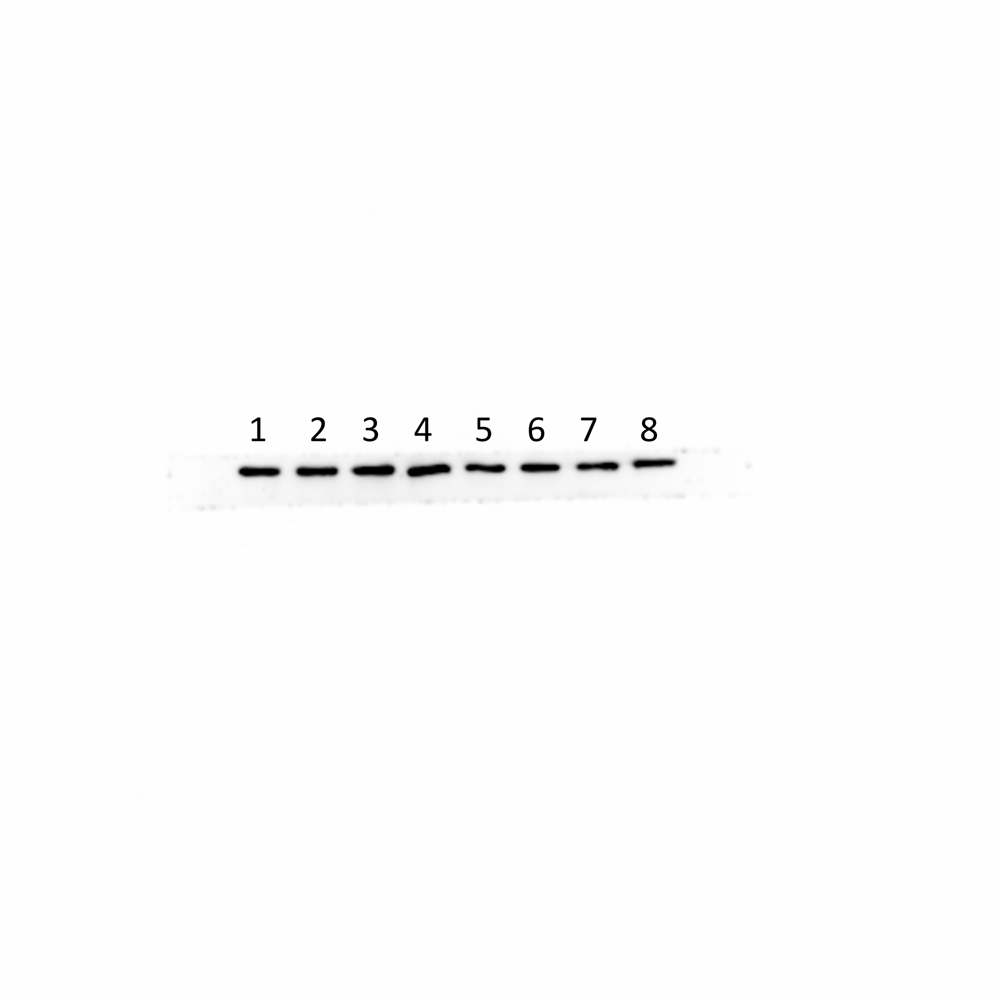

GAPDH
